# Molecular basis of allosteric regulation and pharmaceutical targeting of protein kinase Cβ

**DOI:** 10.1038/s41467-026-73413-5

**Published:** 2026-05-21

**Authors:** Anh T. Q. Cong, Taylor L. Witter, Elizabeth S. Bruinsma, Sayantani Sarkar Bhattacharya, Swaathi Jayaraman, Samuel R. Wyatt, Jasper K. Solverson, Maria B. Dugan, Jasmina Paluncic, Mary J. Kuffel, Julia R. Alvey, Huy V. Huynh, Xinyan Wu, Alan P. Fields, Akhilesh Pandey, John R. Hawse, Matthew P. Goetz, Matthew J. Schellenberg

**Affiliations:** 1 Department of Biochemistry and Molecular Biology, Mayo Clinic, Rochester, MN USA; 2 Department of Oncology, Mayo Clinic, Rochester, MN USA; 3 Department of Laboratory Medicine and Pathology, Mayo Clinic, Rochester, MN USA; 4 Department of Cancer Biology, Mayo Clinic, Jacksonville, FL USA; 5 Manipal Academy of Higher Education, Manipal, India; 6 Department of Molecular Pharmacology and Experimental Therapeutics, Mayo Clinic, Rochester, MN USA

**Keywords:** X-ray crystallography, Enzyme mechanisms, Kinases

## Abstract

Protein kinase C (PKC) isozymes are ubiquitous kinases that direct diverse cellular pathways and are important drug targets for the treatment of cancer and neurological diseases. PKCs are auto-regulating enzymes governed by phospholipid and Ca^2+^ signals via a mechanism that has remained enigmatic due to a paucity of structural information. Herein we present a series of structures of the full-length human PKCβI and PKCβII isozymes. These structures reveal the molecular basis by which PKCs maintain an auto-inhibited state, convert to a defined and ordered active conformation via a “lipid-lever” mechanism of allosteric activation, and how isoform-specific differences alter their allosteric regulatory mechanisms. We show that endoxifen, a recently identified PKCβI inhibitor, can alter the allosteric regulatory mechanism of PKCβI, providing a proof of concept for allosteric regulators of PKCs. Collectively, our data describe a foundational molecular model of second messenger-mediated allosteric regulation of PKCs that underpins PKC function, misregulation, and mechanisms of inhibition.

## Abstract (web summary)

The authors reveal how PKCβI/II enzymes switch from inactive to active states through a membrane-driven lipid-lever mechanism and show that endoxifen can allosterically inhibit PKCβ, offering distinct avenues for targeted therapeutics.

## Introduction

Protein kinase C (PKC) enzymes are a family of ten serine/threonine kinases that mediate signal transduction and as such, participate in a wide range of cellular pathways^1–5^. Altered PKC activity is implicated in many disease states, including cancer and neurodegenerative diseases^4,6–11^. PKCs are ubiquitously expressed throughout the body, yet only a variable subset of the PKC enzymes is present in each tissue type^12^. Human cells contain ten PKC proteins that are classified into the conventional, novel, and atypical families. The four conventional PKC family members (α, βI, βII, and γ) are activated upon interaction with the plasma membrane in response to diacylglycerol (DAG) and Ca^2+^ signals^13^. Although PKCα, βI, βII, and γ are >65% identical in amino acid sequence (Supplementary Fig. 1a), they perform distinct cellular functions and display varied sensitivity to phospholipid signals^14,15^. PKCβI and PKCβII isoforms are expressed from the same gene and differ by only a 50 aa C-terminal segment derived from a regulated alternative splicing event (Supplementary Fig. 1a)^16^, suggesting that even subtle sequence differences can impart altered activity. Regulation of each PKC isoform is essential for maintaining cellular homeostasis. Therefore, understanding the molecular actions of each PKC, and particularly how they respond to activating signals, is critical for deciphering their individual contributions to normal tissue homeostasis, disease processes, and the development of isoform-specific small-molecule inhibitors and treatments.

**Fig. 1:**
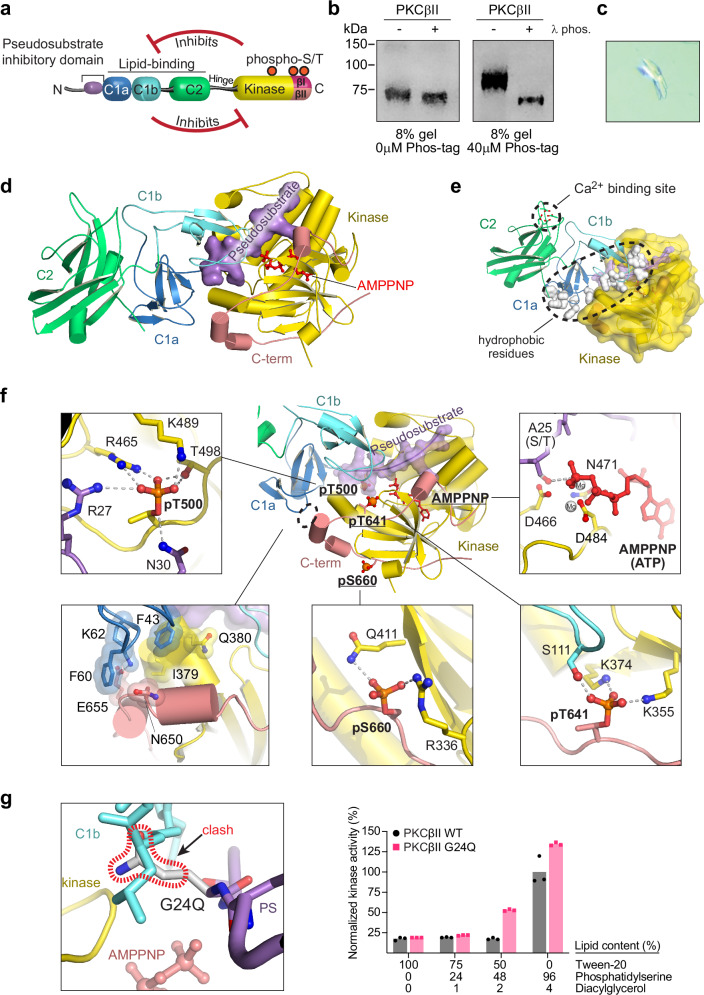
Structure of PKCβII reveals architecture of the autoinhibited state. **a** PKCβI/II domain diagram with phosphorylation sites (phospho-S/T) indicated by orange circles. **b** 8% acrylamide gel with or without Phos-tag of purified PKCβII and purified PKCβII treated with λ protein phosphatase (λ phos.). This experiment was repeated twice with similar results. **c** PKCβII crystal grown using sitting-drop vapor-diffusion method. **d** Overall structure of the PKCβII inactive state with the domains colored as in panel a. **e** In the inhibited state, many hydrophobic phospholipid-binding residues (gray) are occluded by interaction with the catalytic domain (chita yellow). **f** Molecular details of the interdomain contacts between the C1a domain, kinase domain, and C-terminal tail, and the three phosphorylation sites: activation loop (pT500), turn (pT641), and hydrophobic (pS660) with surrounding structures, and the bound AMPPNP nucleotide (red). **g** (Left) A G24Q mutation (gray) was modeled into the structure of PKCβII, which predicts a significant steric clash (red dashes) with residues from the C1b domain. (Right) Assay of kinase activity of recombinant PKCβII enzymes (WT and G24Q) in the presence of 40 μg mL^−1^ of the indicated lipid composition. *n* = 3 technical replicates. Source data are provided as a Source Data file.

The four conventional PKCs share a conserved N-terminal membrane-binding regulatory module composed of pseudosubstrate (PS), C1a, C1b, and C2 domains. The C1a and C1b domains bind DAG and PKC agonists such as phorbol esters within the phospholipid membranes; the C2 domain binds negatively charged phospholipids through a Ca^2+^-mediated interaction^15^ (Fig. 1a and Supplementary Fig. 1b). In the absence of Ca^2+^ and phospholipid signals, the N-terminal PS and membrane-binding domains inhibit the C-terminal kinase domain through intramolecular interactions to promote a closed, inactive state^17,18^. The C-terminal kinase domain acquires phosphorylation at three Ser/Thr residues during CDC37/HSP90-mediated protein folding^19,20^ that are required for kinase activity^15^. Somewhat paradoxical, phosphorylation also reduces the N-terminal domain’s affinity for phospholipid membranes^21^, indicating there is an important undiscovered mechanism of mutual regulation whereby the kinase domain can also regulate the N-terminal membrane-binding domains.

Several decades have passed since the first structural studies of PKC enzymes^22–25^. However, no structure of any full-length PKC enzyme with electron density for all domains, including the PS and C1a domains, has been solved to date, rendering a complete understanding of the molecular mechanisms of PKC regulation and activation elusive. Domain-specific structures have illuminated features of individual PKCs^24,26–28^. The most complete example to date is that of rat PKCβII^29^, which revealed the overall architecture containing C1b, C2, and kinase domains, with C1b poised to inhibit kinase activity as a potential intermediate activation state. What remains uncertain is the role of the unobserved C1a domain and fundamental processes of PKC biology, including an understanding of what holds PKC in an inactive state, what the nature of the activated state is, and the relative roles of activating signal molecules and the plasma membrane in driving the transition between the two. In the absence of such structural insights, conflicting functional models have been proposed, and the features of each PKC isoform that confer distinct activities have yet to be revealed. For example, Leonard et al. proposed a model of C1b-mediated inhibition through binding to the NFD (^628^Asn–Phe–Asp^630^) loop, which is contradicted by the model proposed by Antal et al. to contain a C2–kinase domain interface that blocks kinase activity in the absence of activating signals^29,30^. Confounding the development of such models is the fact that mutations in PKC can have pleiotropic effects. For example, the kinase-deficient PKCβII mutant K371R is also unable to dissociate from the membrane^21^, even though there is no change in the enzyme membrane-binding modalities. Thus, structural information regarding the full-length protein is critical for defining the complex mechanisms by which PKC mutations can impact multiple enzyme functions.

Despite considerable interest in developing drug candidates that modulate PKC activities for the treatment of an array of diseases, including cancer, a strategy of using ATP-competitive inhibitors has, thus far, been unsuccessful. Staurosporine and its derivatives, which target the highly conserved ATP binding site, have demonstrated limited or no antitumor activity in multiple different types of cancer despite nanomolar affinity and are prone to off-target toxicity^31–36^. The lessons learned from these molecules is that, in general, targeting PKC’s highly conserved active site is not only a suboptimal strategy, but also can simultaneously inhibit multiple PKC isoforms and other kinases, disrupting the delicate balance of kinase signaling required for cellular homeostasis^37^. In contrast, an allosteric approach, such as targeting the Phox and Bem1 (PB1) domain of PKCι, has demonstrated promising anticancer activity^38–40^. While this example highlights the therapeutic value of allosteric inhibitors, the potential for developing such inhibitors to target other disease-modulating isoforms remains understudied. Discovery of allosteric mechanisms, such as how PKCs transition between active and inactive states, is crucial for the design of future therapeutic agents with improved isoform specificity.

Here, we report the crystal structure of full-length PKCβII containing electron density for all (PS, C1a, C1b, C2, and kinase) domains, which reveals molecular interactions between the N-terminal membrane-binding domains and the C-terminal kinase domain that inhibit PKC in the absence of plasma membrane association. We also determined X-ray crystal structures of a second PKC isoform, PKCβI, in two different crystal forms, revealing how the C-terminal residues alter the regulation of and impart distinct activity to the PKCβI and PKCβII isoforms. We identified an ordered conformation that describes the kinase-active state formed upon engaging the plasma membrane in response to Ca^2+^ and DAG or phorbol esters. From these structures and supporting in vitro experiments, we propose an allosteric mechanism of PKC activation via a mechanism that we have termed the “lipid-lever.” Collectively, defining the molecular basis of PKC allosteric regulation may bridge the knowledge gap that has hampered the development of effective PKC-targeted therapies. To this end, we show that endoxifen, which is a promising therapy for endocrine-refractory ER+ breast cancer and was recently shown to deplete PKCβI protein levels^41^, is an allosteric modulator and inhibitor of PKCβI/II, thus providing a distinct approach to selective targeting of these enzymes.

## Results

PKCs require phosphorylation of three residues to yield an active protein, as well as the activity of other enzymes such as PDK1^29^ and PHLPP1 for quality control purposes^42,43^, making purification of fully active recombinant protein difficult using traditional approaches. Thus, we developed a robust human cell PKC expression system that produces abundant quantities of protein in the context of a complete repertoire of native kinases, chaperones, and quality-control proteins, using HEK293F cells and a YFP-tag system^42,44,45^ to generate recombinant PKCβII (Supplementary Fig. 1c). This expression system yields milligram-scale quantities of functional and appropriately phosphorylated PKCβII (Supplementary Fig. 1d) that migrates as a single band on phos-tag gels (Fig. 1b). We obtained crystals of PKCβII in the presence of a nonhydrolyzable ATP analogue AMPPNP and solved a 3.3 Å structure that contains two individual chains of full-length PKCβII within the asymmetric unit (Fig. 1c, d and Supplementary Table 1). Continuous electron density was visible for all four structured domains and the PS, as well as linker residues between the C1a, C1b, and C2 domains (Supplementary Fig. 2a), and portions of the linker between the C2 domain and the kinase domain (Supplementary Fig. 2b). Density was observed for the three phosphorylated residues required for proper PKC folding and enzyme function^46^ (Supplementary Fig. 2c), C1 domain Zn^2+^ ions (Supplementary Fig. 2d), and an active site–engaged Mg–AMPPNP (Fig. 1f). Altogether, 91% and 94% of the residues in each of the two chains, respectively, were observed, representing the most complete structure of any PKC isoform to date.

### Molecular basis of PKCβII autoinhibition and regulation by phosphorylation

We assigned all domains (PS, C1a, C1b, C2, and kinase) to each PKCβII chain within the asymmetric unit based on the maximum length that the disordered linkers could span (Supplementary Fig. 2e). Both chains contain a kinase active site occupied by the PS sequence, but chain A forms an intramolecular complex, and chain B forms a domain-swapped complex with the kinase domain of an adjacent monomer through a nearly identical molecular interface (Supplementary Fig. 2f–h). Thus, these structures reveal the molecular architecture of all four structured domains and the PS of PKCβII in an autoinhibited or inactive state.

In this state, the structure of PKCβII is supported by a complex network of interdomain interactions (Fig. 1e, f). The N-terminal PS residues (aa 19–30) are positioned in the substrate-binding site such that the A25 sidechain occupies a position equivalent to a Ser/Thr in a true substrate (Fig. 1f); and the remaining hydrophobic and positively charged residues engage in complementary interactions with kinase domain residues (Supplementary Fig. 3a). The C1b domain interacts with the kinase domain and PS in an orientation that sequesters the C1b hydrophobic phospholipid-binding residues and buttresses the PS in the kinase active site (Fig. 1e). We tested whether the C1b–kinase domain interface contributes to PKC regulation by generating G24Q mutant PKCβII, which was predicted to disrupt this interface (Fig. 1g). G24Q PKCβII kinase was stimulated to a greater extent than WT by phosphatidylserine-containing micelles, particularly when the concentration of phosphatidylserine was reduced (Fig. 1g), suggesting that G24Q destabilizes the inactive state of PKCβII leading to enhanced phospholipid binding and kinase activation.

Our structures also revealed that the C1a domain can form a hydrophobic interaction and salt bridges with the kinase domain and the C-terminal extension (Fig. 1f and Supplementary Fig. 3b). The DAG-binding site on C1a was solvent-accessible, but a small portion of the surrounding hydrophobic residues that would be expected to interact with the hydrophobic lipid core, including residue F43, was sequestered at the C1a–kinase interface, an interaction that would be mutually exclusive with membrane binding (Supplementary Fig. 3c). This configuration is supported by previously reported site-directed mutagenesis data showing that F43A, D382K, or E655K mutations that would disrupt the observed C1a–kinase interface resulting in a more extended conformation and increased phospholipid interactions^17,47^ (Supplementary Fig. 3d). A conserved PKC domain architecture consisting of a PS sequence immediately adjacent to a C1 domain in all PKC isozymes (Supplementary Fig. 1b) suggests the same regulatory mechanism is common to all PKC isoforms. Unlike the lipid-binding residues of the C1a and C1b domains, which are sequestered by the kinase domain, the C2 domain was shown to be supported by interactions with the C1a and C1b domains in a manner that exposes the calcium-binding site and phospholipid-binding residues to the solvent (Fig. 1d, e). This accessibility would allow the C2 domain to interact with the plasma membrane even in the proposed autoinhibited state. As such, it could function by initially recruiting PKC to the plasma membrane upon a rise in cytoplasmic Ca^2+^, consistent with a proposed prelocalization model for the C2 domain^48,49^.

In our structures, pT500 is positioned to serve a structural role mediated by salt bridges and a hydrogen bond with surrounding kinase domain residues as well as two PS residues R27 and N30 (Fig. 1f). These findings suggest that pT500 forms part of the substrate-binding site, explains both the requirement of T500 phosphorylation for kinase activity and the strong counter-selection against negatively charged residues at the +2 and +5 substrate positions^50^, which correspond to residues R27 and N30, respectively (Supplementary Fig. 3a). pT641 also formed salt bridges with two lysines located in the ATP-binding β-hairpin and α-helix active site motifs, as well as an additional hydrogen bond to S111 of the C1b domain. pS660 was located distal from the active site and stabilized the C-terminal V5 domain^46^ through interactions with Q411 and R336 residues.

Phosphorylation of these three residues has been shown to reduce the affinity of PKCs for lipid membranes despite being located outside of the membrane-binding domains^21^. Our structures indicate that the pT500–PS and pT641–C1b interactions stabilize the interdomain interfaces that sequester the phospholipid-binding surfaces of the C1a and C1b domains in an inaccessible conformation. Thus, in addition to the structural and substrate-binding roles, the PKCβII crystal structure demonstrated that pT500 and pT641 also mediate molecular interactions that maintain PKC in its inactive state, and in turn provide a molecular basis for the reduced phospholipid-binding affinity and membrane recruitment kinetics of the fully phosphorylated protein compared to that of the C1 domains alone.

### Crystal structures of PKCβI reveal active and inactive states

**Fig. 2:**
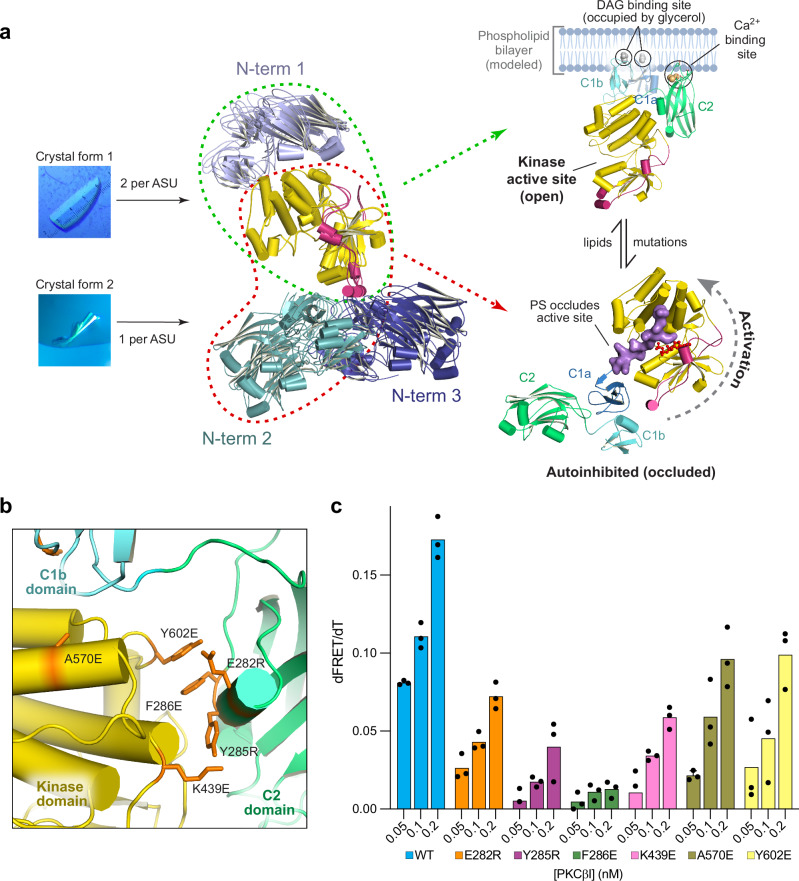
Structure of PKCβI reveals an ordered active conformation. **a** Two PKCβI crystal forms with one or two PKCβI molecules per asymmetric unit (ASU) were aligned by their kinase domain. Their crystal-packing environments are shown (N-term 1, N-term 2, and N-term 3). Two domain arrangements corresponding to the active and inhibited state are common among the PKCβI molecules. The regulatory domain phospholipid-binding residues are in a plane for the activated state (upper), while the pseudosubstrate occupies the active site in the inhibited state. Created in BioRender. Schellenberg, M. (2026) https://BioRender.com/2khvb2e. **b** Recombinant PKCβΙ protein with mutations (orange) engineered to disrupt the C1b–kinase and C2–kinase domain interactions. **c**. Mutant PKCβΙ were assayed for kinase activity in vitro using the FRET probe CKAR. *n* = 3 technical replicates. Source data are provided as a Source Data file.

PKCβI and PKCβII isoforms differ by only 50 C-terminal amino acids (Supplementary Fig. 1a) derived from an alternative splicing event that is responsive to signals such as extracellular glucose levels and signaling by AKT2 kinase^51^. Although these 50 residues are encoded by different exons, many of these residues, including the two phosphorylation sites (pT642 and pS661) present in this region, are conserved. To determine the molecular basis of isoform-specific differences, we generated recombinant PKCβI (Supplementary Fig. 4a) and co-crystallized it with AMPPNP in two crystal forms, with crystal form 1 containing two PKCβI monomers (chain A and chain B) per asymmetric unit and crystal form 2 containing one monomer (Fig. 2a; Supplementary Fig 4b; Supplementary Table 1). We again observed electron density for all four domains (C1a, C1b, C2, and kinase) together with the nucleotide AMPPNP, and phosphorylation at the corresponding βI residues T500, T642, and S661 (Supplementary Fig. 4c–e). The linkers between the C1a, C1b, and C2 domains of PKCβI were visible (Supplementary Fig. 4f) except for a gap of 10 or 11 amino acids between the C1a and C1b domains in crystal form 1 (Supplementary Fig. 4g). Similar to PKCβΙΙ, the hinge region between the C2 and kinase domain, as well as the N-terminal 18 amino acids, were disordered and not visible. However, these gaps were small enough that we could unambiguously assign the domains to individual polypeptide chains.

The most striking feature of these PKCβΙ structures is their kinase–substrate interactions. In crystal form 1, only the PS of chain A is visibly bound to the adjacent chain B kinase domain through a domain-swapped interaction (Supplementary Fig. 5a, b). The PS is disordered and not visible in chain B, nor in the PKCβΙ monomer of crystal form 2. In both instances, the arrangement of protein domains in the crystal lattice positions the C1a domain and its adjacent PS too far away from the nearest kinase domain to reach the active site, and as a consequence the kinase pocket is empty. The AMPPNP nucleotide is only present in the monomer with a PS-bound kinase, and the active site is slightly more open when the nucleotide is absent, suggesting the kinase domain could toggle between these conformations in order to release and re-bind nucleotide. In the absence of nucleotide (crystal form I, chain A), the glycine-rich loop (^351^GSFG^G354^) adopts a conformation that positions F353 in the ATP-binding site in an arrangement similar to that seen for PKCβII bound to a bisindolylmaleimide inhibitor^26^. In chain B, the glycine-rich loop adopts an extended conformation that displaces F353 from the nucleotide-binding site and unmasks three peptide dipoles that interact with the phosphates of the Mn^2+^–AMPPNP (Supplementary Fig. 5c). Although these structural features alone do not portray the biological interdomain interactions of a single PKCβΙ enzyme, they indicate that all monomers contained two conserved kinase domain elements, namely the NFD (^629^Asn–Phe–Asp^631^) motif and helix αB in conformations, which suggests the kinase domains are in the activated and catalytically competent state^52^. Thus, the unbound and peptide–nucleotide-bound states observed here likely represent states in the catalytic cycle of PKC kinases as proposed for the closely related enzyme protein kinase A^53^.

To identify the biological unit of the multidomain PKCβI enzyme and interdomain regulatory interactions, we explored the local symmetry environment in each PKCβI monomer comprised of an interface between a kinase and N-terminal domains within 4 Å in the crystal lattice. The kinase domains from all three monomers (one from crystal form 2 and two from crystal form 1) were aligned to identify common interaction modalities present among the domain arrangements that comprised the crystal lattice. Each kinase domain formed an interface with three adjacent N-terminal domain modules, and an alignment of each kinase domain and surrounding N-terminal modalities revealed that two were comprised of molecular interactions that were present for all kinase domains (Fig. 2a; N-terms 1 & 2), while the third (N-term 3) was variable among the three monomers (Supplementary Fig. 5d). We therefore hypothesized that the interfaces between N-terms 1 & 2 and the kinase domain represented physiological interactions that would be present in solution. The interface with N-term 2 yields a conformation in which the C1a and PS engage with the kinase domain similarly to PKCβII (Fig. 1d), suggesting that it represents the autoinhibited conformation of the PKCβI enzyme. The interface with N-term 1 positioned the C1b and C2 domains, such that they interact with the kinase domain C-lobe, distal from the active site in a conformation that has not been previously described. Interestingly, AlphaFold^54^ predicted a very similar structural arrangement for PKCβII and PKCα (Supplementary Fig. 5e, f) with RMSDs of 1.4 Å and 1.5 Å, respectively, compared to the active conformation of PKCβI (Fig. 2a). These findings prompted us to explore the functional role of this second PKC conformation.

### PKCβI active conformation

An unresolved mystery of PKC enzymes is how the activated state prevents the PS from re-engaging the active site. The domain arrangement comprised of the interface with N-term 1 (Fig. 2a) cannot be attributed to any previously described structure of PKC isozymes and has a few striking differences compared to the inactive conformation. No PS was bound within the kinase domain of crystal form 2, despite the active site appearing to be unobstructed by the crystal lattice. Our structures reveal that the C1b and C2 domains interact with the kinase domain C-lobe on the side opposite the active site, and the C1a domain is buttressed against the C1b and C2 domains (Fig. 2a). The C1a and C1b domains contained a bound glycerol molecule in the DAG-binding site (Supplementary Fig. 6a). Calcium was not included in the crystallization condition, but the Ca^2+^-binding site has been defined previously^27^. A prominent feature of this domain arrangement is that the phospholipid-binding surfaces of the C1a, C1b, and C2 domains align in the same plane, as would be the case when they bind or embed in a plasma membrane. This suggests that this conformation would consist of cooperative interactions with the C1a, C1b, and C2 domains, as well as the phospholipid membrane, that could compete with the autoinhibited state. We note that this arrangement would position the active site of PKC approximately 40 Å away from the plasma membrane, suggesting this could be the prime cellular region for PKC substrates to be located for phosphorylation. Furthermore, the PS would be anchored ~70 Å away from the active site and prevented from re-engaging the active site in this conformation (Supplementary Fig. 6b). The C1b and C2 domains only interact with the kinase domain C-lobe on nonmoveable surfaces, which suggests that this mode of binding would not alter the kinase domain N- and C-lobes movement between open and closed conformations that are part of the normal catalytic cycle of substrate engagement and release.

To test the hypothesis that the crystallographically observed conformation corresponds to the active conformation adopted by PKC upon binding to DAG-containing phospholipids, we employed a site-directed mutagenesis strategy and generated a panel of mutants to disrupt the observed interfaces between the kinase and C1b/C2 domains (Fig. 2b). We assayed in vitro kinase activity stimulated by Ca^2+^ and DAG-containing phospholipids using the nonradioactive FRET-based CKAR substrate. All of the mutants that were located in the C2–kinase and C1b–kinase interfaces impaired the kinase activity of PKCβI (Fig. 2c and Supplementary Fig. 6c). Since these mutations do not affect residues involved in the active site or phospholipid binding, the impaired kinase activity observed can be attributed to destabilization of the interfaces between the membrane-binding domains and the kinase domain as observed in the crystal structure. These findings indicate that PKCβI takes on a defined and ordered conformation upon association with DAG-containing membranes that unmasks the active site, generating a kinase-competent state of PKCβI.

### The lipid-lever mechanism of PKC activation

**Fig. 3:**
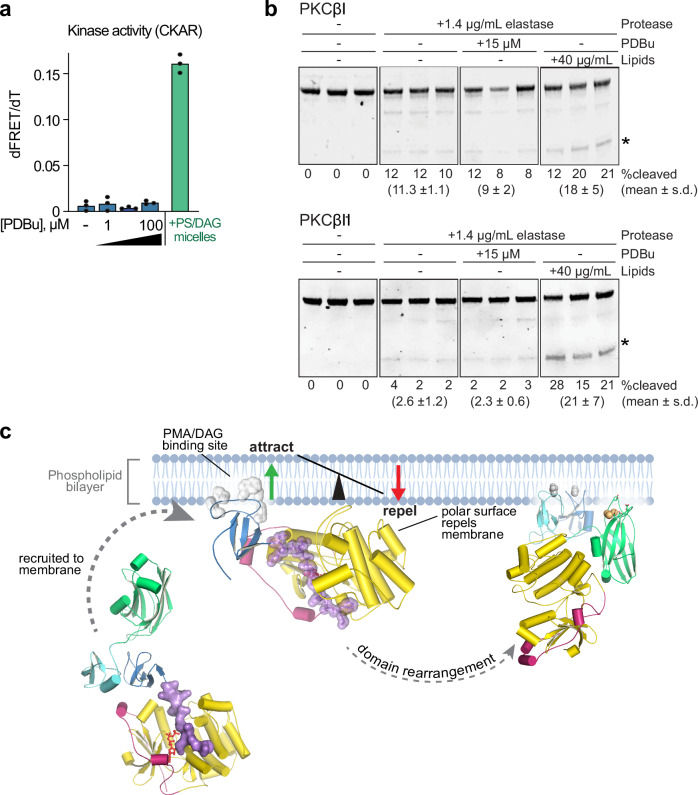
Allosteric activation of PKC is driven by binding to a phospholipid membrane. **a** In vitro CKAR assay showing the kinase activity of PKCβI in the indicated concentrations of PDBu. Addition of 40 μg mL^−1^ PS/DAG lipid vesicles to reactions demonstrates activity of fully activated PKCβI. *n* = 3 technical replicates. **b** Limited proteolysis with elastase used to probe change in PKCβI/II conformation in the presence of the indicated ligands, with quantification of cleavage product (*) as a percentage of protein from each of three replicates and the corresponding average ± SD of the values indicated at the bottom of each gel. **c**. Lipid-lever model of PKC activation. A steric clash between the phospholipid bilayer and kinase domain drives PKCβI/II into its active conformation. Created in BioRender. Schellenberg, M. (2026) https://BioRender.com/2khvb2e. Source data are provided as a Source Data file.

Another fundamental question regarding the mechanism of PKC activation is the role of the phospholipid membrane. In the presence of Ca^2+^, PKCs can be activated by a phospholipid bilayer containing DAG or other agonists such as phorbol 12,13-dibutyrate (PDBu). However, in the absence of a phospholipid bilayer, PDBu and Ca^2+^ cannot activate PKCβ kinase activity (Fig. 3a). This suggests that DAG or phorbol agonists are not the activating ligands per se, but that somehow the membrane itself drives a conformational change in PKC. Although the phospholipid-binding site of C1b is sequestered in the inactive conformation of PKCβII, the phospholipid-binding site of the C1a domain is only partially occluded (Supplementary Fig. 3b, c). The DAG/phorbol-binding cleft of the C1a domain remains solvent-accessible, such that binding a water-soluble ligand such as PDBu would not disrupt the C1a–kinase domain interface in the autoinhibited conformation (Supplementary Fig. 6d), ruling out a direct competition mechanism for the C1a domain, and instead suggesting the phospholipid membrane itself is critical for driving the conformational change that activates PKC.

We used limited proteolysis to probe the conformation of PKCβI and PKCβII in the presence of Ca^2+^ and PDBu and observed that PDBu in solution did not alter elastase cleavage even at 100 µM (Fig. 3b), indicating that phorbol binding alone is also insufficient to drive a conformational change in PKCβI and PKCβII in regions that contain an elastase-accessible cleavage site. In comparison, the addition of micelles that contain DAG embedded within the phospholipid bilayer robustly stimulates kinase activity and leads to increased elastase-mediated cleavage, consistent with a conformational change. Interestingly, modeling of the C1a domain engaged in a phospholipid bilayer revealed that the C1a–PS–kinase domain architecture observed in the crystal structure would necessitate that the kinase domain also becomes embedded into the membrane if this architecture were to be maintained (Fig. 3c). If the hydrophobic residues of the C1a domain were to engage DAG in the hydrophobic region of the membrane, it would cause a steric clash between the kinase domain and membrane that is incompatible with the inactive PKCβI/II conformation (Supplementary Fig. 6e). The C1a and PS domains would need to first undergo the slow process of dissociating^49^ and embedding in the membrane, which would prevent the otherwise rapid reassociation with the kinase domain. In this way, the lipid membrane effectively serves as a lever that holds the PS domain away from the catalytic domain, with the net effect of sequestering the PS and C1a domains from the kinase domain via a mechanism that we have termed a “lipid lever.” Additional data that support the lipid-lever model comes from reports that the C1 domains exhibit enhanced membrane binding in the absence of the kinase domain^17,49^. The kinase domain occludes a region of the C1a phospholipid-binding surface around F43 (Fig. 1f) in the inactive conformation, providing a mechanistic basis by which a fully folded kinase domain moderates membrane binding by the C1a domain, and how mutation of F43^17^ could disrupt this interface to yield a hyperactive kinase. Furthermore, the lipid-lever mechanism can be shared across PKC isoforms as the PS is always found immediately N-terminal to a C1 domain across the family of PKCs (Supplementary Fig. 1b).

### Molecular basis of isoform-specific differences between the splice variants PKCβI and PKCβII

**Fig. 4:**
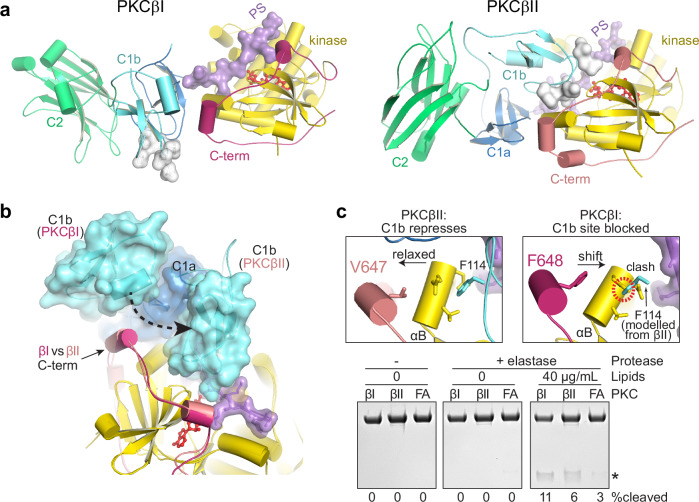
Molecular basis for differential phospholipid affinities between PKCβI and PKCβII. **a** Comparison between the structural architecture of PKCβI and PKCβII inactive conformations and the relative locations of the phospholipid-binding residues (light gray) and pseudosubstrate (purple). **b** Position of the C1b domain differs between PKCβI and PKCβII with respect to the C1a and kinase domains. **c** Intramolecular details of the residue interactions that lead to differential placement of the C1b domain, and the effect of this placement on the susceptibility of PKCβI (βI), PKCβII (βII), and PKCβI F648A (FA) to proteolytic cleavage by elastase (*). This experiment was repeated thrice with similar results. Source data are provided as a Source Data file.

PKCβI and PKCβII have different affinities for phospholipid membranes^14,17^, yet these splice variants contain identical membrane-binding domains and only differ by the last 50 amino acids of the C-terminus as a result of an alternative splicing event. Therefore, a mechanism must exist that links the variant C-termini to altered phospholipid binding. We compared the crystal structures of PKCβI and PKCβII to identify the molecular basis of isoform-specific effects. The active, nucleotide-bound conformation of PKCβI did not contain any interactions between the C-terminal extension and membrane-binding domains. Therefore, we compared the inhibited conformation of PKCβI (crystal form 1) with that of PKCβII (Fig. 4a). The most significant difference was the position of the C1b and C2 domains. The phospholipid-binding residues of the C2 domain were not occluded in either structure. In PKCβII, the C1b domain phospholipid-binding residues were sequestered through extensive contact with the catalytic domain, but in contrast, they were displaced with their phospholipid-binding residues exposed in PKCβI (Fig. 4a, b and Supplementary Fig. 6f). Enhanced accessibility of C1b phospholipid-binding residues would contribute to a stronger membrane interaction and make the PKCβI isoform more prone to activation. The C1b domain contacted only pT641 and F633 of the PKCβII V5 domain, yet PKCβI contains spatially equivalent residues (pT642 and F634) at the same locations (Supplementary Fig. 1a), suggesting it is not direct interaction between the C1b domain and PKCβI/II C-terminal tails that confers altered activity. In PKCβII, F114 of the C1b domain packs against helix αB, whereas this helix is shifted in PKCβI and partially occludes the F114 binding pocket. The αB shift could be attributed to PKCβI F648, which corresponds to V647 in PKCβII. The larger F648 residue shifts helix αB closer to the PS and the pocket occupied by F114 in PKCβII, thus preventing C1b from making close contact with this region (Fig. 4c). To evaluate this model, we engineered a PKCβI F648A mutant and probed the conformational state of PKCβI (WT and F648A) and PKCβII using limited proteolysis. In the presence of activating phospholipids and Ca^2+^, we observed elastase cleavage at a site corresponding to the hinge region (Fig. 4c). PKCβI was more sensitive to elastase than PKCβII, indicating that PKCβI more easily adopts the active conformation in the presence of phospholipids. PKCβI F648A was less sensitive to elastase, while PKCβI F648V was intermediate in sensitivity or closer to the state of WT, but the high variability in this assay could occlude smaller effects (Supplementary Fig. 6g). These data are consistent with a model whereby a smaller residue at position 648 favors a conformation with the C1b docked on the PS as seen in the PKCβII structure, while larger residues favor an equilibrium state with a displaced C1b domain. Thus, we attribute the more potent phospholipid binding of the PKCβI isoform to a shift in the position of helix αB that favors displacement of the C1b from its docked position.

### Endoxifen as an allosteric regulator of PKCβ

**Fig. 5:**
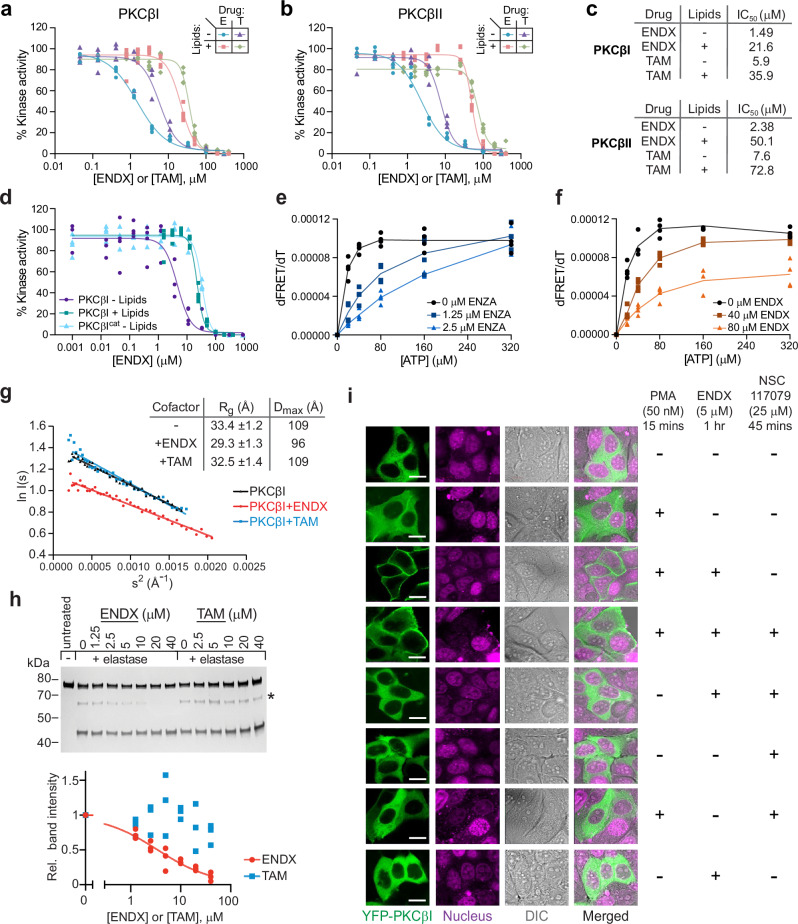
Endoxifen is an allosteric inhibitor of PKCβI. **a** In vitro Z’-LYTE kinase assay shows differences in ENDX (E) and TAM (T) inhibition of PKCβI and **b** PKCβII kinase activity under unstimulated (−lipids) compared to active (+lipids) condition. *n* = 3 technical replicates **c** The associated IC_50_ values as measured from Z’-LYTE kinase assays. **d** In vitro Z’-LYTE kinase assay showing differential ENDX inhibition against 7.5 nM unstimulated PKCβI (−lipids), 5 nM activated PKCβI (+lipids), and 0.3 nM PKCβI catalytic domain. *n* = 3 technical replicates. **e** In vitro kinase assay (CKAR) on the catalytic domain of PKCβI confirms ENZA as an ATP-competitive inhibitor. *n *= 3 technical replicates **f** ENDX is a noncompetitive inhibitor with respect to ATP. *n* = 3 technical replicates. **g** Guinier plot of SAXS data for PKCβI in the presence of the indicated ligands. **h** Limited proteolysis demonstrates dose-dependent conformational alteration of PKCβI by ENDX, but not TAM. Solid line indicates fit to four-parameter dose–response model. *n* = 3 technical replicates. **i** Confocal microscopy data show plasma membrane recruitment of YFP-PKCβI in the presence of the indicated drug treatments. Scale bar = 20 μm. Representative micrographs from three independent experiments shown. Source data are provided as a Source Data file.

PKCs can be inhibited by tamoxifen (TAM)^55^ and more potently by its metabolite endoxifen (ENDX)^41,56^. Therefore, we assessed the effects of ENDX and TAM on PKCβI kinase activity in vitro in the presence and absence of activating phospholipids. We found that ENDX inhibited PKCβI at clinically achievable^57^ concentrations (IC_50_ = 1.49 μM), while TAM required much higher concentrations (IC_50_ = 5.9 μM, *p* < 0.001), beyond that which is achievable with the FDA approved 20 mg day^−1^ dose (Fig. 5a–c). Both ENDX and TAM were less effective inhibitors in the presence of Ca^2+^ and activating phospholipid micelles. Although it is possible that phospholipids reduce the availability of ENDX in solution, the IC_50_ is similar to that of a truncated, constitutively active PKCβI consisting of only the kinase domain in the absence of phospholipids (Fig. 5d), suggesting that the active conformation of PKCβI is less sensitive to inhibition by ENDX. These results suggest weaker binding of ENDX and TAM to the kinase domain when the N-terminal domains are absent or displaced. ENDX exhibited distinctly noncompetitive inhibition with ATP in contrast to the established ATP-competitive PKCβI inhibitor enzastaurin^58^(Fig. 5e, f). In line with our previous report indicating that ENDX binds to PKCβI^41^, differential scanning fluorimetry (DSF) analysis showed that ENDX also decreases the *T*_m_ of PKCβI, further suggesting a direct interaction or ENDX-induced molecular alteration to PKCβI (Supplementary Fig. 7a). Although ligand binding is more classically associated with an increase in *T*_m_, a *T*_m_ decrease can also be induced by ligand binding^59,60^. Enzastaurin, a well-known PKCβI ATP-competitive inhibitor, decreased the *T*_m_ of PKCβI, suggesting that a reduced *T*_m_ is possibly a general effect of PKCβI inhibitors. TAM also decreased the *T*_m_ of PKCβI, but to a lesser extent, consistent with weaker PKCβI inhibition. To identify the ENDX-binding region of PKCβI, we evaluated the effect of ENDX on both the N-terminal regulatory domain and the kinase domain and found that the *T*_m_ decreased for both, but by a lesser amount than the full protein. These results suggest that both domains can interact with ENDX, indicating that ENDX inhibits PKCβI via a mechanism distinct from enzastaurin through a process that involves both the N-terminal and kinase domains. We further discovered that ENDX inhibits several other PKC enzymes with a range of IC_50_ values, but not the closely related AGC kinase PKA, which is completely unaffected by ENDX up to 100 μM (Supplementary Fig. 7b), suggesting specificity for PKC enzymes. We also evaluated inhibition by other TAM metabolites, as well as the E-ENDX isomer (Supplementary Fig. 7c). 4-Hydroxytamoxifen was an inferior inhibitor, suggesting that the 4-position hydroxyl group is not necessary for inhibition. The methylation status of the inhibitor was an important determinant of PKCβI inhibition. The mono-methyl metabolites (ENDX and N-desmethyltamoxifen) were the most potent inhibitors, while N,N-didesmethyltamoxifen (which lacks both methyl groups) had a 20-fold higher IC_50_. Collectively, these data suggest that the specific chemical structure of ENDX, rather than general hydrophobicity, is important for PKCβΙ inhibition.

We next evaluated the effect of ENDX on the solution conformation of PKCβI using small-angle X-ray scattering (SAXS). The Guinier plots indicate that the samples are monodisperse, but with low levels of aggregation in the PKCβI + TAM sample (Fig. 5g and Supplementary Fig. 7d–f). A comparison of observed scattering for the vehicle-treated PKCβI with that calculated from the crystal structure in the inactive state (Supplementary Fig. 6f) using CRYSOL reveals a good fit, with a *χ*^2^ value of 0.37 (Supplementary Fig. 7g). Modeling the unobserved dynamic residues that are not observed in the crystal structure further improved the χ^2^ to 0.18, consistent with PKCβI adopting the inactive conformation in solution. The addition of ENDX, but not TAM, yielded a detectable change in the solution conformation, evidenced by a decrease in the *R*_g_ and *D*_max_ of PKCβI protein (Supplementary Table 2). We also utilized a limited proteolysis assay, where changes in cleavage at protease sensitive sites can provide a “fingerprint” of the solution conformation of PKCβI. Elastase cleavage at a sensitive site located within the linker between the C1b and C2 domains was altered by ENDX, but not TAM, in a concentration-dependent manner, with an apparent midpoint of 3.5 μM (Fig. 5h). Collectively, these data demonstrate that ENDX alters the conformation of PKCβΙ and inhibits it via a non-ATP-competitive mechanism that is distinct from that of enzastaurin, with the potential to target additional PKC enzymes at clinically achievable concentrations.

Since PKCβI was inhibited by ENDX, we sought to determine how ENDX could influence PKCβI activity and subcellular localization by performing live-cell imaging in MCF7AC1 cells^35,61,62^ expressing YFP-tagged PKCβI. Strikingly, we found that in the presence of subsaturating levels of the PKC agonist phorbol myristate acetate (PMA), ENDX increased PKCβI accumulation at the membrane in the presence of PMA in both a time- and dose-dependent manner (Supplementary Fig 8a, b). We examined the kinetics of accumulation of YFP-PKCβI at the plasma membrane and found that ENDX pretreatment accelerated YFP-PKCβI accumulation at the plasma membrane around 4–6 min, with substantial accumulation visible at the 15-min time point (Supplementary Fig. 9). Therefore, we selected a 15-min time point for further analysis. Plasma membrane accumulation was mitigated by addition of NSC117079, which has been proposed to be a pleckstrin homology domain leucine-rich repeat protein phosphatase (PHLPP1/2) inhibitor^63^ (Fig. 5i), as well as the PP1/PP2a inhibitor okadaic acid^64^ (Supplementary Fig 10a). These data suggest that ENDX-mediated PKCβI membrane accumulation is driven by changes in the phosphorylation status of PKCβI. Therefore, diminution of PKCβI through the actions of PHLPP1/2, PP1, and PP2A phosphatases destabilizes the PKCβI/II inactive conformation (Fig. 1e, f) and releases the C1a and C1b domains to enhance membrane retention. To test this hypothesis, we compared the effect of ENDX and phosphatase inhibitors on localization of the completely unphosphorylated T500A PKCβI mutant. YFP-tagged T500A PKCβI subcellular localization was not affected by ENDX (Supplementary Fig. 10b), nor was it altered by the phosphatase inhibitors NSC117079 or okadaic acid (Supplementary Fig. 10c).

Collectively, these results suggest that ENDX inhibits PKCβI kinase activity, which alters autophosphorylation and rephosphorylation activity that would normally counteract dephosphorylation by phosphatases, ultimately resulting in increased plasma membrane accumulation. This is in line with our previous study demonstrating that ENDX treatment blocks insulin- and PMA-induced phosphorylation of PKCβI levels and leads to diminution of total PKCβI protein levels in breast cancer cells^41^. To test this model, we developed an experimental system to assess PKCβI phosphorylation using a reconstituted in vitro system with recombinant proteins. We established a phos-tag PAGE assay capable of resolving the distinct phosphorylation states of PKCβI. Recombinant PKCβI, purified from expression systems, contains three phosphorylated residues. When analyzed on a phos-tag gel, it appears as four distinct bands (Supplementary Fig. 10d, lane 2), representing species with 0, 1, 2, or 3 phosphorylated serine/threonine residues. We note that the band corresponding to the singly phosphorylated form (1P) is faint and can vary between experiments. PKCβI was then treated sequentially with PP2A to remove some phosphate groups, followed by inhibition of PP2A with okadaic acid, and then ATP was added to assess PKCβI’s ability to autophosphorylate (Supplementary Fig. 10d, lane 3). These results indicate that PKCβI can partially restore its fully phosphorylated (3P) form through autophosphorylation. However, the persistence of the 0P band after ATP addition suggests that only the 1P and 2P forms are competent for autophosphorylation and restoration of enzymatic activity. Co-incubation of PKCβI with both PP2A and ATP simultaneously leads to a stable equilibrium consisting of primarily 2P and 3P species (Supplementary Fig. 10e). Using this reconstituted system, we next evaluated the impact of ENDX on the phosphorylation/dephosphorylation balance of PKCβI (Supplementary Fig. 10f). ENDX disrupts the phosphorylation equilibrium by preventing autophosphorylation, thus shifting the balance toward dephosphorylation yielding greater amounts of the 0P PKCβI. Interestingly, treatment with the ATP-competitive inhibitor enzastaurin did not lead to accumulation of dephosphorylated PKCβI (Supplementary Fig. 10g), indicating that ENDX’s mechanism of action is distinct from that of ATP-competitive and staurosporine-derived inhibitors. Together, these findings provide a mechanistic framework for how ENDX can lead to accumulation of PKCβI at the plasma membrane.

## Discussion

In the more than 40 years that have passed since Nishizuka first reported^65^ the discovery of PKC, the molecular architecture of full-length PKC enzymes has remained undefined. A clear, molecular-level description of the interdomain interactions that comprise the autoinhibited and active forms of PKC is essential for the rational design of small molecules that alter PKC activity. In the absence of such knowledge, efforts to develop PKC inhibitors to treat a variety of diseases have thus far failed to make significant impact.

**Fig. 6:**
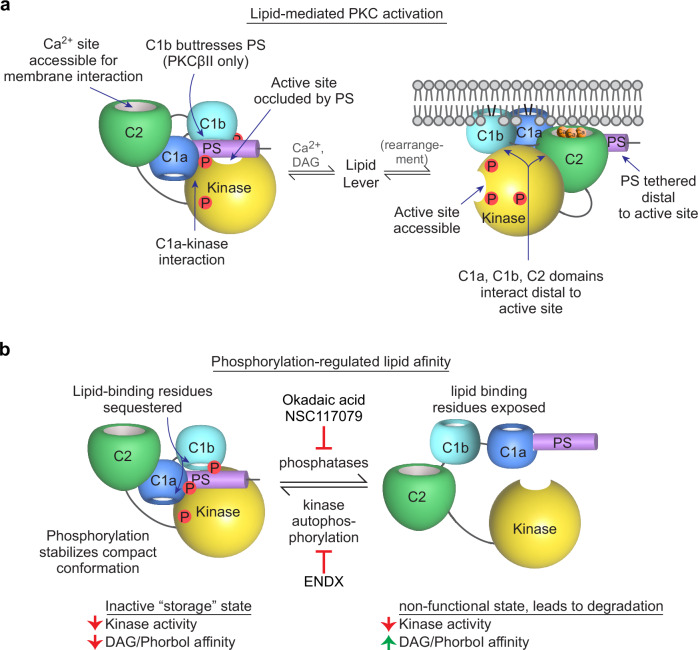
Overall model of PKC regulation. **a** Mechanism of allosteric regulation of PKC enzymes mediated by Ca^2+^, DAG/phorbol esters, and a phospholipid membrane. **b** Mechanism of phosphorylation-dependent modulation of phospholipid affinity.

Here we present the most complete structural analysis of any PKC reported to date. We have determined a series of PKCβI and βII structures that contain all of the structured domains. We have identified interdomain interactions that comprise specific states of activity of the PKCβI/II enzymes that are supported by existing data or generated additional biochemical experiments to test and validate our molecular models, although the possibility remains that the domain arrangements could be the consequence of crystal lattice packing. The models obtained from these PKCβI/II structures illuminate the conformation of key stages in the allosteric regulatory cycle of PKC enzymes, which we demonstrate is mediated by rearrangement of the regulatory N-terminal domains (Fig. 6a). In the autoinhibited state of PKCβII, the PS is bound in the active site with additional interactions between the kinase, C1a, and C1b domains that stabilize the inactive PKC conformation. The phospholipid- and Ca^2+^-binding surfaces of the C2 domain remain accessible for interaction with membranes, consistent with the “C1-inside, C2-outside” model described by Oancea and Meyer. The C-termini of the PKCβI splice variant contains a phenylalanine at position 648 which shifts helix αB and destabilizes the C1b domain inhibitory binding site on the PS, conferring enhanced or accelerated binding to phospholipids relative to PKCβII. We propose that PKC is activated by a lipid-lever mechanism upon binding to DAG which results in the C1a domain embedding within the phospholipid membrane with subsequent separation of the kinase domain from the C1a-PS complex. We note that in all PKC isoforms, the PS is always located immediately adjacent to a C1 domain (Supplementary Fig. 1b), suggesting that the lipid-lever mechanism of C1a-PS-kinase unfolding could also be conserved among all PKCs, including the novel and atypical enzymes. In the case of atypical non-membrane binding PKCs (PKCι and PKCζ), a PB1 domain-binding partner may substitute for a phospholipid membrane. A stable and ordered complex is formed when all three phospholipid-binding domains engage the plasma membrane, which positions the PS distal from the active site and prevents re-inhibition until dissociation from the membrane. This addresses another conundrum with the existing “beads-on-a-string” model^66^ for the activated state of PKC, namely the mechanism that prevents the PS from re-engaging the kinase active site in the presence of DAG and Ca^2+^. We demonstrate here that the ordered activated state (Fig. 2a) tethers the PS distal from the active site, and thus it is unable to re-engage and inhibit the kinase domain while associated with a plasma membrane.

The lipid-lever activation model (Fig. 3c) is distinct from previously described models of PKC activation and remarkably resolves a number of conflicts and unexplained phenomena in the literature. The molecular basis of reduced PKC membrane affinity upon acquisition of the three constitutive phosphorylations relies on the contribution of phosphates to sequester the phospholipid-binding surfaces of the C1a and C1b domains against the kinase domain (Fig. 1e, f). A near-complete structure of PKCβII described by the Hurley group^29^ proposed a mechanism by which the C1b domain clamped onto a C-terminal helix to regulate the kinase domain in a manner that is competitive with DAG binding. Such a model is not compatible with the C1a–PS–kinase domain arrangement that we observed in our structure of the inhibited state, as the ~14 aa linker between the C1a and C1b is not long enough to span the ~75 Å distance between the C1a in the location observed in the PKCβII structure herein and the C1b location in the Hurley structure^29^. However, as noted in their manuscript, the Hurley model may represent an intermediate state in which the C1a domain has engaged the lipid membrane but the C1b domain has not. Interestingly, they also observed a C2–kinase domain interface in their crystal lattice, which they hypothesized could be part of the active PKC conformation. This same interface is observed in the active full-length PKCβI crystal structures described herein and is supported by mutational analysis of C2–kinase interface (Fig. 2). On the other hand, an alternative interpretation of the C2 domain arrangement was proposed^30^, in which the C2 domain could regulate the kinase by blocking the active site in the absence of membrane binding. However, in this arrangement, the C2 domain would clash sterically with the PS observed in the inactive PKCβI/II states. Indeed, the structure of the inactive state of PKCβII in this study reveals that conserved residues can perform multiple functions, confounding the interpretation of their roles in mutagenesis studies. For example, hydrophobic phospholipid-binding residues of the C1a and C1b domains are involved in intramolecular contacts in the inhibited state (Fig. 1e, f), implying that altering them can both destabilize the inactive state, impairing membrane binding and PKC activation. The net balance of these two effects will determine the experimental outcome, and therefore the structural models presented in this report are an invaluable guide for interpreting the effects of altered PKC residues as well as posttranslational modifications.

The inhibited states of PKCβI and PKCβII both have the C2 domain located distal from the kinase domain, which raises the question as to how the C2 domain contributes to the regulation of PKCs. The phospholipid-binding residues and Ca^2+^ sites of the C2 domain are the only membrane-binding regions that are unmasked and accessible in the inhibited state, whereas the phospholipid-binding residues of the C1a and C1b domains are sequestered at the kinase surface (with the possible exception of PKCβI C1b). C2–membrane interactions may therefore rapidly prelocalize PKCs to the membrane to provide adequate time for the C1 domains to undergo the slower rearrangement that embeds their phospholipid-binding residues into the plasma membrane, leading to kinase activation.

Ultimately, the ability to alter PKC regulation, or correct misregulation with small-molecule inhibitors, is of great interest for improving human health. By defining the molecular steps that underlie PKC regulation, we have revealed that there are molecular interfaces within PKC that can be targeted using allosteric inhibitors. Allosteric inhibitors of the PB1 domain of PKCι have already shown promise as a therapeutic approach to target multiple forms of cancer^67^. Here we report a role for ENDX as an allosteric inhibitor of PKCs that changes the conformation of PKC. In cells, ENDX treatment leads to accumulation of PKCβI at the membrane, an effect that appears to be driven by altering the balance between autophosphorylation and the activity of PHLPP1/2 and PP1/PP2a phosphatases, which ultimately leads to dephosphorylation and subsequent degradation and downregulation of PKCβΙ^41^ (Fig. 6b). However, a recent report has proposed that PHLPP2 is a pseudophosphatase, and therefore a possibility should be considered that the effects of NSC117079 could be mediated by proteins other than PHLPP2^68^. ENDX is currently being developed for ERα-positive breast cancer, with demonstrated preclinical^69^ and clinical^57,70^ antitumor activity that is superior to tamoxifen, but only at higher concentrations that additionally target non-ER proteins such as PKCβI. Furthermore, PKC misregulation has been linked to bipolar disorder^71^, and a randomized trial demonstrated that ENDX significantly reduced total mania scores^72^, via a mechanism that was proposed to include modulation of PKC activity. Guided by the complete structures of PKCβI described here, future optimization of the ENDX molecule, or other small molecules, may lead to the development of more selective and potent inhibitors of PKCs.

In summary, PKCβI/II are multidomain proteins with interdomain molecular contacts that maintain the enzyme in an inactive conformation. Ca^2+^ and DAG signals, in concert with a phospholipid membrane, drive a conformational change in PKCβII via a lipid-lever mechanism, to activate the protein. This mechanism is likely common to all conventional PKC enzymes, and potentially the novel and atypical PKCs as well, with each enzyme responding to activating signals via variations in amino acid sequences as is the case for PKCβI/II (Fig. 4). Finally, the multidomain allosteric mechanism of PKCβI/II, and other PKC enzymes, is targetable by molecules that can bind in a location distinct from the ATP-binding active site, representing a strategy for developing a generation of PKC-targeted therapeutics.

## Methods

### Reagents, cell lines, antibodies, plasmids

HEK293F cells (Thermo Fisher) and MCF7AC1 cells were grown in DMEM with 10% (v/v) fetal bovine serum (Gibco), 50 U µL^−1^ penicillin, 50 µg mL^−1^ streptomycin, and 0.5 mmol L^−1^ sodium pyruvate at 37 °C in 5% CO_2_ atmosphere for adherent growth. Suspension cultures of HEK293F cells were grown in CDM4HEK (Cytiva) medium supplemented with 20 mM glutamine, 12 U µL^−1^ penicillin, and 12 µg mL^−1^ streptomycin at 37°C in 8% CO_2_ atmosphere at 135 rpm in a Multitron shaker incubator (HT Infors) for suspension growth. Antibodies used in this study: anti-PKCβ phospho-T500 (Abcam ab5817, lot GR173447-1), anti-phospho-T642 (Abcam ab75657, lot GR29222-17), and anti-phospho-S661 (Abcam 192184, lot GR224655-1), and were all used at a 1:1000 dilution for Western blotting. DNA encoding PKC constructs was cloned into pMCentr2 (DNASU) and recombined into mammalian protein expression vector pcDNA6.2/N-YFP-Dest using LR Clonase II (Invitrogen). Mutant PKCβI plasmids were generated using the QuickChange kit (Stratagene). Primer sequences are listed in Supplementary Table 3.

### Expression and purification of PKCβI and PKCβII

Recombinant plasmid was mixed with 1 mg mL^−1^ polyethyleneimine, pH 7.5 (Polysciences, Inc.) in a 1:3 ratio to form a DNA–PEI mixture used to transfect HEK293F cells in suspension culture using the procedure previously described^44,45,73^. Proliferating HEK293F cells were inoculated in fresh HyClone HyCell Transfx-H (Cytiva) medium supplemented with 200 mM glutamine (Thermo Fisher) and 0.075% pluronic (Gibco 24040-032) at a density of three million cells per mL immediately prior to transfection. A 500 mL transfection was performed by preparing 1.2 mg of plasmid DNA in 5 mL of the same medium in one tube and 1.4 mg of PEIMAX 40K linear PEI (Polysciences) in 35 mL of medium in a second tube. The DNA- and PEI-containing tubes were rapidly mixed for 10 s by pipetting vigorously, then added to the culture flask containing the HEK293F cells. Transfected cells were grown for 72 h at 37°C, while cell count and protein expression (GFP) level were monitored every 24 h. PKCβ-expressing culture was spun down at 5000 × *g* for 10 min, followed by resuspension of pelleted cells in 1× PBS containing 0.1× Roche EDTA-free protease inhibitor cocktail (Sigma). Centrifugation step was repeated to obtain cell pellet that was used directly for protein preparation or frozen down at −80°C for storage.

Thawed HEK293F cells expressing PKCβ were lysed at 4 °C with a lysis buffer solution^29^ consisting of 50 mM Tris pH 7.4, 300 mM NaCl, 50 mM NaF, 5 mM sodium pyrophosphate, 10 mM β-glycerol phosphate, 1 mM TCEP, 2 mM benzamidine, 2 μg mL^−1^ leupeptin, 0.5 mM sodium orthovanadate, 0.5% CHAPS, and 1:100 protease inhibitor cocktail (Sigma P8849) as described. Lysate was sonicated with a Branson sonicator at 50% power for 10 s (×2) and spun down in a Lynx 4000 centrifuge (Thermo Fisher) at 15,000 × *g*, 4°C for 10 min. The clarified lysate was bound and recycled 3× over a GFP-enhancer nanobody-linked NHS Sepharose (Cytiva 45002965) resin bed^44,45,73^ equilibrated in lysis buffer. Protein-bound resin was washed in 3× resin volume with the same lysis buffer. TEV cleavage of the YFP tag was completed overnight by incubating the resin with 1.5× resin volume TEV cleavage buffer containing 50 mM Tris pH 7.4, 50 mM NaF, 5 mM sodium pyrophosphate, 10 mM β-glycerol phosphate, 1 mM TCEP, 0.5 mM sodium orthovanadate, and 0.25% CHAPS, supplemented with 0.09 mg mL^−1^ TEV protease. For cleavage of PreScission site–containing constructs, PreScission protease was substituted for TEV protease. Cleaved protein was eluted from the resin using buffer without TEV protease, and the presence of protein in eluted fractions was monitored using a Coomassie-stained SDS-PAGE gel.

Following purification, PKCβ proteins were polished on an ÄKTA go FPLC system (Cytiva) to remove TEV protease and further purify protein for biochemical assays and crystallization. TEV-eluted protein was diluted (1:3) with a low-salt buffer containing 50 mM Tris pH 8.0 and 1 mM TCEP prior to being loaded onto a HiTrap Q HP anion exchange column (Cytiva). A salt gradient was introduced by the gradual addition of buffer supplemented with 1 M NaCl at a flow rate of 5 mL min^−1^. Gradient fraction elution continued to a final of 60% or 600 mM NaCl. Eluted fractions containing PKCβ were combined and concentrated to 0.5 mL using a 10K cutoff centrifugal filter (Sartorius Vivaspin), pretreated overnight with 3% PEG 3350 (Jena Biosciences) at 4°C to avoid protein loss through binding to filter. Concentrated protein was subjected to final purification on a Superdex 200 Increase 10/300 GL column (Cytiva) using 20 mM Tris pH 8.0, 100 mM NaCl, 2 mM MgCl_2_, and 1 mM TCEP at a flow rate of 0.5 mL min^−1^. All FPLC elution fractions were monitored for protein presence using Coomassie-stained SDS-PAGE gel. Fractions containing PKC were pooled and concentrated to 10 mg mL^−1^ using a 10K centrifugal filter (Sartorius Vivaspin) for crystallization, or buffer-exchanged into storage buffer (20 mM Tris pH 8.0, 100 mM NaCl, 1 mM MgCl_2_, 0.5 mM TCEP, 25% (v/v) glycerol) and stored at −80°C prior to use in kinase assays. Typical yield of final, purified PKCβI/II proteins (77 kDa) was 3–6 mg L^−1^ of culture.

### Lambda phosphatase reactions

To generate dephosphorylated PKC, 7 μg PKC protein were incubated at 37 °C for 2 h with 1400U lambda phosphatase (New England BioLabs P0753S) and its 1× reaction buffer according to the manufacturer’s protocol. As a control, 7 μg PKC protein was incubated under the same conditions in the absence of phosphatase. After the incubation period, loading dye was added and the reactions were heated to 75°C for 5 min prior to visualization via 8% SDS-PAGE with 0 μM Phos-tag or 40 μM Phos-tag (APExBIO).

### Phos-tag gel

To resolve phosphorylated protein species, SDS-PAGE was performed using a Phos-tag acrylamide gel. The resolving gel (6%) was prepared by supplementing a standard acrylamide/bis-acrylamide solution with Phos-binding acrylamide reagent (APExBIO), and MnCl_2_ to concentrations of 12.5 and 25 mM, respectively. Polymerization was initiated by the addition of ammonium persulfate (APS) and TEMED. The stacking gel (4.5%) was prepared without Phos-tag or MnCl_2_. Following electrophoresis, gels were silver-stained using the standard protocol. Gels are visualized using an iBright system (Thermo Fisher) and analyzed using FIJI.

### Crystallization and structure determination of PKCβI and PKCβII

Crystals of PKCβI and PKCβΙΙ were grown using the sitting-drop vapor diffusion method by mixing 200 nL of precipitant with 200 nL of protein mixture (PKCβI or PKCβII with 1 mM AMPPNP). Crystals of PKCβI crystal form 1 and 2 grew in 200–300 mM sodium citrate pH 7–8 and 8%–10% (w/v) PEG 3350. Crystals of PKCβII grew in 100–200 mM magnesium chloride, 0.1 M MES pH 6.5, and 6%–10% (w/v) PEG 8000. Crystals grew over a period of 2–6 weeks at 4 °C and were transferred to a cryoprotectant containing crystallization condition supplemented with 25% (v/v) glycerol, then flash frozen in liquid nitrogen. For manganese soaks, the cryoprotectant also contained 1 mM MnCl_2_. X-ray diffraction datasets were collected at the NE-CAT beamlines (24-C and 24-E) at the Advanced Photon Source. X-ray diffraction data were processed and scaled using the HKL2000 suite^74^. Structures were solved via molecular replacement using the C1b, C2, and kinase domains from PDB entry 3PFQ^29^ as search models using the PHENIX PHASER^75^. The C1a domain and other missing regions were built manually using COOT^76^, followed by refinement against the high-resolution datasets with PHENIX^77^ to produce the final models. Figures were generated using PyMOL (Schrodinger Scientific). Except where indicated otherwise, a consistent domain coloring scheme was used for each protein domain as outlined in Supplementary Fig. 1a.

### Limited proteolysis

0.4 mg mL^−1^ PKC protein was digested with 1.4 μg mL^−1^ elastase (Promega) in the presence and absence of activating factors (40 μg mL^−1^ lipids and 82 μM Ca^2+^). Additional reaction components or PKC-interacting molecules were added in the concentrations indicated. Reactions were incubated at room temperature for 45 minutes. Protease activity was quenched by adding SDS reducing dye and heating at 70 °C for 10 min. The full mixture was loaded onto a SurePAGE Bis-Tris 4–12% gel (Genscript) and stained with Coomassie blue to visualize proteins. Gels were photographed using an iBright instrument (Thermo Fisher). Band intensities for the cleaved and uncleaved protein were quantified using ImageJ, and the percentage cleavage was determined by dividing the intensity of the cleaved band by the sum of the cleaved + uncleaved bands. IC_50_ values were calculated by fitting the percentage cleavage as a function of ENDX concentration to a four-parameter dose–response model using GraphPad Prism.

### Expression and purification of CKAR

pRSET-B-CKAR^28^ (Addgene) was transformed into JM109 DE3 *E. coli* (Promega)^78^, which contains the recA1 mutation that is important for maintaining the stability of tandem fluorescent protein reporters. Cultures of pRSET-B-CKAR in JM109 DE3 were grown in 2 L of Terrific Broth (Research Products International), supplemented with 300 μL of Antifoam 204 (Sigma-Aldrich) in a LEX-48 Bioreactor (Epiphyte3) at 37 °C, until they reached an optical density (OD_600_) between 3.0 and 4.0. CKAR protein expression was induced by the addition of 50 μM of isopropylthio-β-galactoside (IPTG, GoldBio) for 18 h at 16 °C. The bacterial cell pellet was harvested by centrifugation at 6000 × *g* for 20 min at 4 °C and frozen at −80 °C.

Cell pellets were thawed and lysed in Ni running buffer (20 mM Tris pH 7.5, 300 mM NaCl, 0.5 mM TCEP, 10 mM imidazole) supplemented with 0.1 mg mL^−1^ of lysozyme and a 1/2000 dilution of ethanol-saturated phenylmethylsulfonyl fluoride (PMSF, GoldBio) on ice for 30 min. Lysate was sonicated at 80% power for three 30-s intervals using a Branson 250 sonifier. Sonicated lysate was centrifuged at 25,000 × *g* for 30 min, and soluble fraction was passed over Ni-NTA resin (Qiagen) pre-equilibrated with lysis buffer. The resin was washed six times, each with one column volume of lysis buffer, and CKAR was eluted with Ni running buffer supplemented with 250 mM imidazole. Protein was precipitated out of solution by the addition of 2× volume of 4 M ammonium sulfate, and pellets were collected after centrifugation at 25,000 × *g* for 30 min. The protein pellets were redissolved in 2 mL of Milli-Q water and polished on a Superdex 200 16/60 column (Cytiva) in 20 mM Tris pH 7.5, 300 mM NaCl, and 0.5 mM TCEP buffer. Protein elution was monitored by absorbance at 280, 460, and 520 nm to identify fractions with full-length CKAR and both YFP and CFP modules. CKAR was further polished by ion-exchange chromatography on a HiTrap Q HP column (Cytiva) using a 0%–50% gradient between low-salt (20 mM Tris pH 7.5, 3 mM DTT) and high-salt buffers (20 mM Tris pH 8.0, 1 M NaCl). CKAR was concentrated and buffer-exchanged into PKC storage buffer (20 mM Tris pH 8.0, 100 mM NaCl, 2 mM MgCl_2_, 1 mM TCEP, and 25% (v/v) glycerol) using an Amicon 10K concentrator (Millipore). The final CKAR product was quantified using *A*_520nm_ and an extinction coefficient of 70,000 M^−1^cm^−1^ for YFP.

### CKAR kinase assay

Lipid vesicles were prepared fresh by dissolving 10 mg mL^−1^ bovine brain phosphatidylserine (PS) (Avanti) and/or 1 mg mL^−1^ 1,2-dioleoyl-glycerol (DAG) (Avanti) in chloroform. Lipids were mixed at the indicated ratio, and chloroform was evaporated with a stream of dry air, then lipids were redissolved in water to a final concentration of 40 μg mL^−1^, vortexed for 60 s, and sonicated three times for 30 s at 10% power on a Branson 250 sonifier with a microtip (12840498). Concentrated PKC protein was diluted into storage buffer containing 20 mM Tris pH 8.0, 100 mM NaCl, 2 mM MgCl_2_, 1 mM TCEP, 25% (v/v) glycerol and 1 mg mL^−1^ BSA to create a 10× stock based on final assay concentration. Kinase reaction mixtures (100 μL) contained 20 mM HEPES pH 7.5, 2 mM MgCl_2_, 1 μM CKAR, 10 nM PKCβ protein, and indicated concentrations of ENDX and ATP. Reaction mixtures were preheated at 30°C, and ATP was mixed in last to initiate the reaction, just prior to placing the reaction plate in a CLARIOstar Plus plate reader (BMG Labtech). Förster resonance energy transfer (FRET) was measured every 5 min using a 434-16 excitation filter with 476-16 and 528-16 emission filters^28^ at 30°C. Control reactions without PKC were used as background measurements to correct for fluorophore decay throughout the experiment. The FRET ratio was calculated by dividing the emission measurement at 476 nm by 528 nm emission using the MARS software (BMG Labtech). PKC kinase activity was plotted as the change in FRET ratio over time (dFRET/d*T*) corresponding to inhibitor concentrations using Prism 9 (GraphPad).

### Z′-LYTE kinase assay

Kinase activity was measured using the Z′-LYTE Kinase Assay Kit – Ser/Thr 7 Peptide (Thermo Fisher) following the manufacturer’s protocol. The reaction mix contained 250 mM HEPES pH 7.5, 50 mM MgCl_2_, 5 mM EGTA, 0.05% Brij-35, 40 μg mL^−1^ PS:DAG lipids, PKC protein at the indicated concentration, drug serial dilution at the respective concentration, 40 μM ATP, and 2 μM Z′-LYTE Ser/Thr 7 peptide substrate. The peptide substrate and ATP mixture were added last to initiate the reaction. For reactions without lipids, the 40 μg mL^−1^ lipids were substituted with Milli-Q H_2_O. Reactions were incubated at room temperature for 1 h. The development solution, created using the manufacturer’s instructions, was added and mixed with the kinase reaction prior to placing the plate in the CLARIOstar Plus plate reader (BMG Labtech). The extent of phosphorylation of the peptide substrate is calculated through the ratio of the coumarin emission at 445 nm to the fluorescein emission at 520 nm after a 1-h incubation with development solution. Kinase activity is plotted as percent activity to the corresponding drug concentration. To determine inhibitor IC_50_ values, data were fitted to a four-parameter dose–response model using Prism 9 (GraphPad). In cases where the IC_50_ could not be accurately determined due to precipitation of ENDX at very high concentrations, the model was constrained to a bottom value of 0%, and the resulting estimated IC_50_ value is reported. Extra sum-of-squares *F*-test analysis was used to calculate *P* values between different drug treatments.

For the G24Q assay, 40 μg mL^−1^ 96:4 PS/DAG was prepared as described for the CKAR assay, then diluted by addition of 0.04% Tween-20 to achieve the indicated concentrations. These solutions were then vortexed for 30 s and sonicated three times for 30 s at 10% power on a Branson 250 sonifier with a microtip (12840498). All final lipid mixtures were placed on the thermomixer for 1 h at 37°C immediately prior to use in the Z′-LYTE activity assay. Kinase activity was normalized based on the average protein absorbance values for the 100% lipid condition. Data visualization and statistical analysis were done using Prism 9 (GraphPad).

### Expression and purification of PKA

DNA encoding PKA catalytic domain (Addgene 14921) was transformed into Rosetta2 cells (EMD) and inoculated in a 2 L culture of Terrific Broth (Research Products International) supplemented with 300 μL Antifoam 204, 100 μg mL^−1^ carbenicillin, and 34 μg mL^−1^ chloramphenicol. Culture was grown using a LEX-48 Bioreactor (Epiphyte) at 37 °C, and protein expression was induced with 100 µM IPTG overnight at 16 °C. *E. coli* culture was pelleted and resuspended in lysis buffer (20 mM Tris pH 7.5, 300 mM NaCl, 10 mM imidazole, and 0.5 mM TCEP) supplemented with 10 mg of lysozyme (GoldBio) and 20 μL of saturated PMSF in ethanol (GoldBio). Cell lysate was incubated on ice for 30 min with occasional mixing and then sonicated using a Branson 250 sonicator at 80% power in five 15-s intervals. Clarified lysate was centrifuged at 25,000 *× g* for 30 min and passed over a Ni-NTA column equilibrated in lysis buffer. Ni-NTA resin was washed with five column volumes lysis buffer and eluted with lysis buffer supplemented with 250 mM imidazole. Protein-containing fractions were detected by a color change in a Bradford Assay (99 μL Bradford reagent, 1 μL elution fraction), pooled, and precipitated with two volumes of 4 M ammonium sulfate followed by centrifugation at 25,000 *× g* for 30 min at 4 °C. Precipitated protein was dissolved in 3 mL Milli-Q water and loaded onto a Superdex S200 size exclusion column equilibrated in 20 mM Tris pH 7.5, 300 mM NaCl, and 0.5 mM TCEP. Fractions were analyzed by SDS-PAGE gel, and PKA-containing fractions were pooled and diluted 1:5 in Milli-Q water. The pH of the dilution was adjusted to pH 6 by addition of MES powder and loaded onto a Resource 15S cation-exchange column (Cytiva) equilibrated in running buffer (20 mM NaH_2_PO_4_ pH 6.0, 0.1 mM TCEP) and eluted with a linear gradient of 0%–100% 1 M NaCl in 20 mM NaH_2_PO_4_ pH 6.0. To increase the pH in PKA-containing fractions 20 mM Tris pH 8.0 was added to pooled protein, and the protein was then concentrated by Amicon ultrafiltration (Millipore). Concentrated protein was run over a Superdex 200 Increase column (Cytiva) equilibrated in running buffer (20 mM Tris pH 7.5, 300 mM NaCl, and 0.5 mM TCEP). Purified protein was analyzed by SDS-PAGE gel and buffer-exchanged during Amicon centrifugation to PKC storage buffer (20 mM Tris pH 8.0, 100 mM NaCl, 2 mM MgCl_2_, 1 mM TCEP, and 25% (v/v) glycerol).

### Small-angle X-ray scattering

PKCβI was purified and buffer-exchanged into SAXS buffer (20 mM Tris pH 8.0, 100 mM NaCl, 2 mM MgCl_2_, 1 mM TCEP, and 1% (v/v) glycerol) using a Superdex Increase 10/300 column (Cytiva). Aliquots of the highest concentration fraction were supplemented with ENDX or TAM to a final concentration of 80 μM, or an equivalent volume of water was added to the inhibitor-free sample. Samples were concentrated briefly with 0.5 mL 10K centrifugal filters (Amicon) that had been preblocked and washed (incubated overnight at 4°C in SAXS buffer supplemented with 3% (v/v) PEG400, followed by 3× washing in SAXS buffer with 10 s spins in the centrifuge to remove any remaining PEG). Samples were concentrated briefly by centrifugation at 4°C and 8000 × *g* to 17 μM concentration, and concentrator flowthrough was used to prepare diluted samples of 8.5 and 4.2 μM, as well as the buffer subtraction control. SAXS data were collected at the SIBYLS beamline at the Advanced Photon Source using the mail-in service. Data frames were merged using the SIBYLS Frameslice software as follows: frames 1–8 for *S* = 0.008742–0.091598, frames 1–15 for *S*= 0.092358–0.175208, and frames 1–33 for *S* = 0.175968–0.446450. Inspection of Guinier plots for the 17 μM samples indicated a small but detectable amount of protein aggregation, whereas none was detectable for the 8.5 and 4.2 μM samples. Data from the 8.5 μM samples were used for analysis in PRIMUS (version 3.03) from the ATSAS suite^79^. *R*_g_ was determined using the AutoRg function in PRIMUS. *D*_max_ and real-space parameters were determined using GNOM. Calculation of theoretical SAXS data and comparison to the observed scattering for PKCβI was performed using CRYSOL. Modeling of flexible residues that were not observed in the crystal structure was performed using EOM^79^, by fitting against the full range of observed scattering with the position of all residues observed in the crystal structure fixed.

### AlphaFold models

Predicted structural models of full-length PKCα and PKCβI were obtained from AlphaFold^54^, respectively. Structure of PKCα was obtained as AF-P17252-F1-model-v4 and AF-P05771-F1-model-v1 for PKCβI.

### Live cell imaging

MCF7AC1 cells that stably expressed YFP-PKCβI were generated by transfecting plasmid DNA using Lipofectamine 2000 (Thermo Fisher) according to the manufacturer’s instructions, and then following the protocol described in the “Expression and purification of PKCβI and PKCβII” section for generating a stable expression cell line. For microscopy, YFP^+^ cells were seeded in 35-mm glass-bottom microwell dishes (MatTek Corporation) for at least 24 h. Subsequently, cells were incubated with ENDX 1 h prior to imaging, followed by addition of PMA 15 min prior to imaging, as indicated in figure legends. Where indicated, phosphatase inhibitors NSC117079 (MedChem Express) or okadaic acid (Cayman Chemical) were added 45 min prior to imaging. Successively, NucRed Live 647 Reagent (Invitrogen) was added for live-cell nuclear staining and visualized using a Zeiss-LSM 780 confocal microscope. Confocal images were processed using Carl-Zeiss Blue/Black ZEN 3.0 SR software. FIJI ImageJ was used to prepare cropped panels and timecourse montages using linear LUT that covers the full range of the data, and colors as indicated in figure labels.

### Differential scanning fluorimetry (DSF)

DSF was performed using a Protein Thermal Shift Dye Kit (Thermo Fisher) according to the manufacturer’s protocol. The reaction mixture consisted of 1 μg of PKCβI FL WT. Data were collected using a CFX96 Touch Real-Time PCR System (Bio-Rad). Thermal stability was assessed for PKCβI FL WT alone and in the presence of 40 μM of endoxifen, tamoxifen, or enzastaurin. Additionally, DSF experiments were conducted with 1 μg of unbound PKCβI N-terminal and C-terminal domains, both alone and with 40 μM of endoxifen. All data were analyzed using Bio-Rad CFX Manager (Bio-Rad Laboratories, Inc.).

### Quantification and statistical analysis

Standard statistical analyses were used to distinguish significant from nonsignificant results as indicated.

### Reporting summary

Further information on research design is available in the Nature Portfolio Reporting Summary linked to this article.

## Supplementary information

Supplementary Information

Reporting Summary

Transparent Peer Review file

## Source data

Source data

## Data Availability

All unique/stable reagents generated in this study are available from the lead contact with a completed materials transfer agreement. Atomic coordinates and structure factors have been deposited in the Protein Data Bank (www.rcsb.org) under accession codes 8SE1 (PKCβII), 8SE4 (PKCβI form 1), 8SE3 (PKCβI form 2), and 8SE2 (PKCβI-Mn complex). Source data are provided as a Source Data file. Source data are provided with this paper.

## References

[1] Harper, M. T. & Poole, A. W. Diverse functions of protein kinase C isoforms in platelet activation and thrombus formation. *J. Thromb. Haemost.* **8**, 454–462 (2010). 20002545 10.1111/j.1538-7836.2009.03722.x

[2] Kawakami, T., Kawakami, Y. & Kitaura, J. Protein kinase C beta (PKC beta): normal functions and diseases. *J. Biochem.* **132**, 677–682 (2002). 12417015 10.1093/oxfordjournals.jbchem.a003273

[3] Gomez, J. et al. The zeta isoform of protein kinase C controls interleukin-2-mediated proliferation in a murine T cell line: evidence for an additional role of protein kinase C epsilon and beta. *Exp. Cell. Res.* **218**, 105–113 (1995). 7737351 10.1006/excr.1995.1136

[4] Leitges, M. et al. Immunodeficiency in protein kinase cbeta-deficient mice. *Science* **273**, 788–791 (1996). 8670417 10.1126/science.273.5276.788

[5] Callender, J. A. & Newton, A. C. Conventional protein kinase C in the brain: 40 years later. *Neuronal Signal.* **1**, NS20160005 (2017). 32714576 10.1042/NS20160005 PMC7373245

[6] Tagawa, K. et al. Comprehensive phosphoproteome analysis unravels the core signaling network that initiates the earliest synapse pathology in preclinical Alzheimer’s disease brain. *Hum. Mol. Genet.* **24**, 540–558 (2015). 25231903 10.1093/hmg/ddu475

[7] Zarate, C. A. & Manji, H. K. Protein kinase C inhibitors: rationale for use and potential in the treatment of bipolar disorder. *CNS Drugs* **23**, 569–582 (2009). 19552485 10.2165/00023210-200923070-00003 PMC2802274

[8] Geraldes, P. & King, G. L. Activation of protein kinase C isoforms and its impact on diabetic complications. *Circ. Res.* **106**, 1319–1331 (2010). 20431074 10.1161/CIRCRESAHA.110.217117 PMC2877591

[9] Palaniyandi, S. S., Sun, L., Ferreira, J. C. & Mochly-Rosen, D. Protein kinase C in heart failure: a therapeutic target? *Cardiovasc. Res.* **82**, 229–239 (2009). 19168855 10.1093/cvr/cvp001 PMC2675930

[10] Garg, R. et al. Protein kinase C and cancer: what we know and what we do not. *Oncogene* **33**, 5225–5237 (2014). 24336328 10.1038/onc.2013.524 PMC4435965

[11] Sadeghi, M. M., Salama, M. F. & Hannun, Y. A. Protein kinase C as a therapeutic target in non-small cell lung cancer. *Int. J. Mol. Sci.* **22**, 5527 (2021). 10.3390/ijms22115527 PMC8197251 34073823

[12] Wetsel, W. C. et al. Tissue and cellular distribution of the extended family of protein kinase C isoenzymes. *J. Cell Biol.* **117**, 121–133 (1992). 1556149 10.1083/jcb.117.1.121 PMC2289401

[13] Newton, A. C. Protein kinase C: perfectly balanced. *Crit. Rev. Biochem. Mol. Biol.* **53**, 208–230 (2018). 29513138 10.1080/10409238.2018.1442408 PMC5901981

[14] Kedei, N. et al. Characterization of the interaction of ingenol 3-angelate with protein kinase C. *Cancer Res.* **64**, 3243–3255 (2004). 15126366 10.1158/0008-5472.can-03-3403

[15] Steinberg, S. F. Structural basis of protein kinase C isoform function. *Physiol. Rev.* **88**, 1341–1378 (2008). 18923184 10.1152/physrev.00034.2007 PMC2899688

[16] Chalfant, C. E. et al. Regulation of alternative splicing of protein kinase C beta by insulin. *J. Biol. Chem.* **270**, 13326–13332 (1995). 7768933 10.1074/jbc.270.22.13326

[17] Lučić, I., Truebestein, L. & Leonard, T. A. Novel features of DAG-activated PKC isozymes reveal a conserved 3-D architecture. *J. Mol. Biol.* **428**, 121–141 (2016). 26582574 10.1016/j.jmb.2015.11.001

[18] Parissenti, A. M., Kirwan, A. F., Kim, S. A., Colantonio, C. M. & Schimmer, B. P. Inhibitory properties of the regulatory domains of human protein kinase Calpha and mouse protein kinase Cepsilon. *J. Biol. Chem.* **273**, 8940–8945 (1998). 9535877 10.1074/jbc.273.15.8940

[19] Gould, C. M., Kannan, N., Taylor, S. S. & Newton, A. C. The chaperones Hsp90 and Cdc37 mediate the maturation and stabilization of protein kinase C through a conserved PXXP motif in the C-terminal tail. *J. Biol. Chem.* **284**, 4921–4935 (2009). 19091746 10.1074/jbc.M808436200 PMC2643500

[20] Keramisanou, D., Vasantha Kumar, M. V., Boose, N., Abzalimov, R. R. & Gelis, I. Assembly mechanism of early Hsp90-Cdc37-kinase complexes. *Sci. Adv.* **8**, eabm9294 (2022). 35294247 10.1126/sciadv.abm9294 PMC8926337

[21] Feng, X. & Hannun, Y. A. An essential role for autophosphorylation in the dissociation of activated protein kinase C from the plasma membrane. *J. Biol. Chem.* **273**, 26870–26874 (1998). 9756933 10.1074/jbc.273.41.26870

[22] Pappa, H., Dekker, L. V., Parker, P. J. & McDonald, N. Q. Preliminary X-ray analysis of a C2-like domain from protein kinase C-delta. *Acta Crystallogr. D Biol. Crystallogr.* **54**, 693–696 (1998). 9761878 10.1107/s0907444997019732

[23] Hommel, U., Zurini, M. & Luyten, M. Solution structure of a cysteine rich domain of rat protein kinase C. *Nat. Struct. Biol.* **1**, 383–387 (1994). 7664052 10.1038/nsb0694-383

[24] Zhang, G., Kazanietz, M. G., Blumberg, P. M. & Hurley, J. H. Crystal structure of the cys2 activator-binding domain of protein kinase C delta in complex with phorbol ester. *Cell* **81**, 917–924 (1995). 7781068 10.1016/0092-8674(95)90011-x

[25] Xu, Z. B. et al. Catalytic domain crystal structure of protein kinase C-theta (PKCtheta). *J. Biol. Chem.* **279**, 50401–50409 (2004). 15364937 10.1074/jbc.M409216200

[26] Grodsky, N. et al. Structure of the catalytic domain of human protein kinase C beta II complexed with a bisindolylmaleimide inhibitor. *Biochemistry* **45**, 13970–13981 (2006). 17115692 10.1021/bi061128h

[27] Sutton, R. B. & Sprang, S. R. Structure of the protein kinase Cbeta phospholipid-binding C2 domain complexed with Ca2+. *Structure* **6**, 1395–1405 (1998). 9817842 10.1016/s0969-2126(98)00139-7

[28] Violin, J. D., Zhang, J., Tsien, R. Y. & Newton, A. C. A genetically encoded fluorescent reporter reveals oscillatory phosphorylation by protein kinase C. *J. Cell Biol.* **161**, 899–909 (2003). 12782683 10.1083/jcb.200302125 PMC2172956

[29] Leonard, T. A., Rozycki, B., Saidi, L. F., Hummer, G. & Hurley, J. H. Crystal structure and allosteric activation of protein kinase C βII. *Cell* **144**, 55–66 (2011). 21215369 10.1016/j.cell.2010.12.013 PMC3104240

[30] Antal, C. E., Callender, J. A., Kornev, A. P., Taylor, S. S. & Newton, A. C. Intramolecular C2 domain-mediated autoinhibition of protein kinase C βII. *Cell Rep.* **12**, 1252–1260 (2015). 26279568 10.1016/j.celrep.2015.07.039 PMC4551583

[31] Mina, L. et al. A phase II study of oral enzastaurin in patients with metastatic breast cancer previously treated with an anthracycline and a taxane containing regimen. *Invest. New Drugs* **27**, 565–570 (2009). 19214387 10.1007/s10637-009-9220-1

[32] Clemons, M. et al. Phase II, double-blind, randomized trial of capecitabine plus enzastaurin versus capecitabine plus placebo in patients with metastatic or recurrent breast cancer after prior anthracycline and taxane therapy. *Breast Cancer Res. Treat.* **124**, 177–186 (2010). 20814815 10.1007/s10549-010-1152-0

[33] Millward, M. J. et al. The multikinase inhibitor midostaurin (PKC412A) lacks activity in metastatic melanoma: a phase IIA clinical and biologic study. *Br. J. Cancer* **95**, 829–834 (2006). 16969355 10.1038/sj.bjc.6603331 PMC2360547

[34] Robertson, M. J. et al. Phase II study of enzastaurin, a protein kinase C beta inhibitor, in patients with relapsed or refractory diffuse large B-cell lymphoma. *J Clin. Oncol.* **25**, 1741–1746 (2007). 17389337 10.1200/JCO.2006.09.3146

[35] Macedo, L. F., Sabnis, G. J., Goloubeva, O. G. & Brodie, A. Combination of anastrozole with fulvestrant in the intratumoral aromatase xenograft model. *Cancer Res.* **68**, 3516–3522 (2008). 18451180 10.1158/0008-5472.CAN-07-6807

[36] Kreisl, T. N. et al. A phase I/II trial of enzastaurin in patients with recurrent high-grade gliomas. *Neuro. Oncol.* **12**, 181–189 (2010). 20150385 10.1093/neuonc/nop042 PMC2940576

[37] Mochly-Rosen, D., Das, K. & Grimes, K. V. Protein kinase C, an elusive therapeutic target? *Nat. Rev. Drug Discov.* **11**, 937–957 (2012). 23197040 10.1038/nrd3871 PMC3760692

[38] Arencibia, J. M. et al. An allosteric inhibitor scaffold targeting the PIF-pocket of atypical protein kinase C isoforms. *ACS Chem. Biol.* **12**, 564–573 (2017). 28045490 10.1021/acschembio.6b00827

[39] Mansfield, A. S. et al. Phase I dose escalation study of the PKCι inhibitor aurothiomalate for advanced non-small-cell lung cancer, ovarian cancer, and pancreatic cancer. *Anticancer Drugs* **24**, 1079–1083 (2013). 23962904 10.1097/CAD.0000000000000009 PMC3937851

[40] Yin, N. et al. Protein kinase Cι mediates immunosuppression in lung adenocarcinoma. *Sci. Transl. Med.* **14**, eabq5931 (2022). 36383684 10.1126/scitranslmed.abq5931 PMC11457891

[41] Jayaraman, S. et al. Endoxifen downregulates AKT phosphorylation through protein kinase C beta 1 inhibition in ERalpha+ breast cancer. *NPJ Breast Cancer* **9**, 101 (2023). 38114522 10.1038/s41523-023-00606-2 PMC10730845

[42] Mukai, H. & Ono, Y. Expression and purification of protein kinase C from insect cells. *Methods Mol. Biol.* **233**, 21–34 (2003). 12840495 10.1385/1-59259-397-6:21

[43] Baffi, T. R., Van, A. N., Zhao, W., Mills, G. B. & Newton, A. C. Protein kinase C quality control by phosphatase PHLPP1 unveils loss-of-function mechanism in cancer. *Mol. Cell* **74**, 378–392 e375 (2019). 30904392 10.1016/j.molcel.2019.02.018 PMC6504549

[44] Cong, A. T. Q., Witter, T. L. & Schellenberg, M. J. High-efficiency recombinant protein purification using mCherry and YFP nanobody affinity matrices. *Protein Sci.* **31**, e4383 (2022). 36040252 10.1002/pro.4383 PMC9413470

[45] Cong, A. T. Q. & Schellenberg, M. J. Recombinant topoisomerase 2 production using cultured human cell lines. *Methods Mol. Biol.* **2928**, 207–222 (2025). 40372648 10.1007/978-1-0716-4550-5_17

[46] Keranen, L. M., Dutil, E. M. & Newton, A. C. Protein kinase C is regulated in vivo by three functionally distinct phosphorylations. *Curr. Biol.* **5**, 1394–1403 (1995). 8749392 10.1016/s0960-9822(95)00277-6

[47] Antal, C. orinaE., Violin, J. onathanD., Kunkel, M. ayaT., Skovsø, S. & Newton, A. lexandraC. Intramolecular conformational changes optimize protein kinase C signaling. *Chem. Biol.* **21**, 459–469 (2014). 24631122 10.1016/j.chembiol.2014.02.008 PMC4020788

[48] Nalefski, E. A. & Newton, A. C. Membrane binding kinetics of protein kinase C betaII mediated by the C2 domain. *Biochemistry* **40**, 13216–13229 (2001). 11683630 10.1021/bi010761u

[49] Oancea, E. & Meyer, T. Protein kinase C as a molecular machine for decoding calcium and diacylglycerol signals. *Cell* **95**, 307–318 (1998). 9814702 10.1016/s0092-8674(00)81763-8

[50] Nishikawa, K., Toker, A., Johannes, F. J., Songyang, Z. & Cantley, L. C. Determination of the specific substrate sequence motifs of protein kinase C isozymes. *J. Biol. Chem.* **272**, 952–960 (1997). 8995387 10.1074/jbc.272.2.952

[51] Patel, N. A. et al. Insulin regulates protein kinase CbetaII alternative splicing in multiple target tissues: development of a hormonally responsive heterologous minigene. *Mol. Endocrinol.* **18**, 899–911 (2004). 14752056 10.1210/me.2003-0391

[52] Arter, C., Trask, L., Ward, S., Yeoh, S. & Bayliss, R. Structural features of the protein kinase domain and targeted binding by small-molecule inhibitors. *J. Biol. Chem.* **298**, 102247 (2022). 35830914 10.1016/j.jbc.2022.102247 PMC9382423

[53] Welsh, C. L., Conklin, A. E. & Madan, L. K. Crystal structures reveal hidden domain mechanics in protein kinase A (PKA). *Biology (Basel)* **12**, 1370 (2023). 10.3390/biology12111370 PMC10669547 37997969

[54] Jumper, J. et al. Highly accurate protein structure prediction with AlphaFold. *Nature* **596**, 583–589 (2021). 34265844 10.1038/s41586-021-03819-2 PMC8371605

[55] Gundimeda, U., Chen, Z. H. & Gopalakrishna, R. Tamoxifen modulates protein kinase C via oxidative stress in estrogen receptor-negative breast cancer cells. *J. Biol. Chem.* **271**, 13504–13514 (1996). 8662863 10.1074/jbc.271.23.13504

[56] Ali, S. M. et al. Endoxifen is a new potent inhibitor of PKC: a potential therapeutic agent for bipolar disorder. *Bioorg. Med. Chem. Lett.* **20**, 2665–2667 (2010). 20227879 10.1016/j.bmcl.2010.02.024

[57] Goetz, M. P. et al. First-in-human phase I study of the tamoxifen metabolite Z-endoxifen in women with endocrine-refractory metastatic breast cancer. *J. Clin. Oncol.* **35**, 3391–3400 (2017). 28854070 10.1200/JCO.2017.73.3246 PMC5648176

[58] Faul, M. M. et al. Acyclic N-(azacycloalkyl)bisindolylmaleimides: isozyme selective inhibitors of PKCbeta. *Bioorg. Med. Chem. Lett.* **13**, 1857–1859 (2003). 12749884 10.1016/s0960-894x(03)00286-5

[59] Cimmperman, P. et al. A quantitative model of thermal stabilization and destabilization of proteins by ligands. *Biophys. J.* **95**, 3222–3231 (2008). 18599640 10.1529/biophysj.108.134973 PMC2547457

[60] Leitner, P. D., Vietor, I., Huber, L. A. & Valovka, T. Fluorescent thermal shift-based method for detection of NF-κB binding to double-stranded DNA. *Sci. Rep.* **11**, 2331 (2021). 33504856 10.1038/s41598-021-81743-1 PMC7840993

[61] Yue, W., Zhou, D., Chen, S. & Brodie, A. A new nude mouse model for postmenopausal breast cancer using MCF-7 cells transfected with the human aromatase gene. *Cancer Res.* **54**, 5092–5095 (1994). 7923123

[62] Long, B. J. et al. Therapeutic strategies using the aromatase inhibitor letrozole and tamoxifen in a breast cancer model. *J. Natl. Cancer Inst.* **96**, 456–465 (2004). 15026471 10.1093/jnci/djh076

[63] Sierecki, E., Sinko, W., McCammon, J. A. & Newton, A. C. Discovery of small molecule inhibitors of the PH domain leucine-rich repeat protein phosphatase (PHLPP) by chemical and virtual screening. *J. Med. Chem.* **53**, 6899–6911 (2010). 20836557 10.1021/jm100331d PMC2951065

[64] Nagao, M. et al. Role of protein phosphatases in malignant transformation. *Princess Takamatsu Symp.* **20**, 177–184 (1989). 2562181

[65] Inoue, M., Kishimoto, A., Takai, Y. & Nishizuka, Y. Studies on a cyclic nucleotide-independent protein kinase and its proenzyme in mammalian tissues. II. Proenzyme and its activation by calcium-dependent protease from rat brain. *J. Biol. Chem.* **252**, 7610–7616 (1977). 199594

[66] Das, J., Ramani, R. & Suraju, M. O. Polyphenol compounds and PKC signaling. *Biochim. Biophys. Acta* **1860**, 2107–2121 (2016). 27369735 10.1016/j.bbagen.2016.06.022 PMC4961512

[67] Yin, N., Liu, Y., Murray, N. R. & Fields, A. P. Oncogenic protein kinase Cι signaling mechanisms in lung cancer: implications for improved therapeutic strategies. *Adv. Biol. Regul.* **75**, 100656 (2020). 31623973 10.1016/j.jbior.2019.100656

[68] Husremović, T. et al. PHLPP2 is a pseudophosphatase that lost activity in the metazoan ancestor. *Proc. Natl. Acad. Sci. USA*. **122**, e2417218122 (2025). 40168118 10.1073/pnas.2417218122 PMC12002173

[69] Jayaraman, S. et al. Antitumor activity of Z-endoxifen in aromatase inhibitor-sensitive and aromatase inhibitor-resistant estrogen receptor-positive breast cancer. *Breast Cancer Res*. **22**, 51 (2020). 32430040 10.1186/s13058-020-01286-7 PMC7238733

[70] Takebe, N. et al. Phase 1 study of Z-endoxifen in patients with advanced gynecologic, desmoid, and hormone receptor-positive solid tumors. *Oncotarget* **12**, 268–277 (2021). 33659039 10.18632/oncotarget.27887 PMC7899551

[71] Saxena, A. et al. Role of rotein kinase C in bipolar disorder: a review of the current literature. *Mol. Neuropsychiatry* **3**, 108–124 (2017). 29230399 10.1159/000480349 PMC5701269

[72] Ahmad, A. et al. Endoxifen: a new, protein kinase C inhibitor to treat acute and mixed mania associated with bipolar I disorder. *Bipolar Disord.* **23**, 595–603 (2021). 33368969 10.1111/bdi.13041

[73] Schellenberg, M. J., Petrovich, R. M., Malone, C. C. & Williams, R. S. Selectable high-yield recombinant protein production in human cells using a GFP/YFP nanobody affinity support. *Protein Sci.* **27**, 1083–1092 (2018). 29577475 10.1002/pro.3409 PMC5980532

[74] Otwinowski, Z. & Minor, W. Processing of X-ray diffraction data collected in oscillation mode. *Methods Enzymol.* **276**, 307–326 (1997). 27754618 10.1016/S0076-6879(97)76066-X

[75] McCoy, A. J. et al. Phaser crystallographic software. *J. Appl. Crystallogr.* **40**, 658–674 (2007). 19461840 10.1107/S0021889807021206 PMC2483472

[76] Emsley, P., Lohkamp, B., Scott, W. G. & Cowtan, K. Features and development of Coot. *Acta Crystallogr. D Biol. Crystallogr.* **66**, 486–501 (2010). 20383002 10.1107/S0907444910007493 PMC2852313

[77] Adams, P. D. et al. PHENIX: a comprehensive Python-based system for macromolecular structure solution. *Acta Crystallogr. D Biol. Crystallogr.* **66**, 213–221 (2010). 20124702 10.1107/S0907444909052925 PMC2815670

[78] Miyawaki, A. & Tsien, R. Y. Monitoring protein conformations and interactions by fluorescence resonance energy transfer between mutants of green fluorescent protein. *Methods Enzymol.* **327**, 472–500 (2000). 11045004 10.1016/s0076-6879(00)27297-2

[79] Manalastas-Cantos, K. et al. ATSAS 3.0: expanded functionality and new tools for small-angle scattering data analysis. *J. Appl. Crystallogr.* **54**, 343–355 (2021). 33833657 10.1107/S1600576720013412 PMC7941305

