# Supplementary material for: Molecular basis of allosteric regulation and pharmaceutical targeting of protein kinase Cβ
Source: Nat Commun. 2026 May 21;17:6703. doi: 10.1038/s41467-026-73413-5 (PMC13385876; doi:10.1038/s41467-026-73413-5)
Supplement: Supplementary file 1 — Supplementary Information [file 41467_2026_73413_MOESM1_ESM.pdf]

Supplementary Materials for

Molecular Basis of Allosteric Regulation and Pharmaceutical Targeting of Protein Kinase Cβ

Anh T.Q. Cong, Taylor L. Witter, Elizabeth S. Bruinsma, Sayantani Sarkar Bhattacharya, Swaathi Jayaraman, Samuel R. Wyatt, Jasper K. Solverson, Maria B. Dugan, Jasmina Paluncic, Mary J. Kuffel, Julia R. Alvey, Huy V. Huynh, Xinyan Wu, Alan P. Fields, Akhilesh Pandey John R. Hawse, Matthew P. Goetz, and Matthew J. Schellenberg
Corresponding author: schellenberg.matthew@mayo.edu and goetz.matthew@mayo.edu

The PDF file includes:

Supplementary Figures 1 to 10
Supplementary Tables 1 to 3

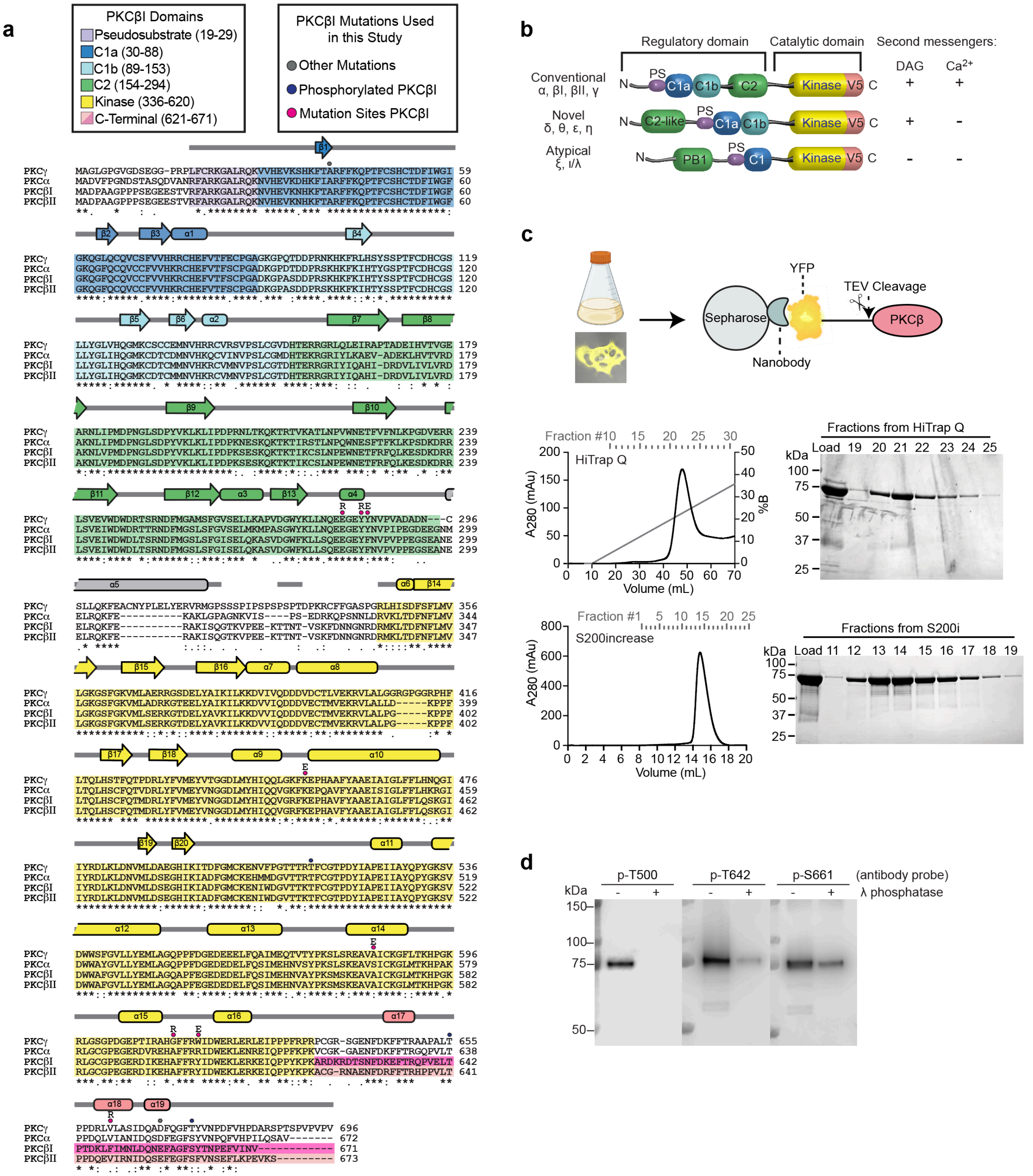

Supplementary Figure 1 (**a**) Sequence alignment of canonical PKCs with domains coloured as indicated. The altered C-terminal tails derived from an alternative splicing event are coloured pink (PKCβI) or salmon (PKCβII). (**b**) Domain arrangement and second messenger sensitivity of the three PKC families. (**c**) Schematic diagram of YFP-PKCβII protein expression and affinity isolation using the anti-YFP nanobody system. Chromatograms and corresponding Coomassie-stained gels of PKCβII from FPLC purification steps. Created in BioRender. Cong, A. (2026) https://BioRender.com/3jamc8o. (**d**) Western blots with the indicated phospho-specific PKCβI antibodies detects phosphorylation at all three expected sites (T500 – activation, T642 – turn, and S661 – hydrophobic) in the purified protein. The blots were performed once. Source data are provided as a Source Data file.

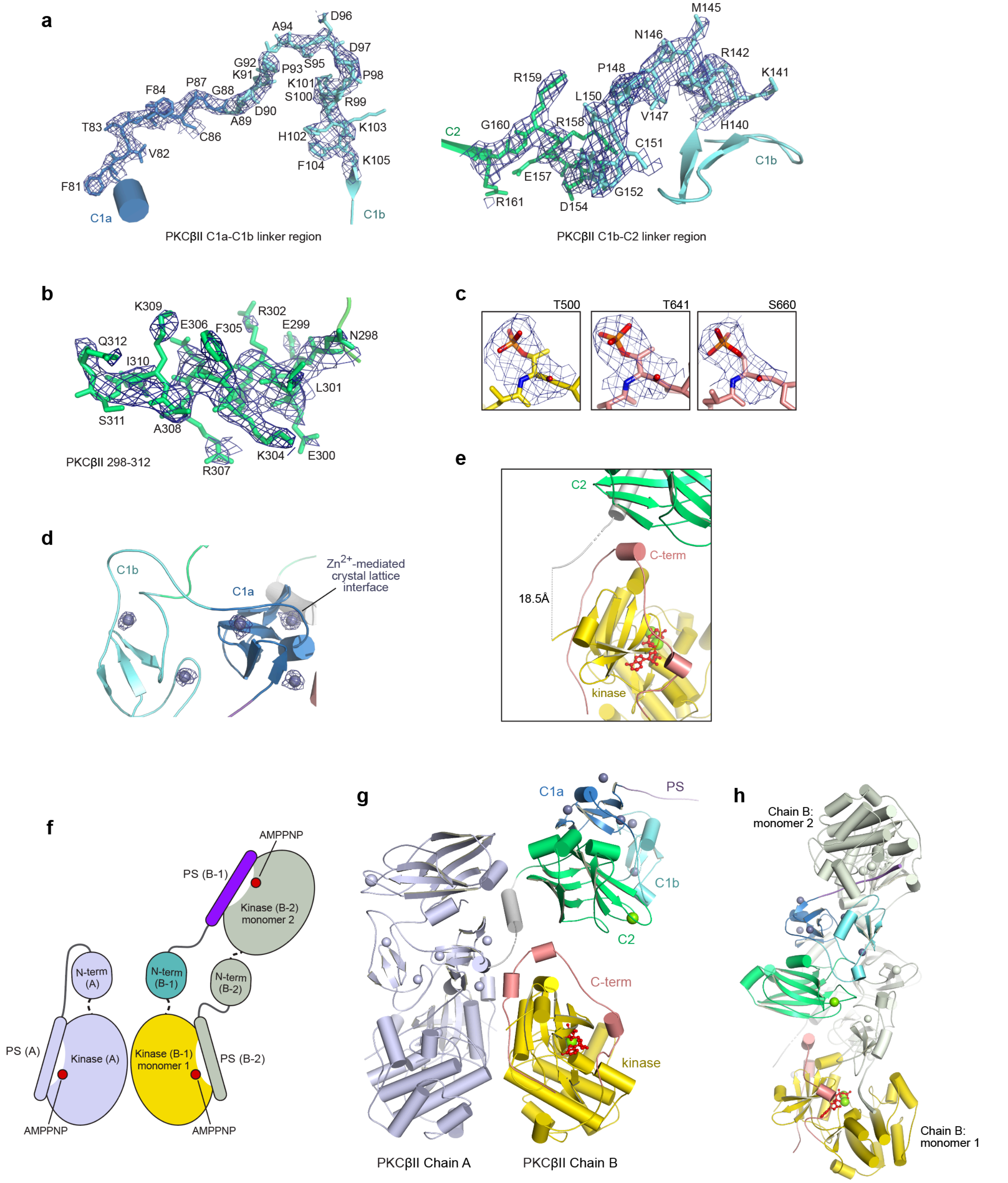

Supplementary Figure 2 (**a**) $2mF_0 - dF_c$ map contoured at 1σ indicating continuous electron density for the PKCβII C1a – C1b linker region and C1b – C2 linker region. (**b**) $2mF_0 - dF_c$ map contoured at 1σ for residues 298 – 312 in the PKCβII C2-kinase domain linker region. (**c**) $2mF_0 - dF_c$ contoured at 1σ of the three phosphorylation sites on PKCβII. (**d**) $2mF_0 - dF_c$ map contoured at 4σ shows high electron density of Zn^{2+} ions (grey spheres), which are coordinated within the C1a and C1b domains, as well as a fifth Zn^{2+} ion that mediates a crystal lattice contact. (**e**) Measured length of the disordered region between C2 and kinase domain used to define pairing of N-terminal and kinase domains that belong to the same chain. (**f**) Cartoon demonstration of the asymmetric unit and monomer domain swap in PKCBII crystal with simplified domains in chain

A labelled with (A), chain B monomer 1 labelled with (B-1) and chain B monomer 2 labelled with (B-2) (**g**) Monomer arrangement within the asymmetric unit of PKCBII crystal with chain B coloured as in Fig. 1a and chain B coloured in light purple. (**h**) Domain–swapped arrangement of PKCBII chain B (monomer 1, coloured as in Fig. 1a) with an adjacent identical monomer (monomer 2, grey).

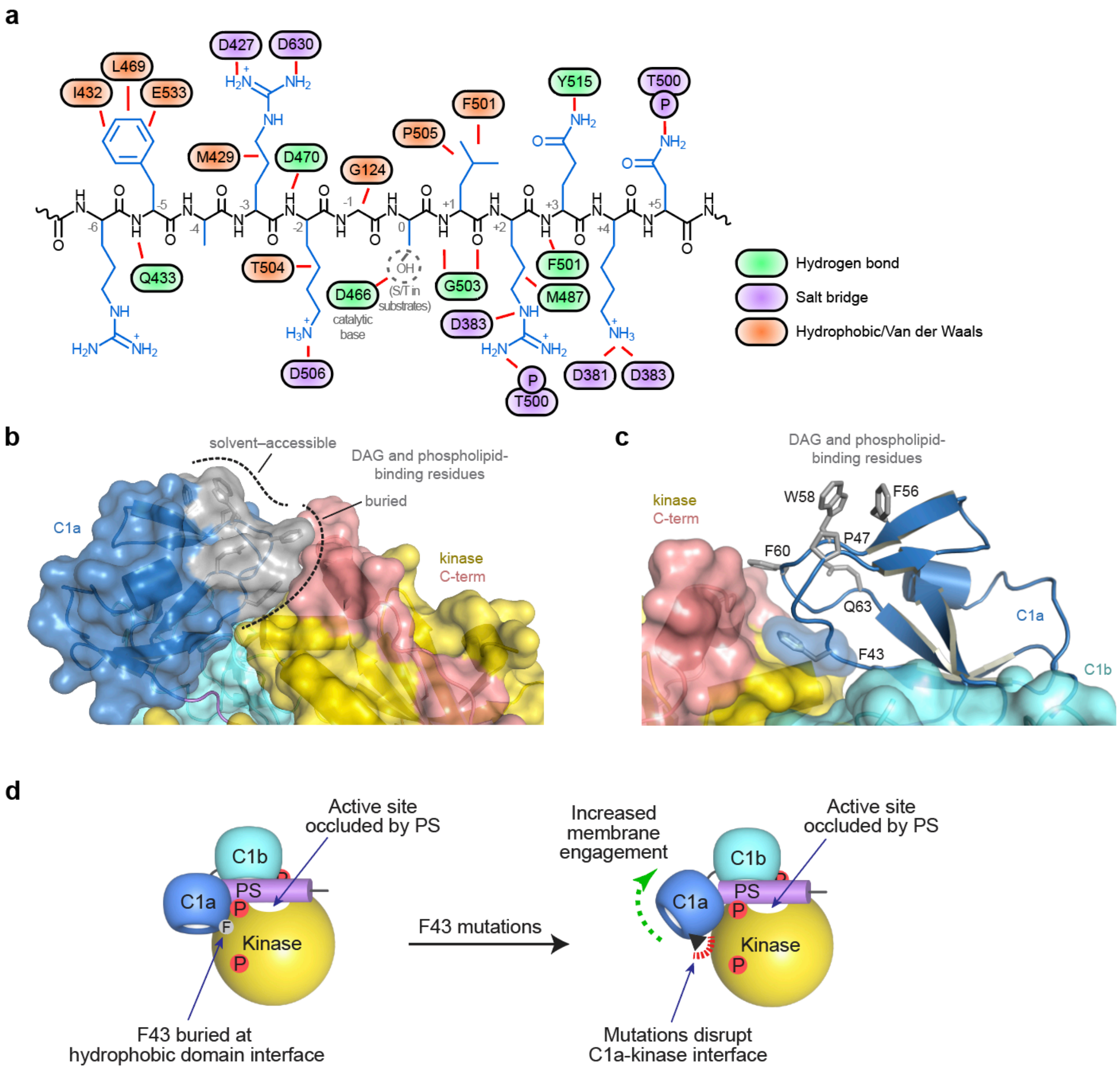

Supplementary Figure 3 (**a**) Molecular details of the N-terminal pseudosubstrate side chains (blue) and their specific interactions with the C1b and kinase domains labelled by residues and color-coded (hydrogen bonding, salt bridge, hydrophobic/Van der Waals). (**b**) Surface representation and (**c**) individual residues of a small portion of the membrane-binding surface is buried between the C1a and kinase interface and is not compatible with membrane-binding. Protein domains coloured as in Fig. 1a. (**d**) Cartoon illustration of how F43 mutations disrupt the C1a-kinase interface to increase phospholipid binding at the C1a domain and kinase hyperactivity.

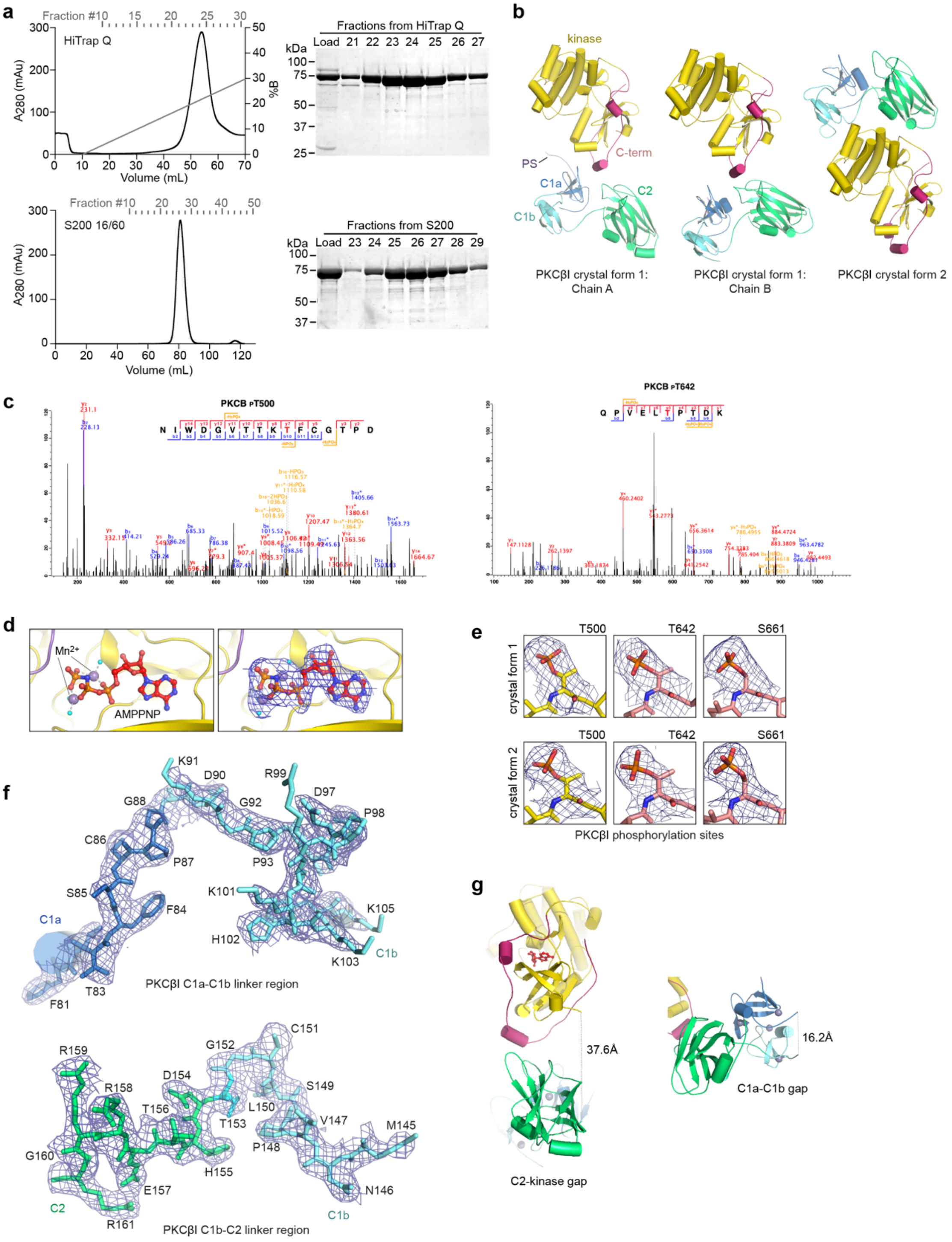

Supplementary Figure 4 (**a**) Ion exchange and size exclusion chromatograms of PKCβI purification and SDS-PAGE analysis of indicated fractions. (**b**) Individual copies of PKCβI within each crystal form, coloured as in Supplementary Figure 1a. (**c**) Mass spectrometry of purified PKCβI protein detects phosphorylation at T500 and T642. (**d**) Experimental $2mF_0 - dF_c$ map of PKCβI contoured at 1σ for the nucleotide AMPPNP with coordinating Mn^{2+} ions (purple) and water (cyan). (**e**) Experimental $2mF_0 - dF_c$ map contoured at 1σ of the phosphorylation sites on PKCβI in each crystal form. (**f**) Experimental $2mF_0 - dF_c$ map contoured at 1σ for the PKCβI C1a – C1b linker region (top panel) and C1b – C2 linker region (bottom panel). (**g**) Disordered linkers connecting the C1a-C1b domain gap and the C2-kinase domain gap in PKCβI crystal form 1. Source data are provided as a Source Data file.

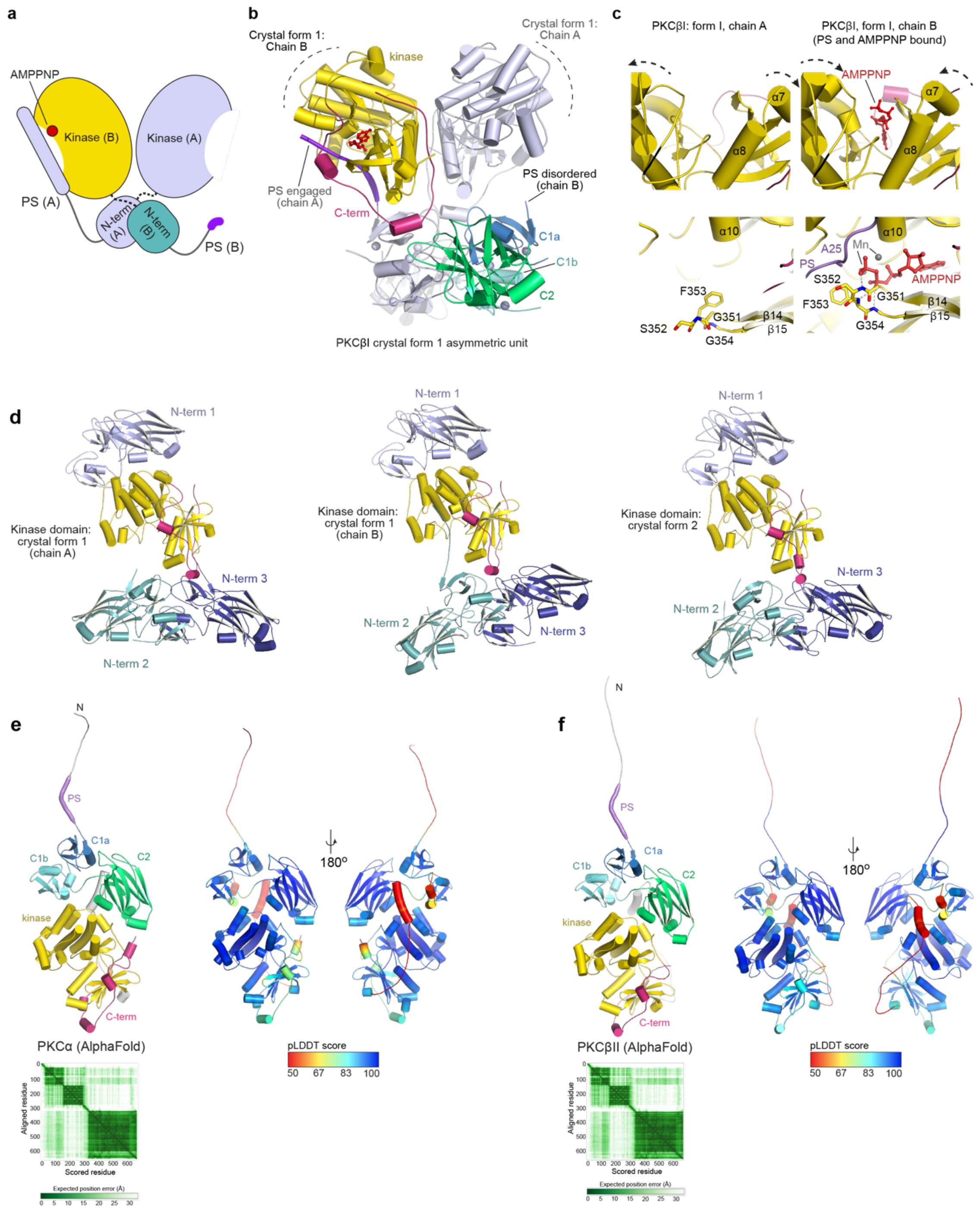

Supplementary Figure 5 (**a**) Cartoon demonstration of the asymmetric unit and monomer domain swap in PKCBI crystal form 1 with simplified domains in chain A labelled with (A), chain B labelled with (B). (**b**) The asymmetric unit of crystal form 1 shows a domain-swap interaction between two PKCBI monomers with the PS of the adjacent monomer (chain A, light purple) is bound to the active site of chain B (coloured as in Fig. 1A). (**c**) PKCβI active site adopts a more closed conformation when occupied by AMPPNP nucleotide and remains open in its absence. (**d**) Local symmetry environment of the three copies of PKCβI reveals similar molecular interactions. (**e**) AlphaFold-predicted structure of PKCα and (**f**) PKCβII and accompanied PAE plots show a domain arrangement that is similar to the active form. Domains coloured as in Supplementary Figure 1a.

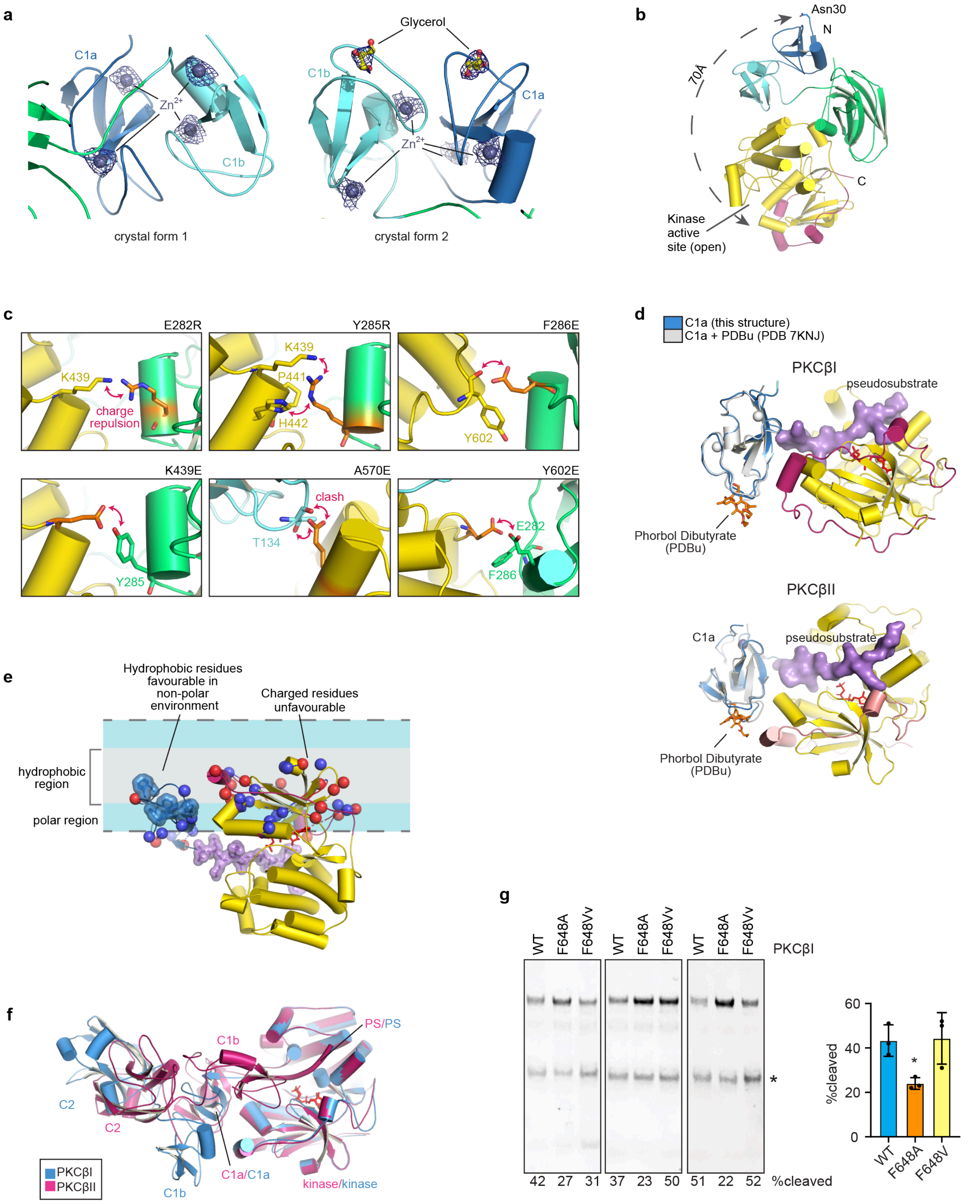

Supplementary Figure 6 (**a**) Ligands coordinate with their experimental 2mF$_0$ – dF$_c$ maps contoured at 4σ (Zn^{2+}) and 1σ (glycerol) in crystal form 1 and crystal form 2 of PKCβI. DAG binding site of PKCβI is occupied by glycerol molecules in crystal form 2. (**b**) 70 Å distance between N-terminal tail and active site prevents re-engagement of the pseudosubstrate. (**c**) Modelled mutations that disrupt C2-kinase and C1b-kinase domain interactions in the active conformation of PKCβI. (**d**) Aligned crystal structures of the C1a domain with PDBu (PDB 7KNJ)

and PKCβI or PKCβII in their auto-inhibited conformation shows PDBu binding does not disrupt the inactive conformation. (**e**) Insertion of C1a domain hydrophobic residues into the hydrophobic region of phospholipid bilayer would cause a steric clash between the kinase domain and phospholipid bilayer, which would result in separation of the kinase domain from the pseudosubstrate. (**f**) Structural alignment of the auto-inhibited conformation of PKCβI and PKCβII. (**g**) Limited proteolysis of PKCβI WT and the two mutants F648A and F648V. Cleavage efficiency is calculated from band intensity and shown as % cleaved. mean ± s.d., P-values were determined using a two-tailed t-test with n=3 independent experiments. Source data are provided as a Source Data file.

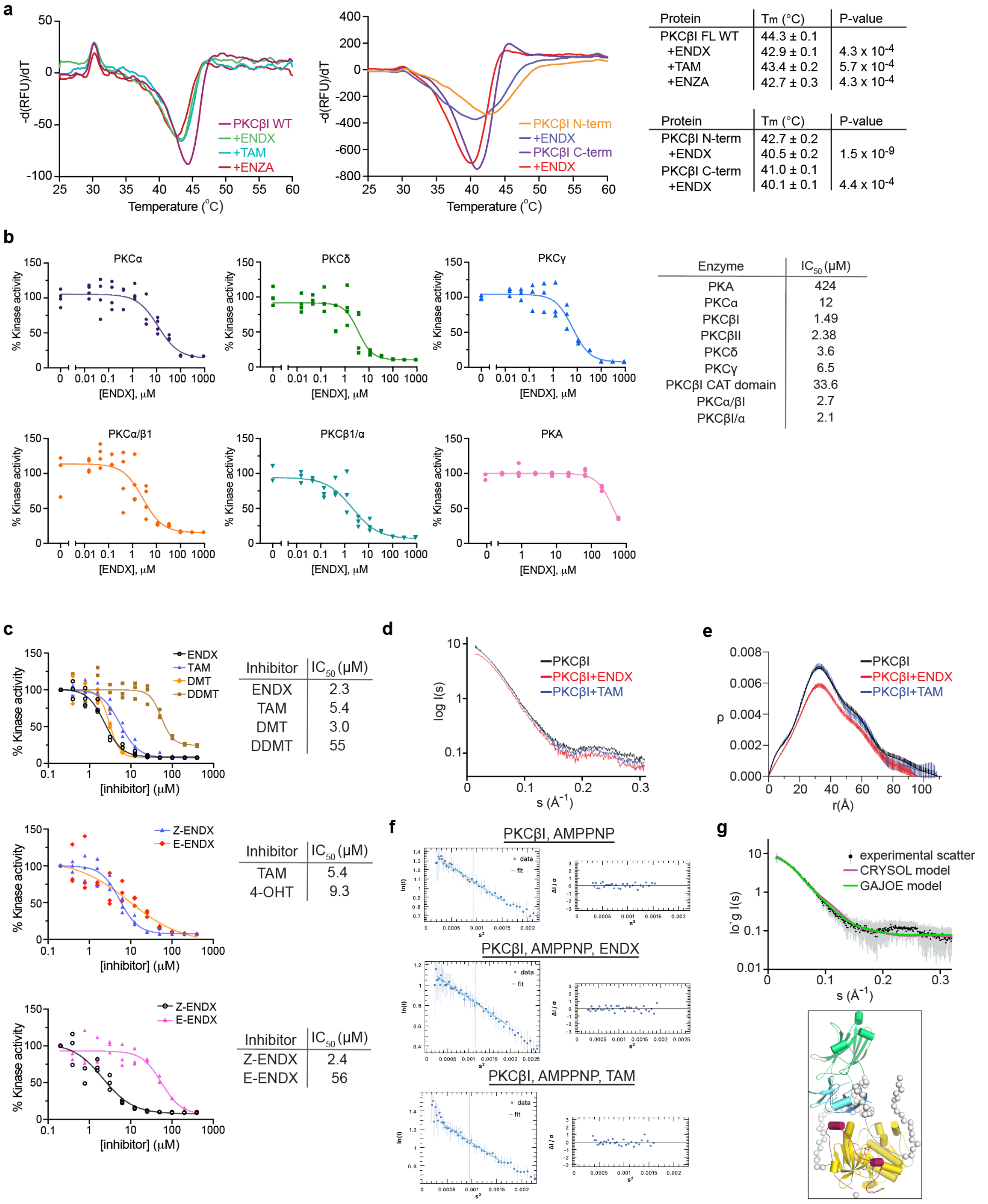

Supplementary Figure 7 (**a**) DSF measurements of PKCβI constructs in the presence of indicated inhibitors. Data are mean ± SD and P-values reported are from a two-tailed t-test with n=4 (PKCβI FL WT and C-term) and n = 9 (PKCβI N-term) biological replicates. (**b**) *In vitro* Z'-LYTE kinase assay of other PKC isoforms and their associated IC_{50} values. n=3 technical replicates for PKCγ, PKCβ1/α, and PKA. n=4 technical replicates for PKCα, PKCδ, and PKCα/β1. Values for PKCβI and PKCβII are reproduced from Fig. 5c. (**c**) *In vitro* Z'-LYTE kinase assay of PKCβI in the presence of the indicated inhibitors (DMT = N-desmethyltamoxifen, DDMT = N,N-

didesmethyltamoxifen, 4-OHT = 4-hydroxytamoxifen) and their associated IC_{50} values. (n=3 technical replicates). (**d**) SAXS scattering plots of PKCβI in the presence of the indicated ligands. (**e**) Electron pair distribution (ρ(r)) plots calculated from SAXS data for PKCβI in the presence of the indicated ligands. (**f**) (left) Guinier plots and (right) residuals calculated from SAXS data shown in Fig. 5d. (**g**) (upper) Comparison of observed scattering with theoretical scattering generated from the model of PKCβI in the inactive conformation generated by CRYSOL or that from EOM used to model the unobserved residues. Black dots indicate mean pixel intensity, grey error bars, s.d. (lower) Model of PKCβI in the inactive conformation with domains colored as in Supplementary Figure 1a, with dummy residues modelled by EOM as grey spheres. Source data are provided as a Source Data file.

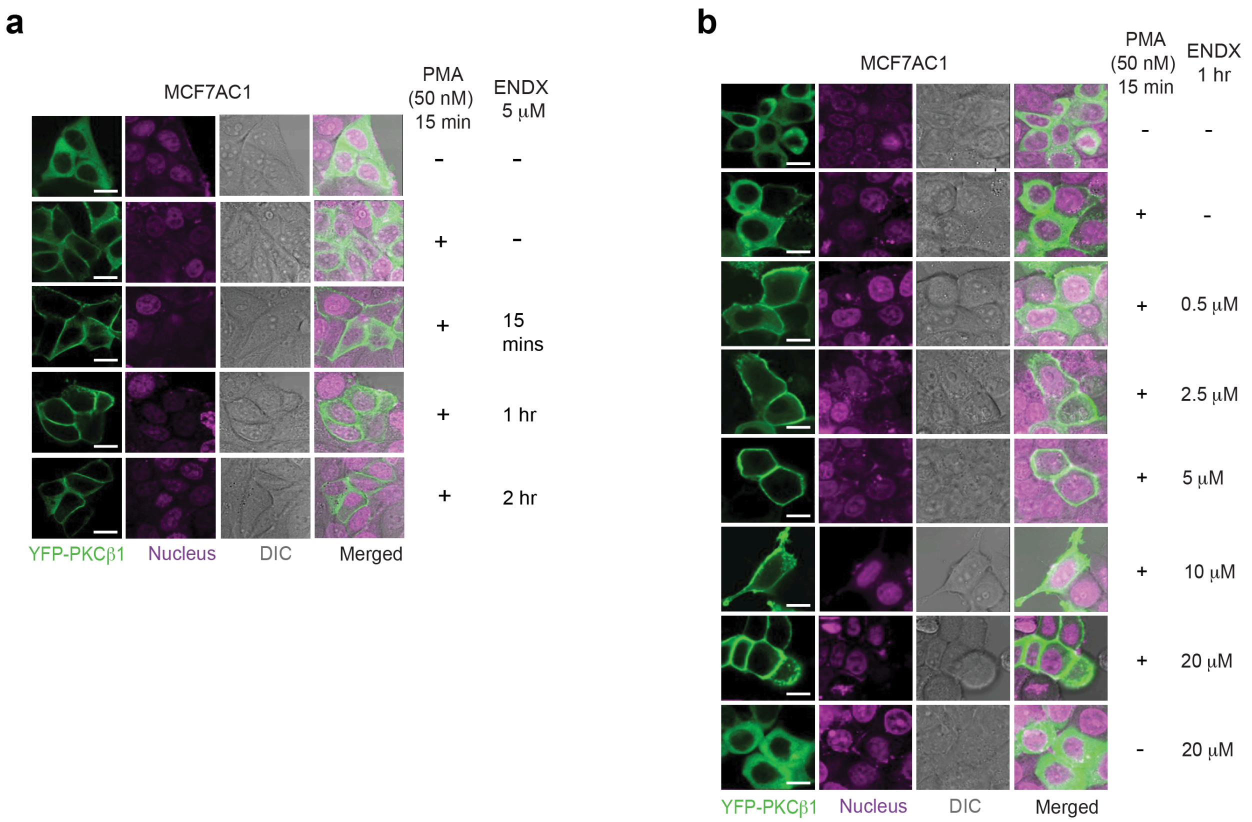

Supplementary Figure 8 (**a**) Confocal live cell microscopy images showing time course of YFP-tagged PKCβI accumulation at the plasma membrane in the presence of indicated compounds. Scale bar = 20 μm. (**b**) Confocal live cell microscopy images showing concentration dependence of YFP-tagged PKCβI accumulation at the plasma membrane in the presence of ENDX. Scale bar = 20 μm.

a 50 nM PMA

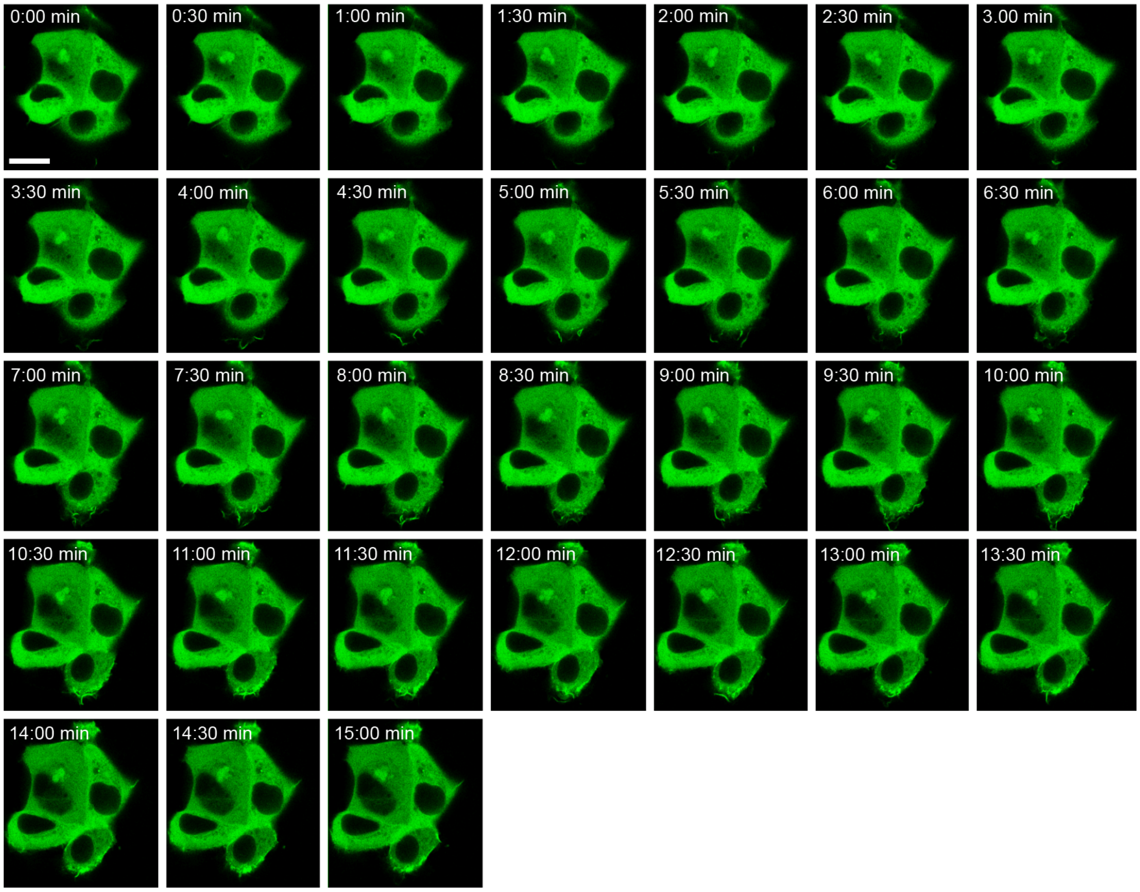

b 50 nM PMA with 5 μM ENDX pretreatment (1h)

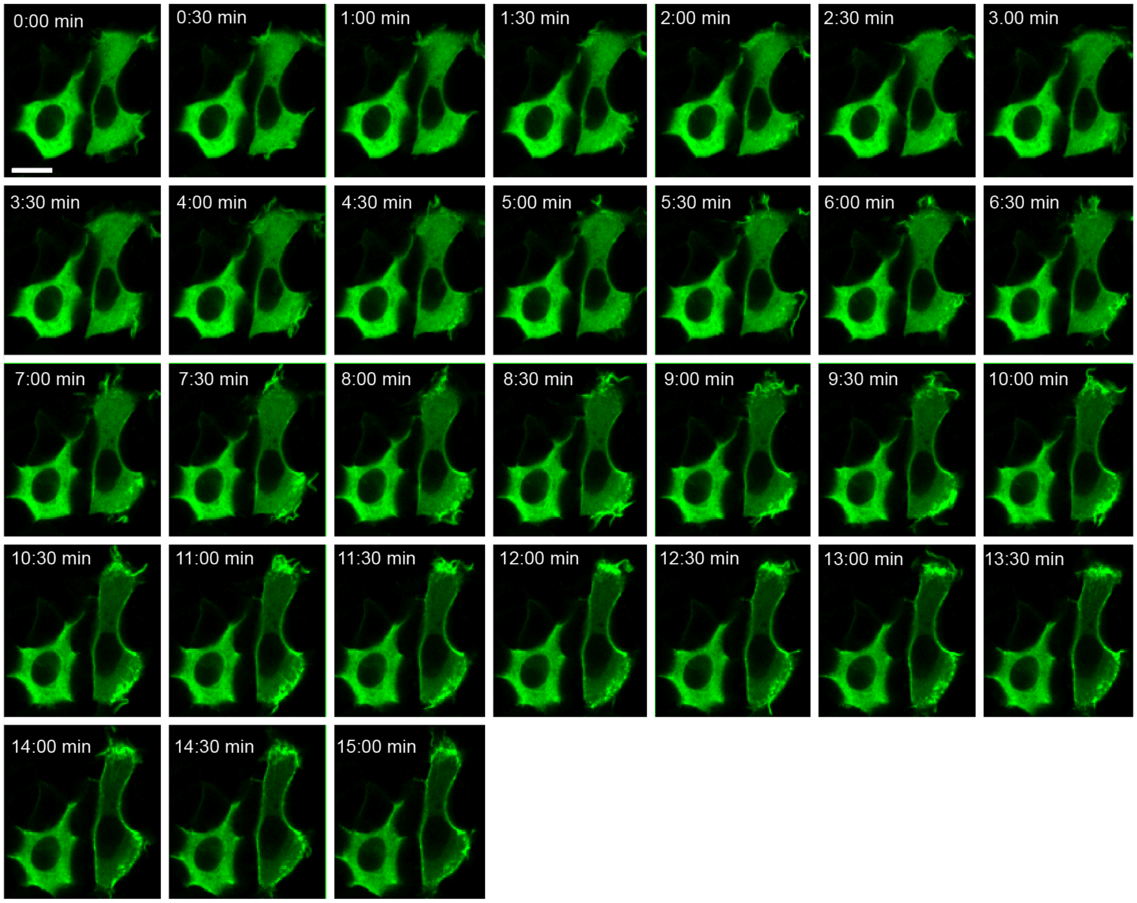

c 50 nM PMA

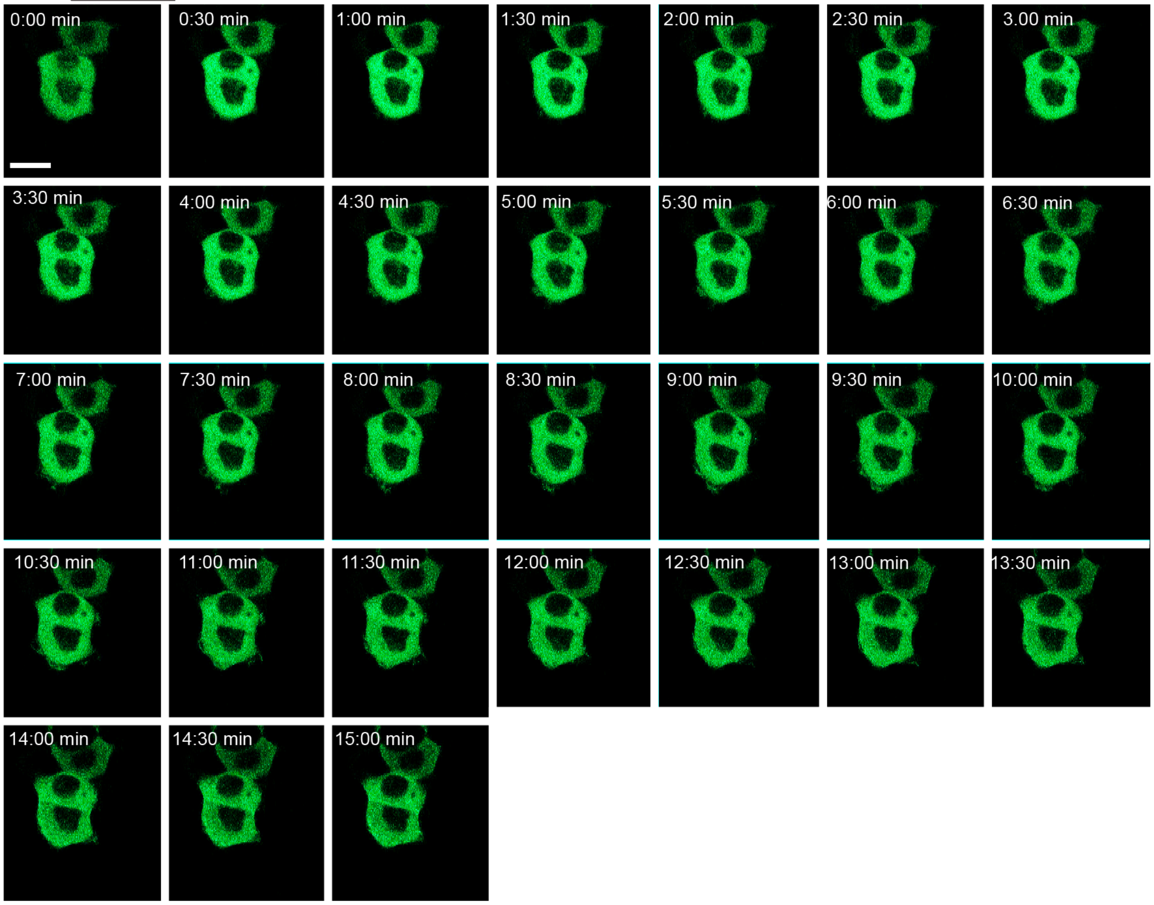

d 50 nM PMA with 5 μM ENDX pretreatment (1h)

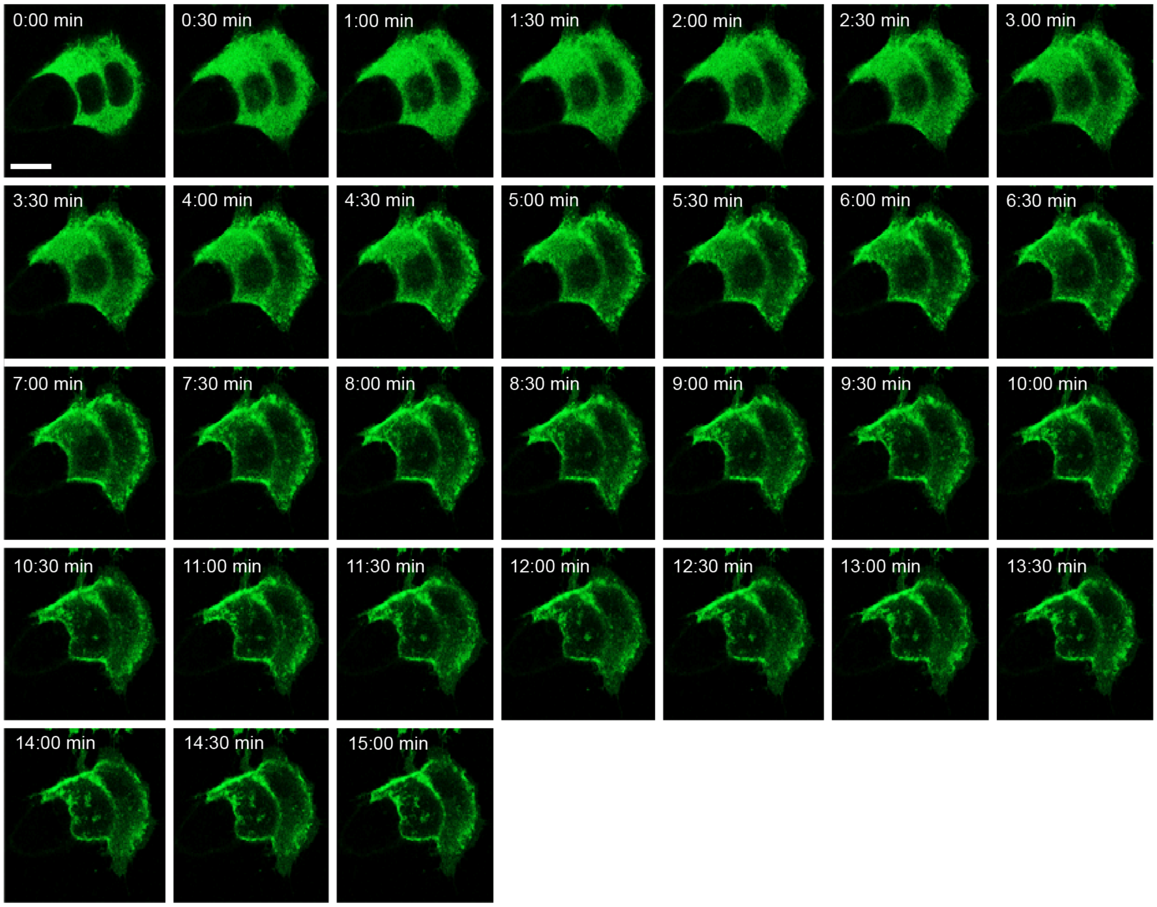

e 50 nM PMA

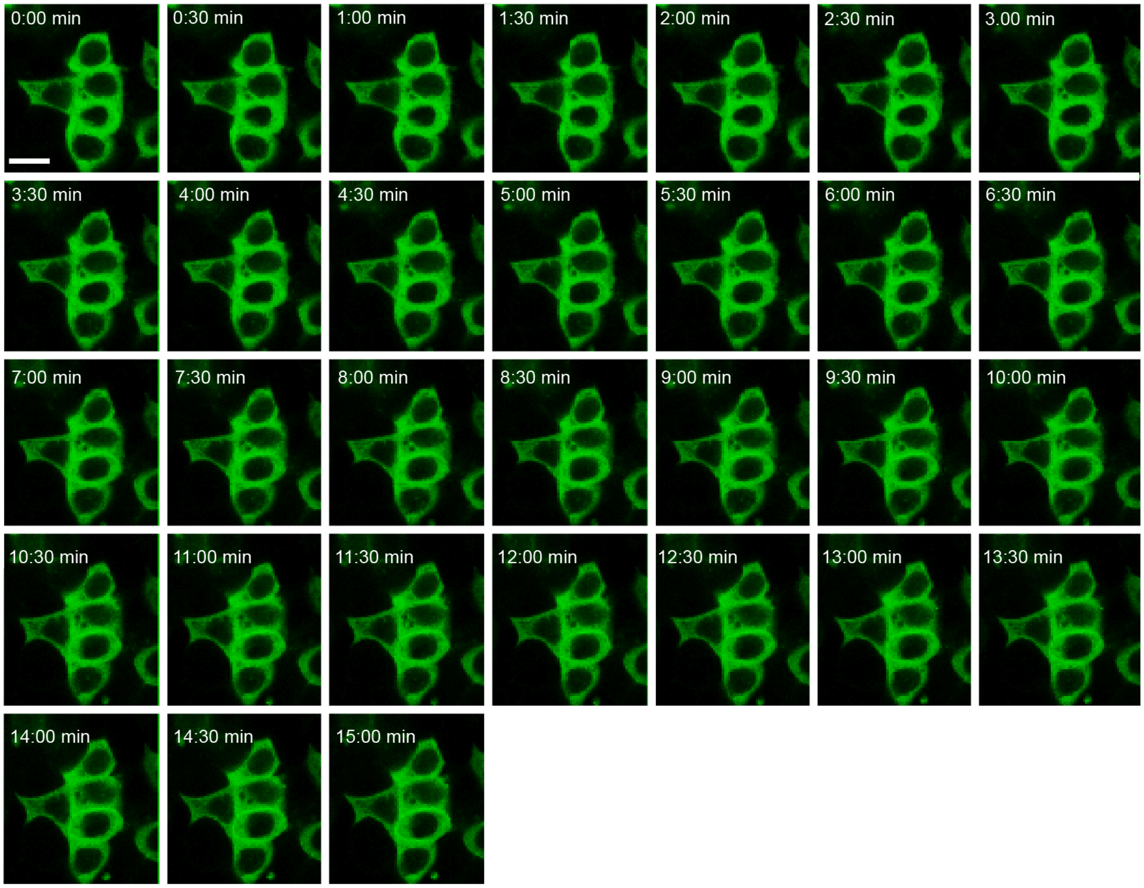

f 50 nM PMA with 5 μM ENDX pretreatment (1h)

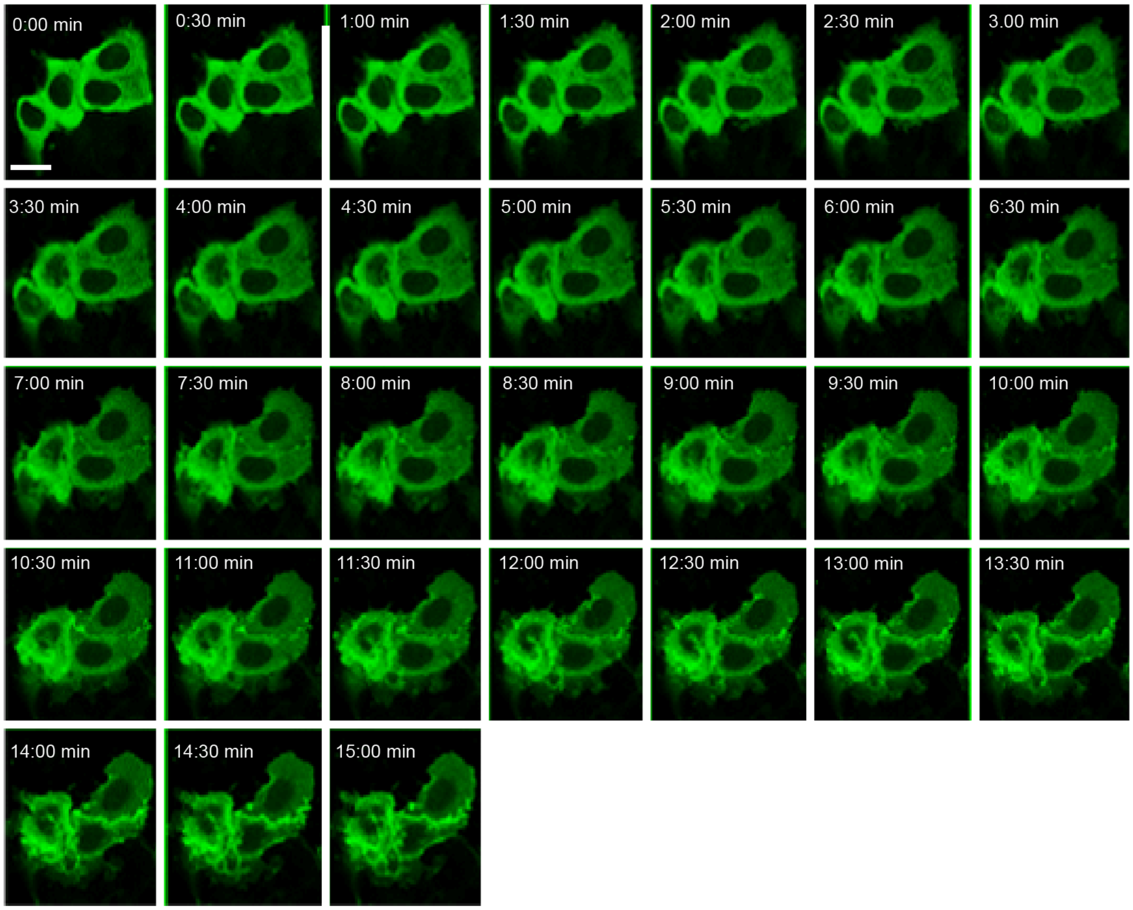

Supplementary Figure 9 Spatiotemporal distribution YFP-PKCβ1 in MCF7AC1 cells. Fluorescent images of 3 replicate time-lapse experiment recordings of YFP-PKCβ1cellular localization after addition of 50 nM PMA without (**a,c,e**), and with (**b,d,f**) 5 μM ENDX pretreatment (1 hr). Images were acquired continuously, with intervals of 30 secs between two frames. Time zero is defined as the time of PMA application and scanning time points indicated in upper left of each frame. Scale bar is 20 μm.

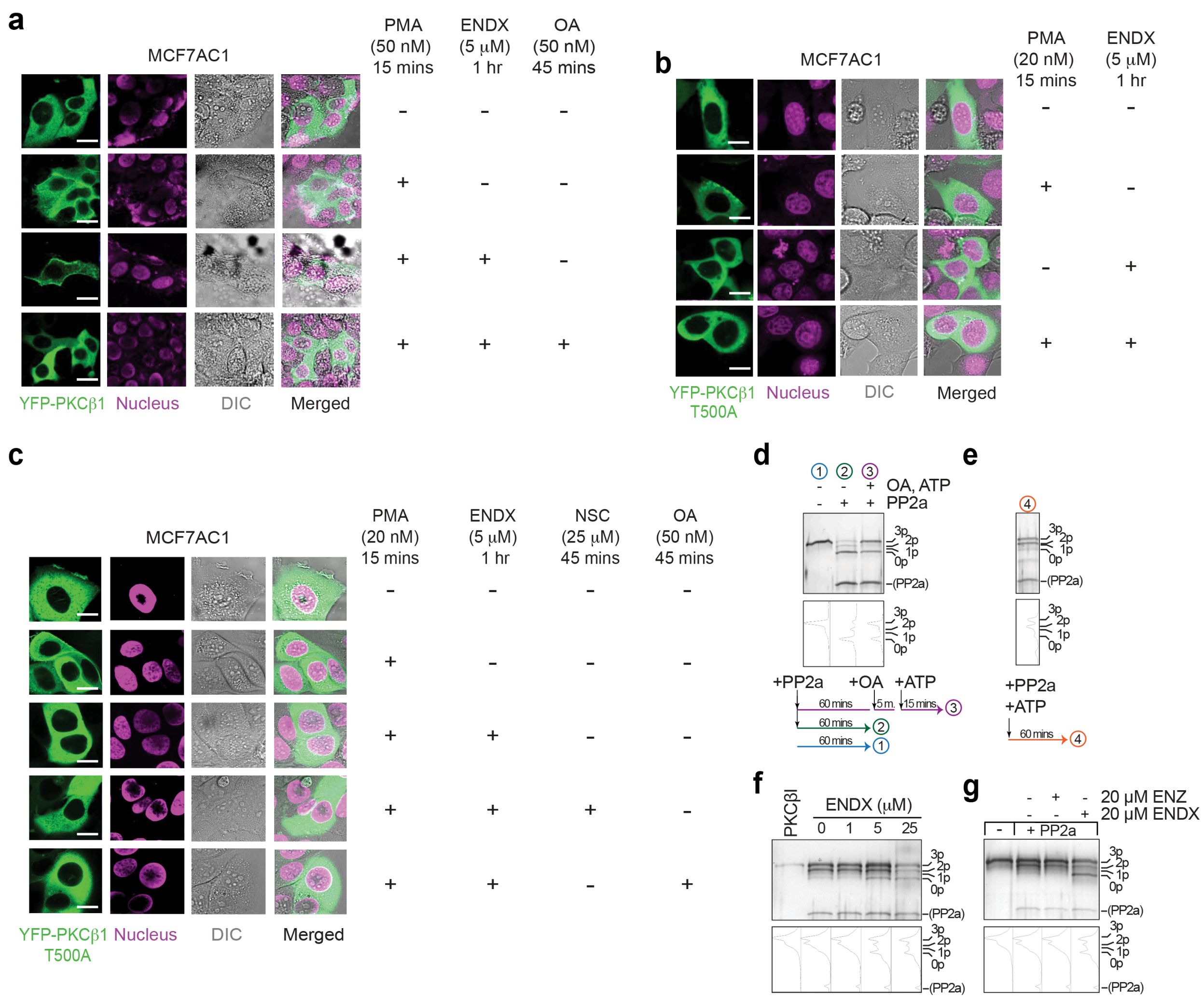

Supplementary Figure 10 (**a**) Confocal live cell microscopy images showing the effect of the PP1/PP2A inhibitor okadaic acid (OkA) on accumulation of YFP-tagged PKCβI in the presence of indicated compounds. Scale bar = 20 µm. Representative images from 3 experiments shown. (**b**) Confocal live cell microscopy images showing YFP-tagged T500A mutant PKCβI does not accumulate at the plasma membrane in the presence of indicated compounds. Scale bar = 20 µm. Representative images from 3 experiments shown. (**c**) Confocal live cell microscopy images showing YFP-tagged T500A mutant PKCβI plasma membrane accumulation is not affected by the indicated phosphatase inhibitors. NSC is the abbreviation of NSC117079 which is a pan-PHLPP inhibitor selective for PHLPP1 and PHLPP2. Scale bar = 20 µm. Representative images from 3 experiments shown. (**d**) Recombinant PKCβI was treated as described (OA = okadaic acid, PP2a = protein phosphatase 2a) and run on a 6% acrylamide 12.5uM Phos-tag gel phos-tag SDS-PAGE, followed by silver staining total protein. Bands that contain 3, 2, 1, or 0 phosphoresidues are indicated as 3p, 2p, 1p, or 0p. The lane profile is plotted below each lane. (**e**) Recombinant PKCβI was incubated with both phosphatase and ATP for 60 minutes then run on a phos-tag SDS PAGE followed by silver staining total protein. Bands are labelled and lane profiles are shown as in panel (d). (**f**) ENDX was added to phosphorylation equilibrium reactions, which were then run on a phos-tag SDS PAGE followed by silver staining total protein. Bands are labelled and lane profiles are shown as in panel (d). (**g**) ENDX or ENZ (enzastaurin) were added to phosphorylation equilibrium reactions, which were then run on a phos-tag SDS PAGE followed by silver staining total protein. Bands are labelled and lane profiles are shown as in panel (e). Source data are provided as a Source Data file.

Supplementary Table 1. X-ray diffraction data collection and structure refinement statistics

	PKCβII	PKCβI-form 1	PKCβI-form 2	PKCβI-form 1-Mn
PDB Accession #	8SE1	8SE4	8SE3	8SE2
Space group	C 1 2 1	C 1 2 1	C 1 2 1	C 1 2 1
Cell dimensions				
a, *b*, *c* (Å)	130.6, 89.1, 160.7	162.7 157.8, 87.1	82.2, 163.2, 77.3	162.7, 157.9, 85.1
α, β, γ (°)	90, 107.1, 90	90, 113.0, 90	90, 100.4, 90	90, 112.2, 90
Resolution (Å)[a]	50.00-3.35 (3.47-3.35)	50-2.7 (2.80-2.70)	50.00-2.60 (2.69-2.60)	50-2.95 (3.06-2.95)
R_{merge}	0.299 (0.962)	0.141 (2.084)	0.149 (1.485)	0.115 (1.12)
$I / \sigma I$	4.7 (1.5)	15.7 (1.1)	13.2 (1.4)	11.3 (1.3)
$CC_{1/2}$	0.91 (0.46)	0.99 (0.48)	0.99 (0.49)	0.99 (0.48)
Completeness (%)	99.9 (99.3)	99.5 (99.8)	99.2 (99.1)	98.8 (99.0)
Redundancy	3.5 (3.7)	10.6 (10.6)	6.9 (7.1)	3.6 (3.6)
Refinement				
Resolution (Å)	48.75-3.32	49.88-2.70	48.66-2.60	49.70-2.95
No. reflections	25776	55648	30355	41359
R_{work} / R_{free}	0.199/0.256	0.206/0.258	0.197/0.227	0.196/0.252
No. atoms (non-H)	10226	9685	4970	9713
Protein	10077	9657	4871	9657
Ligand/ion	84	27	28	48
Water	65	1	71	8
Average *B* factor (Å^2)	63.31	113.8	78.34	99.5
Ramachandran plot: Favored (%)	91.6	93.4	94.4	94.4
Allowed (%)	8.1	6.4	5.1	5.4
Outliers (%)	0.3	0.2	0.5	0.2
r.m.s. deviations				
Bond lengths (Å)	0.003	0.006	0.002	0.005
Bond angles (°)	0.605	0.863	0.519	0.805

[a]Values in parentheses refer to the highest resolution shell

Supplementary Table 2. SAXS data analysis parameters (Source data are provided as a Source Data file)

(*a*) Sample details			
	PKCβI, AMPPNP	PKCβI, AMPPNP, ENDX	PKCβI, AMPPNP, TAM
Organism	Homo sapiens	Homo sapiens	Homo sapiens
Source	HEK293F cells	HEK293F cells	HEK293F cells
Description - sequence (including tags) + bound ligands/modifications, etc.	PKCβI, 1mM AMPPNP	PKCβI, 1mM AMPPNP, 40 µM ENDX	PKCβI, 1mM AMPPNP, 40 µM TAM
Extinction coefficient (M^{-1} cm^{-1})	77,810	77,810	77,810
M from chemical composition	77,421	77,794	77,792
Solvent details	20mM Tris pH 8.0, 100mM NaCl, 2mM MgCl2, 1mM TCEP, and 1% (v/v) glycerol		
(*b*) SAXS data collection parameters			
Source, instrument and description or reference	APS beamline 12.3.1 (Sibyls)		
Wavelength	1.127 Å		
Detector distance	2,100 mm		

q-measurement range (Å^{-1})	0.01406 – 0.4464
Exposure time, number of exposures	0.05s, 33 exposures
Sample temperature	10 ˚C

(*c*) Software employed for SAXS data reduction, analysis and interpretation

SAXS data reduction merging	Frameslice
Calculation of extinction coefficient	Protparam
Basic analyses: Guinier, *P*(*r*)	ATSAS suite
Atomic structure modelling (rigid body, ensemble)	ATSAS suite
3D graphic model representations	Pymol

(*d*) Structural parameters

	PKCβI, AMPPNP	PKCβI, AMPPNP, ENDX	PKCβI, AMPPNP, TAM
PKCβI, AMPPNP			
I(0) (cm^{-1})	4.13 ±0.10	3.19 ±0.10	4.08 ±0.13
R_g (Å)	33.4 ±1.2	29.3 ±1.3	32.5 ±1.4
sR_g range	0.47-1.30	0.48-1.28	0.63-1.30
Fidelity (from GNOM)	1.00	1.00	1.00
M from *I*(0) (ratio to expected value)			
P(*r*) analysis			
I(0)	4.08 ±0.08	3.29 ±0.07	4.09 ±0.08
R_g	33.1 ±0.7	31.1 ±0.6	32.9 ±0.7
d_{max}	109	96	109
q range	0.0141-0.2436	0.0148-0.2725	0.0171-0.2573
Quality-of-fit parameter (total estimate)	0.592	0.759	0.681

(*e*) Atomistic modelling

Sample	PKCβI, AMPPNP	PKCβI, AMPPNP
Program used	CRYSOL	EOM
q range for fitting	0.01406 – 0.4464	0.01406 – 0.4464
Symmetry assumptions	None (P1)	None (P1)
χ^2 value	0.37	0.18
Adjustable parameters in the model fit	none	none
Modelled regions	8SE4 chain A (19–89,101–292), Chain B (336–671)	Fixed: 8SE4 chain A (19–89,101–292), Chain B (336–671). Modelled: (1–18, 90–100, 293–335)

Supplementary Table 3. Oligonucleotide sequences used for PCR primers in this study.

Primer name	Sequence 5' to 3'
PKCB1 and PKCB2 LIC Fwd	TACTTCCAATCCAATGCTGACCCGGCTGCG
PKCB1 LIC Rev	TTATCCACTTCCAATGTAACTACACATTAATGACAAACTCTGGGTT
PKCB2 LIC Rev	TTATCCACTTCCAATGTTACTAGCTCTTGACTTCGGGTTTTAAAAA
E282R Fwd	CTGAGCCAGGAGCGAGGCGAGTACTT
E282R Rev	AAGTACTCGCCTCGCTCCTGGCTCAG
Y285R Fwd	GAGGAAGGCGAGCGCTTCAATGTGCC
Y285R Rev	GGCACATTGAAGCGCTCGCCTTCCTC
F286E Fwd	GAAGGCGAGTACGAGAATGTGCCTGTG
F286E Rev	CACAGGCACATTCTCGTACTCGCCTTC
K439E Fwd	GTCGGCCGGTTCGAGGAGCCCCATGCT
K439E Rev	AGCATGGGGCTCCTCGAACCGGCCGAC
A570E Fwd	AGGAAGCTGTGGAAATCTGCAAAGGG
A570E Rev	CCCTTTGCAGATTTCCACAGCTTCCT
Y602E Fwd	CATTTTTCCGGGAGATTGATTGGGAG

Y602E Rev	CTCCCAATCAATCTCCCGGAAAAATG
G24Q Fwd	CGTGCGCTTCGCCCGCAAACAAGCCCTCCGGCAGAAGAACGT
G24Q Rev	ACGTTCTTCTGCCGGAGGGCTTGTTTGCGGGCGAAGCGCACG
Prescission site C2-Cat PCKβ1 Primer B Rev	TTGGTCGTCTTTTCTTCCGGTCCTTGGAATAGTACTTCGAG
Prescission site C2-Cat PCKβ1Primer C Fwd	AGAAATTTGAGAGGGCCAAGCTCGAAGTACTATTCCAAGGA
PKCB1 Primer B Rev PS Prescission site	TCCGCTCGGTCCTTGGAATAGTACTTCGAGGCTTCCGTTCTTCTGCCG GAGGGCGCCAAA
PKCB1 Primer C Fwd PS Prescission site	GGAAGCCTCGAAGTACTATTCCAAGGACCGAGCGGAGTGCATGAGGT CAAGAACCACAA
