# Supplementary material for: Molecular basis of allosteric regulation and pharmaceutical targeting of protein kinase Cβ
Source: Nat Commun. 2026 May 21;17:6703. doi: 10.1038/s41467-026-73413-5 (PMC13385876; doi:10.1038/s41467-026-73413-5)
Supplement: Supplementary file 2 — Reporting Summary [file 41467_2026_73413_MOESM2_ESM.pdf]

nature portfolio

Corresponding author(s): Matthew Schellenberg, Matthew Goetz

Last updated by author(s): Dec 31, 2025

Reporting Summary

Nature Portfolio wishes to improve the reproducibility of the work that we publish. This form provides structure for consistency and transparency in reporting. For further information on Nature Portfolio policies, see our Editorial Policies and the Editorial Policy Checklist.

Statistics

For all statistical analyses, confirm that the following items are present in the figure legend, table legend, main text, or Methods section.

n/a	Confirmed	
☐	☒	The exact sample size (*n*) for each experimental group/condition, given as a discrete number and unit of measurement
☐	☒	A statement on whether measurements were taken from distinct samples or whether the same sample was measured repeatedly
☐	☒	The statistical test(s) used AND whether they are one- or two-sided<br>*Only common tests should be described solely by name; describe more complex techniques in the Methods section.*
☒	☐	A description of all covariates tested
☒	☐	A description of any assumptions or corrections, such as tests of normality and adjustment for multiple comparisons
☐	☒	A full description of the statistical parameters including central tendency (e.g. means) or other basic estimates (e.g. regression coefficient) AND variation (e.g. standard deviation) or associated estimates of uncertainty (e.g. confidence intervals)
☐	☒	For null hypothesis testing, the test statistic (e.g. *F*, *t*, *r*) with confidence intervals, effect sizes, degrees of freedom and *P* value noted<br>*Give P values as exact values whenever suitable.*
☒	☐	For Bayesian analysis, information on the choice of priors and Markov chain Monte Carlo settings
☒	☐	For hierarchical and complex designs, identification of the appropriate level for tests and full reporting of outcomes
☒	☐	Estimates of effect sizes (e.g. Cohen's *d*, Pearson's *r*), indicating how they were calculated

Our web collection on statistics for biologists contains articles on many of the points above.

Software and code

Policy information about availability of computer code

Data collection	HKL2000 (V720), Unicorn (7.3), Clariostar (5.61), Zeiss Zen Blue (3.6)
Data analysis	Pymol (2.5.2), Phenix (1.17.1-3660), Graphpad Prism (10.0.3), HKL2000 (V720), Coot (0.8.9.1), Primus (3.03), FIJI (2.1.0), Imagestudio (5.2.5), Bio-Rad CFX Manager (3.1), Crysol (30), EOM (30), FIJI ImageJ (2.1.0/1.53c)

For manuscripts utilizing custom algorithms or software that are central to the research but not yet described in published literature, software must be made available to editors and reviewers. We strongly encourage code deposition in a community repository (e.g. GitHub). See the Nature Portfolio guidelines for submitting code & software for further information.

Data

Policy information about availability of data

All manuscripts must include a data availability statement. This statement should provide the following information, where applicable:

- Accession codes, unique identifiers, or web links for publicly available datasets
- A description of any restrictions on data availability
- For clinical datasets or third party data, please ensure that the statement adheres to our policy

All unique/stable reagents generated in this study are available from the lead contact with a completed materials transfer agreement. Atomic coordinates and structure factors have been deposited in the Protein Data Bank (www.rcsb.org) under accession codes 8SE1 (PKCβII) A) [http://doi.org/10.2210/pdb8SE1/pdb], 8SE2 (PKCβI form 1) [http://doi.org/10.2210/pdb8SE2/pdb], 8SE3 (PKCβI form 2) [http://doi.org/10.2210/pdb8SE3/pdb], and 8SE4 (PKCβI-Mn complex) [http://doi.org/10.2210/pdb8SE4/pdb].

Research involving human participants, their data, or biological material

Policy information about studies with human participants or human data. See also policy information about sex, gender (identity/presentation), and sexual orientation and race, ethnicity and racism.

Reporting on sex and gender	n/a
Reporting on race, ethnicity, or other socially relevant groupings	n/a
Population characteristics	n/a
Recruitment	n/a
Ethics oversight	n/a

Note that full information on the approval of the study protocol must also be provided in the manuscript.

Field-specific reporting

Please select the one below that is the best fit for your research. If you are not sure, read the appropriate sections before making your selection.

[x] Life sciences [ ] Behavioural & social sciences [ ] Ecological, evolutionary & environmental sciences

For a reference copy of the document with all sections, see nature.com/documents/nr-reporting-summary-flat.pdf

Life sciences study design

All studies must disclose on these points even when the disclosure is negative.

Sample size	No sample size calculation was performed to pre-determine sample sizes. A minimum sample size of 3 replicates was chosen for enzymatic assays to provide an adequate estimate of error associated with each data point in an assay. When adequate reagents were available a fourth replicate was included.
Data exclusions	A pre-determined criterion was applied in enzyme assays to exclude data showing obvious deviations from other technical replicates, or inconsistent readings that precluded slope measurement needed to calculate rates, which is often caused by air bubbles in assay wells.
Replication	Experiments were repeated at least twice and similar results were obtained.
Randomization	n/a
Blinding	n/a

Reporting for specific materials, systems and methods

We require information from authors about some types of materials, experimental systems and methods used in many studies. Here, indicate whether each material, system or method listed is relevant to your study. If you are not sure if a list item applies to your research, read the appropriate section before selecting a response.

Materials & experimental systems

n/a	Involved in the study	
[ ]	[x]	Antibodies
[ ]	[x]	Eukaryotic cell lines
[x]	[ ]	Palaeontology and archaeology
[x]	[ ]	Animals and other organisms
[x]	[ ]	Clinical data
[x]	[ ]	Dual use research of concern
[x]	[ ]	Plants

Methods

n/a	Involved in the study	
[x]	[ ]	ChIP-seq
[x]	[ ]	Flow cytometry
[x]	[ ]	MRI-based neuroimaging

Antibodies

Antibodies used	Anti PKCβ phospho-T500 (Abcam ab5817, lot GR173447-1)<br>Anti-phospho T642 (Abcam ab75657, lot GR29222-17)<br>anti-phospho S661 (Abcam 192184, lot GR224655-1)

Validation	Antibodies were validated by supplier

Eukaryotic cell lines

Policy information about cell lines and Sex and Gender in Research

Cell line source(s)	HEK293F (Thermofisher R79007)<br>MCF7AC1 (Dr. Angela Brodie, University of Maryland)
Authentication	Cell lines used in this study were not authenticated by our group
Mycoplasma contamination	Cell lines tested negative with Lonza Micoalert plus kit prior to initiating experiments
Commonly misidentified lines<br>(See ICLAC register)	No commonly misidentified cell lines were used in this study

Plants

Seed stocks	n/a
Novel plant genotypes	n/a
Authentication	n/a
