# Supplementary material for: Molecular basis of allosteric regulation and pharmaceutical targeting of protein kinase Cβ
Source: Nat Commun. 2026 May 21;17:6703. doi: 10.1038/s41467-026-73413-5 (PMC13385876; doi:10.1038/s41467-026-73413-5)
Supplement: Supplementary file 3 — Transparent Peer Review file [file 41467_2026_73413_MOESM3_ESM.pdf]

nature portfolio

Peer Review File

Molecular Basis of Allosteric Regulation and Pharmaceutical Targeting of Protein Kinase Cβ

Corresponding Author: Dr Matthew Schellenberg

This file contains all reviewer reports in order by version, followed by all author rebuttals in order by version.

Attachments originally included by the reviewers as part of their assessment can be found at the end of this file.

Version 0:

Reviewer comments:

Reviewer #1

(Remarks to the Author)
This paper features multiple structures of full-length PKC I and II in active and inactive states. The structures are at good resolution and well-refined, and therefore highly credible. This is an important and long-awaited structural tour-de-force worthy of publication in a high profile venue. The discussion is clear and thorough. The functional aspects of the paper are much less impressive. A natural assessment of the structures would be to mutate the interface and explore the dose-response for translocation in cells or activation on liposomes. To some extent this type of information for the kinase-C2 interface is provided in Fig. 2c. It would be preferable for this type of experiment to be shown for the other interfaces. It would be much more informative to see dose-response instead of total activity at a single condition. The experiment in Fig. 3 does not show anything that wasn't known by the late 1980s, and the single mutant studied in Fig. 4c is not a very complete analysis. In the interests of getting these fascinating structures into the public view faster, perhaps such studies could be left for a follow-up paper.

Introduction, line 5, and elsewhere, substitute "conventional" PKC for "canonical"

Fig. S2 Legend. "Experimental" in reference to electron density syntheses is ambiguous. Specific the phasing model. Were the models shown including in the phasing of the corresponding maps, i.e., are these omit maps, or not?

Fig. 2d should show electron density. An anomalous difference map would be desirable as well.

Ref. 21 should be to Violin et al. 2003.

Reviewer #2

[Editorial Note: Please also see attachment at end of file]

(Remarks to the Author)
Summary

Protein Kinase C (PKC) is a family of 10 mammalian iso-enzymes that transduce the signal generated by phospholipase C (PLC) into appropriate downstream responses in the cell. PKCs have been implicated in a number of disease pathologies and regulate a diverse array of critical intracellular signaling pathways that make the study of their regulation incredibly valuable. Unfortunately, decades of study have failed to elucidate the complex regulatory mechanism(s) of these kinases at atomic resolution. The authors of this manuscript present a series of X-ray crystal structures of two splice variants of PKCb, which they use, together with a series of accompanying biochemical and cell biological experiments, to rationalize the mechanism of PKC activation by calcium, phospholipids, and diacylglycerol (DAG). In addition, the authors report the

characterization of endoxifen, a metabolite of the anti-cancer drug tamoxifen, as an allosteric inhibitor of PKCb. The topic is of broad interest to a general audience, including basic, translational and clinical scientists. The study, however, falls short in a number of areas and, in the opinion of this reviewer, there are significant reasons to doubt some of the conclusions that the authors make on the basis of their data. In particular, serious concerns are raised about the quality of some of the supporting biochemical and biophysical data, which the manuscript heavily depends on to support the authors' structure-based hypotheses.

Major Comments

Structure of PKCbII reveals architecture of autoinhibited state.

The authors have determined the structure of full-length PKCbII in which 2 chains are observed in the ASU. The first chain (chain A, presumably) forms an intramolecular complex in which electron density is visible for 91% of the chain, while the second chain forms a domain-swapped interface between its regulatory domains and the kinase domain of an adjacent protomer in the crystal lattice. In this second instance, the inter-molecular interface is near-identical to the intramolecular interface observed in chain A. The structure represents the most complete picture of any PKC isoform to date.

The catalytic cleft of each kinase domain is engaged by a pseudosubstrate segment (PS) (either its own, or that of its domain swap partner), consistent with the known mode of substrate:kinase interaction. The DAG binding surface of the C1b domain is observed to be buried in intramolecular contacts with the PS and kinase domain, in contrast to a previously reported structure of PKCbII in which the C1b domain was observed to form an intramolecular contact with the N-lobe of the kinase domain (Leonard et al., Cell 2011), an interface that was validated with membrane translocation assays in cells both in the original manuscript and in a follow-up manuscript published by the same group (Lucic et al, JMB 2016).

In the original submission of this manuscript, it was difficult to assess the physiological relevance of the reported C1b-PS-kinase interface in the absence of any mutational data. The authors have added kinase activity data on a mutant, G24Q (Supplementary Figure 3D) to substantiate this interface. Although the data presented supports their claims, the data points have no error bars (presumably n=1) and the y-axis scale is '% kinase activity', where it appears that the authors have normalized their data to the signal from the highest wt concentration. At the very least, this experiment needs to have sufficient replicates and an appropriate activity scale.

The DAG-binding surface of the C1a domain is observed to be solvent accessible. The observation that F43 of the C1a domain is buried in an intramolecular interface with the kinase domain is consistent with a previously published report showing that its mutation results in accelerated membrane translocation of all conventional and novel PKCs (Lučić et al, JMB, 2016). Stabilization of the active conformation of the kinase domain and its intramolecular interactions by phosphorylation of T500, T641 and S660, however, was already known, including how mutation of these sites to non-phosphorylatable alanines causes PKC to partition into the insoluble or membrane-bound fraction.

Crystal structures of PKCbI reveal active and inactive states.

PKCbI and PKCbII are slice-variants that differ only in the C-terminal ~50 amino acid tail of their kinase domains. The authors report two more crystal structures, this time of PKCbI in two different crystal forms.

Crystal form 1 contains 2 mol/ASU in which one of the two chains is observed to have electron density for the PS in the catalytic cleft of the adjacent protomer, again via a domain swap. The arrangement of the regulatory domains in this crystal form seems to be incompatible with the inactive conformation that they report for PKCbII, and the authors investigate this arrangement later in the context of PKCbI activation.

Crystal form 2 contains 1 mol/ASU in which the PS segment is not observed bound to the catalytic cleft, despite it being unobstructed and available for engagement. The authors suggest that the packing of the regulatory domains with the kinase domain is similar to that observed in PKCbII, but this is difficult to assess based on the data presented. Visually, these structures do not appear to be that similar and the authors themselves state that the C1b domain occupies "an altered location…that we attribute to the autoinhibited conformation for the PKCbI enzyme." It is not clear what the authors mean by this, but it is difficult to imagine different autoinhibited conformations for PKCbI and PKCbII given that they are identical except for their C-terminal tails. Here, a definitive analysis of their claims is somewhat impeded by a more quantitative comparison of the PKCbII and PKCbI (crystal form 2) structures. Given that the PS segment is not observed in the catalytic cleft and the C1b domain occupies a different position, it should at least be considered that the arrangement of the domains could be the consequence of lattice packing interactions that are energetically more favorable.

The authors observe an intriguing interface between the regulatory domains of PKC and its kinase domain that is common to both PKCbI structures that they have determined. In this conformation, which the authors refer to confusingly as N-term 1, the C1a, C1b and C2 domains pack against the C-lobe of the kinase domain. They make an interesting comparison to the AF2 prediction of PKCb, which places the domains in a similar arrangement. The authors have added in the AF2 prediction for the PKCa isoform, but there are still no PAE plots included in Supplementary Figure 5e, which would offer a metric-based assessment of the confidence of the predictions. Nevertheless, prediction of the same arrangement for other PKCs and the strong similarity to their experimentally determined structure strengthens the authors' claims.

PKCbI active conformation

The authors next investigate the interface between the N-terminal domains and the C-lobe of the kinase that they observed in both crystal forms and that is also predicted by AF2. Given that the AF2 predictions of PKCb and PKCa are convergent with the authors' experimentally determined arrangement of the regulatory domains, the implications are intriguing. Namely, that the membrane binding surfaces of the C1a, C1b, and C2 domains are observed in the same plane and binding of the PS segment to the kinase domain would be impossible. Should this structure represent the active conformation of PKCbI, it would help resolve two important questions regarding PKC activation: (1) does PKC form a defined, active conformation on the membrane (implications for substrate specificity etc) and (2) what prevents the PS segment from inhibiting activity on the membrane?

The authors attempt to validate this interface with a site-directed mutagenesis approach coupled to an in vitro FRET-based kinase assay (why such a complicated kinase assay readout is required in vitro is not clear – other, more classical in vitro assays would almost certainly provide a cleaner readout). They report a series of mutants in the observed C1b-kinase and C2-kinase interfaces that impair PKC activity in vitro. However, the effect is modest for all mutants, except for F286E, which (strangely) seems to exhibit no concentration-dependent change in activity (Figure 2C). Additionally, the observed packing arrangement of the domains exclusively involves the distal surface of the C-lobe of the kinase domain, which is not a reported surface of allosteric regulation for PKC. In fact, there are no movable elements in that surface that could regulate kinase activity allosterically. It is therefore hard to reconcile changes in activity in vitro caused by mutations that would disrupt these interfaces. The authors do not discuss this apparent contradiction, although, as previously stated, the interface they observe is potentially relevant. The question therefore arises: what is the interface useful for? In this respect, I believe that the authors may want to consider more explicitly the implications of the interface for the conformation and substrate specificity of PKC on the membrane. The consequences of such an interface for maintaining active and very highly specific PKC on the membrane due to a well-ordered conformation are quite profound, in my opinion, and are possibly the most interesting findings of this study.

I do not, therefore, dismiss a physiological relevance of the interface observed. Since the autoinhibited conformation of PKCb relies on the clamping of the PS segment by the C1b domain (Figure 1), a possible way to test this interface would be to examine the interface mutants in the context of a construct of PKCb lacking its PS segment. Kinase activity assays are unlikely to reveal allosteric changes for the aforementioned reasons, but the particle might reasonably be expected to remain compact and become extended upon disruption of the interface. This could be tested, for example, by SAXS analysis of PKCb interface mutants compared to PKCb, assuming that the recombinant proteins behave sufficiently well to make them amenable to such analyses.

The lipid lever mechanism of PKC activation

Having determined that the above conformation is, according to their mutagenesis data, biochemically relevant, the authors sought to rationalize the activation of PKC on membranes containing DAG. DAG-mimetics, such as phorbol esters, are commonly used to activate PKCs in vitro. However, as the authors show, they rely on being embedded in a membrane for their ability to activate PKC (Figure 3B). The authors assert that, due to solvent accessibility of the lipid binding surface of the C1a domain in their crystal structure, phorbol dibutyrate (PDBu; a DAG-mimetic) binding would not disrupt the inactive conformation and therefore a direct competition mechanism can be ruled out. I strongly disagree with this statement for the following reasons:

1. The PDBu-binding surface of the C1B domain in their structure is buried in an intramolecular interface, which means that PDBu would compete for this interface.
2. Multiple reports have unambiguously determined that the DAG-binding surfaces of the C1 domains (minimally one, but possibly both) are sequestered in the intramolecular, autoinhibited conformation. In fact, the naked C1 domains translocate to the plasma membrane with dramatically accelerated kinetics compared to full-length PKCs (Oancea & Meyer, Cell 1998; Lucic et al, JMB 2016).
3. Mutation of F43 (or its equivalent) in the C1a domain essentially unlocks all novel and conventional PKCs such that they translocate with near-identical kinetics to the isolated C1a-C1b domains. While an allosteric effect on the neighboring C1b domain and PS cannot be excluded, this observation strongly suggests that the DAG-binding surface of the C1a domain is also sequestered in the autoinhibited conformation. This raises the question of whether the interface observed in the authors' crystal structure is representative of the physiological conformation. Given the reported promiscuity of domain swaps in PKCb crystal lattice formation, this cannot be ignored.

The authors use a limited proteolysis assay to probe PKCbI conformation in the presence of Ca2+, PDBu or lipids, and combinations thereof. They show, in Figure 3C, that DAG-containing lipid micelles result in increased elastase-mediate cleavage of PKCbII (presumably in the C2-kinase domain linker?), whereas PDBu and Ca2+ are insufficient. This supports the requirement for DAG/PDBu to be embedded in a membrane/micelle, but it does not rule out a competition mechanism between DAG/PDBu and intramolecular contacts of the C1 domains with the kinase domain.

Minor comments related to Fig. 3C:
• Why do the authors compare PDBu with DAG-containing lipid micelles? This is a bit like comparing apples with oranges.

Minor comments regarding the text for this section:

The authors propose what they call a 'lipid lever model', in which they suggest that the membrane acts as a lever to pry the regulatory domains apart from the kinase domain. The model is based on the incompatibility of the domain arrangement

observed in the autoinhibited structure of PKCbII with membrane binding by the C1a domain, which would cause a steric clash between the kinase domain and the membrane. Whilst the phrasing of this has been improved from the previous version of this manuscript, I urge the authors to reconsider their terminology here. If the C1a domain cannot approach the membrane because a of a clash between the membrane and the kinase domain, then binding is prohibited until the two domains dissociate (as the authors themselves write). As such, this model is not a 'lipid lever' model, which implies a force, but rather a process of conformational selection.

Although the authors use previously published membrane translocation data to support their lipid lever model, this is also problematic. In fact, published mutational data on F43, which appears to completely unlock the autoinhibited conformation of PKC (Lucic et al, JBC 2016), supports a conformational selection model in which the C1a and C1b domains must first dissociate from the kinase domain before they can bind the membrane. Here, whilst the authors have improved their terminology, their continued use of the 'lipid lever' terminology is misleading and their citation of references 14 and 17 needs to be more explicit. In particular:

1. It's not clear why reference 14 is cited after the sentence: "Additional data that supports the lipid-lever model comes from reports that the C1 domains exhibit enhanced membrane binding in the absence of the properly folded kinase domain 14, 17." Better references for this statement are the original studies by Oancea and Meyer (JBC 1998, Cell 1998).
2. The authors confusingly refer to the "absence of the properly folded kinase domain" in these studies, but what they mean is simply the absence of the kinase domain. They inadvertently imply that the proteins under investigation are not properly folded, which is not the case.
3. Reference 17 specifically deals with F43 and the equivalent residue in all conventional and novel PKCs. Since its mutation demonstrates accelerated membrane translocation of all cPKCs and nPKCs and the authors focus on it as part of their interface, it seems worthy of a more explicit explanation. In fact, this would help the authors to support their claims regarding the physiological relevance of the C1a-kinase interface they observe.

Molecular basis of isoform-specific differences between the splice variants PKCbI and PKCbII

PKCbI and PKCbII have been reported to have different affinities for phospholipid membranes. To try to rationalize this difference, the authors compared their structures of autoinhibited PKCbII (Figure 1) and PKCbI (crystal form I). The two structures differ in the following ways:

1. Electron density for the PS is only observed in the catalytic cleft of the neighboring protomer in the crystal lattice of PKCbI, an interaction brought about by a domain swap.
2. The C1b domain exhibits a different position: in PKCbII it is sequestered in an extensive contact with the catalytic domain that makes its lipid binding surface solvent inaccessible; in contrast, its lipid binding surface is solvent exposed in PKCbI.

According to the structure depicted in Figure 1D, the change in C1b position in PKCbI could reasonably be attributed to the absence of the PS in the catalytic cleft of one molecule in the ASU, an interaction which seems to be necessary for the intramolecular contacts of the C1b domain observed in PKCbII (Figure 1D). However, the authors do not appear to have considered this possibility. Instead, the authors focus on new contacts that they observe in PKCbI crystal form 1 between the C1b domain and the V5 extension to the kinase domain, which differs between PKCbI and bII. They postulate that the presence of F648 in PKCbI, which is V647 in bII, forces helix aB closer to the PS segment, thereby preventing an equivalent interaction of the C1b domain to that seen in PKCbII. To support this hypothesis, the authors engineered a F648A mutant of PKCbI and compared its sensitivity to elastase (as a probe for the active conformation) in a limited proteolysis assay. PKCbI was observed to be more sensitive to elastase cleavage than PKCbII in the presence of lipids. The following issues preclude a definitive assessment:

- It is unclear why the authors make a F648A mutant rather than F648V, which would convert F648 into the equivalent residue in PKCbII. As a test for the difference, this would seem to be a more appropriate comparison.
- n=1 for the data shown in Figure 4C. There is no quantifiable difference in elastase sensitivity in the absence of lipids (middle panel), while each protein measured in panel C is performed only once. What has been quantified by densitometry is also not described in the figure legend and the effect size is modest at best (6% of PKCbII cleaved vs 11% of PKCbI and 3% of the F648A mutant). Since this lacks replicates and corresponding error bars, it does not provide compelling evidence to support the authors' conclusions.
- This figure/data has not changed from the original manuscript.

Endoxifen as an allosteric regulator of PKCb

The final experimental section of this manuscript addresses published data reporting an inhibitory effect of tamoxifen (TAM) on PKC and a more potent effect of its metabolite, endoxifen (ENDX). The authors start by assessing the inhibitory effect of TAM and ENDX on PKCbI and bII in an in vitro kinase assay. Both compounds exhibited lower IC50 values in the absence of lipids; however, given the hydrophobicity of these compounds, indirect effects of lipids on their availability in solution cannot be ruled out. As such, the interpretation of the results in Figure 5A should be treated cautiously.

The authors also report thermal stability (DSF) measurements for PKCbI in the presence and absence of ENDX (Figure S7A). Although they claim that ENDX binds to both the N-terminal regulatory and kinase domains of PKCbI (pg 7, ln 15-17), the data do not support this conclusion. In fact, the melting temperature of full-length PKCbI (FL), the catalytic domain (CAT), and the N-terminal domain (NT) are unaffected (even slightly reduced) by ENDX (see table inset, Figure S7A). It is, therefore, not clear how the authors came to the conclusion that ENDX binds to PKC from this DSF data. The data and the

conclusions from these experiments remain unchanged from the original manuscript, but do not support the authors conclusion that “differential scanning fluorimetry (DSF) analysis shows that both the N-terminal regulatory domain and the kinase domain bind to ENDX.”

Next, the authors support the conclusion that ENDX is an allosteric inhibitor of PKCbI by comparing the effect of ENDX on PKCbI in the presence or absence of lipids with the isolated kinase domain of PKCb (Figure 5B). ENDX has a stronger inhibitory effect on PKCbI in the absence of lipids than either PKCbI with lipids or the isolated catalytic domain, which the authors interpret as an allosteric effect of ENDX on PKCbI that depends on the intramolecular assembly of its regulatory domains. (Again, I would emphasize the aforementioned concerns regarding the hydrophobicity of ENDX and possible (indirect) effects of lipids on its inhibitory behavior). This is further supported by kinase activity data in Figure 5C that shows that ENDX is a non-competitive ATP inhibitor (it lowers Vmax without changing the Km for ATP, right panel), which is in contrast to the known ATP-competitive inhibitor enzastaurin, which does not affect Vmax, but increases the Km for ATP (left panel).

Minor comment: panels A and C each contain two graphs, which should be labeled separately for ease of reading and interpretation.

To evaluate the effect of ENDX on the conformation of PKCbI in solution, the authors employed small angle X-ray scattering (SAXS). Figure 5D depicts the raw 2D scattering curves for PKCbI alone, +TAM and +ENDX, together with a table of the respective Rg and Dmax parameters derived from the data. The authors have added Guinier plots for their SAXS data (Supplementary Figure 7d) which confirm that their samples are generally well behaved, with the exception of their PKCbI+TAM sample, which shows evidence of low levels of aggregation (therefore Rg and Dmax should be treated cautiously). The manuscript also now contains a Materials and Methods section on the SAXS sample preparation and data collection, as well as a Supplementary Table summarizing the corresponding data parameters. Pair-distribution functions used to determine Dmax, however, are still missing. Overall, the evidence from SAXS that ENDX results in compaction of PKCbI is reasonably strong, as evidenced in the low-angle scattering regime in Figure 5D. However, the authors could make a stronger case by showing the respective Guinier plots for PKCbI ±TAM on the same plot in the main figure (only a SAXS expert will spot and appreciate the difference in the raw scattering curves that the authors show).

It is, however, somewhat perplexing that ENDX exerts an apparent compaction effect on PKCbI in solution, but has no stabilizing effect in the authors DSF assays (despite the authors’ claims to the contrary. An allosteric inhibitor, that acts as a ‘molecular glue’ to stabilize intramolecular, interdomain interactions would be expected to have a fairly significant effect on the melting temperature of FL PKCbI. However, according to the authors’ data in Supplementary Figure 7a, it has the opposite effect of lowering the melting temperature. This apparent contradiction undermines the authors’ claims that ENDX is an allosteric inhibitor of PKCbI.

Using limited proteolysis again as a readout for conformation, the authors then compare the effects of ENDX and TAM on elastase cleavage of PKCbI. No comparison to PKCbI in the absence of either compound is shown as a reference. While the data presented show a clear difference in the cleavage pattern which is visible by eye, it is not clear what sort of model has been used to fit the data in the lower panel. In particular, fitting the cleavage of PKCbI in the presence of TAM appears to be questionable given that there is no significant change at any concentration of TAM. This remains unchanged from the original manuscript.

Finally, the authors employ live-cell imaging to visualize the effect of ENDX on PKCbI translocation to the plasma membrane in MCF7AC1 cells treated with the phorbol ester PMA. The authors observed that ENDX promoted PKCbI translocation in the presence of PMA and that the PHLPP phosphatase inhibitor NSC117079 abrogated this effect (Figure 5F). Based on the authors’ previous observations of the effects of ENDX, these findings are somewhat paradoxical. The authors claim that ENDX is an allosteric inhibitor of PKC that specifically targets its intramolecular conformation in an ATP non-competitive manner and renders it more compact. Intuitively, one might therefore expect an inhibitory effect on PKC translocation, much as has been observed for the allosteric inhibitor MK2206, which targets Akt. Instead, the authors observe the opposite. The data presented in Figure 5F and Figure S7C-D is difficult to assess for the following reasons:

• the authors present only static snapshots of different patches of cells in each experiment. Without translocation kinetic data, averaged over many cells to account for variabilities in PMA and/or ENDX availability that result from the available membrane surface area and local environment of each cell in a 2D culture monolayer, it is impossible to assess with any degree of confidence what the effect of ENDX treatment is on PKCbI membrane translocation.

The authors invoke the phosphatase PHLPP as a possible explanation for this effect on the basis that the reported PHLPP inhibitor NSC117079, as well as the PP1/PP2A inhibitor okadaic acid (OA), mitigated the increased localization. However, there are significant problems with this interpretation:

• The authors use a mutant PKCbI, T500A, which cannot be phosphorylated in its activation loop. ENDX, NSC and OA had no effect on the translocation of PKCbI T500A, compared to wt PKCbI. However, the implication, at least for NSC, is that PHLPP inhibition is responsible for this effect. However, PHLPP has been reported to be the phosphatase for the hydrophobic motif of PKC and not the activation loop (T500), and the validation of NSC as a specific PHLPP inhibitor is not very convincing, in the opinion of this reviewer. Furthermore, OA specifically inhibits a structurally different family of phosphatases to PHLPP, which implies that these compounds exert different effects on cells which cannot be rationally integrated into the same model.
• The authors reference autophosphorylation of T500, but the prevailing model in the field is that this is accomplished by

PDK1. I do not necessarily subscribe to this model, but the authors should acknowledge it, since inhibition of autophosphorylation is intrinsic to their model. To support their model, the authors should demonstrate that ENDX inhibits PKCb T500 autophoshorylation in an in vitro kinase assay containing purified, recombinant, and dephosphorylated PKCbI. This should be possible given that the authors have well-behaved, purified proteins at their disposal.

Minor comment:

The authors reference Supplementary Figure 7C for the effects of OA on membrane accumulation, but the correct reference is Supplementary Figure 8C.

In summary, the assertion that ENDX is an allosteric inhibitor of PKCbI is not sufficiently well supported by the data the authors present.

Reviewer #3

(Remarks to the Author)
In their article “Molecular Basis of Allosteric Regulation and Pharmaceutical Targeting 1 of Protein Kinase Cβ” Cong et al report their crystallographic results, biochemistry results and analysis on an important class of proteins in the Protein Kinase C (PKC) family.

On the positive, the data reported in this paper are very worthwhile and meet the barrier for a high ranking journal. These protein and these crystal structures have been sought after for many decades. The resolution and completeness of these structures is very informative and has the possibility to guide further biological studies and even help in drug discovery.

The reporting of these results faces many challenges. The proteins are complex with multiple domains, there are multiple functions and finally their crystal structures are also very complicated. For such a complicated system and results, the authors must have a very clear explanation of their results and conclusions. Unfortunately, I didn’t feel like the authors hit that mark in their current draft. There were many confusing issues with the presentation of these results in this paper. These issues made the paper almost unreadable as the reader is made to look up, in other literature, whether the authors meant one thing or another. I don’t think these issues are insurmountable. They require the authors to read their paper multiple times and assess whether a person outside the lab would also understand, without second hand information. I definitely did not catch all issues as they accumulated and I got bogged down as I read the paper.

Starting from the beginning
Phosphorylation is required for proper folding. Don’t think this is true, protein folds and phosphorylation is part of function and regulation.

PBI is used once in the paper without definition.

Very little is said about the active site of the protein. Sure this may not be the main point of the paper, but a sentence that comments on the active site of the protein and says the results are identical to some other literature would be good. Worth paying some homage to the active site. Along similar lines, the substrate that is acted on is ATP not DAG. DAG is a regulatory molecule, as far as I could tell. Also the paper would benefit from an understanding of whether the active or inactive form is “meant” to have the ATP substrate bound.

In the Results section titled “Molecular basis of PKC II autoinhibition and ….” suddenly there are two chains. I assume this is intended to refer to two chains in the asymmetric unit. However, this is completely up to the reader to surmise.

I’m not sure whether the authors intended to use the word “lipid” as shorthand for phospholipid or just the hydrophobic portion of a phospholipid. If the latter, then whenever there is “lipid” binding, near to this hydrophobic area there should be something on the protein that contends with the hydrophilic part.

The description of the differences between the different isoforms of the PKC were quite good. The work that went into clearly describing the difference paid off.

The text that is associated with Fig 2 and figure itself were confusing to me. The overlapping of the kinase domain and the placement of the N-terminal domains was specifically confusing. At some point in the text it says “two of them were comprised of similar molecular interactions”, then looking at the figure, there are three very different interactions. Further the colors have changed – keeping at least one of the N-terms in similar domain colors as Figure 1 would help with orientation.

Overall, a small bubble cartoon of the asymmetric units in the crystals would have been very helpful. This would have particularly helped in underscoring the challenges of the project and why much analysis was done.

The lipid-lever phraseology was confusing though the alliteration is appreciated. As far as I followed the proposed mechanism, and especially as I followed Fig 3a, a portion of the protein is sequestered into the phospholipid membrane. This process removes contacts that are made between domains in a non-membrane state. First, my complaint here involves the lipid vs phospholipid issue I raised earlier. The protein does not only need to prefer the hydrophobic section, but there must be something that deals with the strong phospholipid contacts. Second, this is more like a phase sequestration or membrane integration process. Would be good to note in Fig 3a whether the drawn lipids are “to scale” and if not, to make

some attempt to make it so.

The only substantive issue I have with the science, rather than way or specificity with which the information was conveyed, was the lack of a solution analysis. There appears to be some SAXS data available (though it appears to be collected both at the Advanced Photon Source and the Advanced Light Source – per text vs acknowledgement). Quite easily the full length solved structure (or a model where the missing residues are modelled in) could be put into a SAXS calculator like crysol or foxs and the result could be compared to the measured SAXS profile. If the match is poor and conformational change is suspected for the difference, the program BilboMD or EOM could be used to help define the conformational flexibility required to fit the data.

I apologize to the authors for being critical of the writing after they clearly have put so much effort into the science. Going through rounds of review is never fun. However, having made some effort in deciphering the text, I think the hardest part is done. With a bit of thoughtful revision (thinking about what the reader needs to know and will assume), the effort will come through much more clearly. The conceptual issues I describe were exemplary of distracting issues that prevented me from being able to read through the paper without having to research whether the authors meant one thing or another. I suggest going through the entire paper for issues like those raised here. I’m pretty sure there are more that I didn’t catch.

Version 1:

Reviewer comments:

Reviewer #2

(Remarks to the Author)
Major comments
• Supplementary Figure 3b. The authors include new data to support the C1b-PS interaction they observe in their crystal structures. Mutation of G24Q in the PS segment increases activity, rather modestly, at high concentrations of phosphatidylserine-containing micelles. However, I am concerned about both the interpretation and depiction of this data:
o The activating effect of the mutation is not seen in the absence of lipids, despite the authors’ claims that it destabilizes the inactive conformation. This weakens the authors’ claim that G24 is part of a physiologically relevant PS-C1b interface.
o The x-axis is labeled as % lipid content, which has no meaning. It is not clear what the authors mean, but the lipid content of these reactions should be expressed in standard concentration terms.
o It is not clear whether DAG was included in these micelles and, if so, at what concentration. Since phosphatidylserine (PS) binds to the C2 domain and not the C1b domain being investigated in this experiment, the presence of absence of DAG is important for interpretation of the result. In fact, as the authors state, the binding site in the C2 domain for PS is solvent-exposed, such that PS alone would not be expected to activate PKC.
o Finally, the experimental validation of the authors’ structure(s) is extremely important for an assessment of their physiological relevance. Such data are therefore an integral part of the authors’ narrative and should not be relegated to Supplementary Data.
• Figure 2d. The authors use limited proteolysis to test the effects of mutation of the C2-kinase domain interface in their structure. This experiment was complicated by the degradation of PKC lacking its PS segment, so the authors engineered a cleavable variant with which they could remove the PS segment post-purification. With the purified proteins in hand, they tested their susceptibility to elastase cleavage as a proxy for their stable compact intramolecular association. However, I do not agree with their interpretation of the results:
o Both PS-cleaved mutant proteins are already significantly degraded at t=0 in comparison to the non-cleaved proteins, presumably because the introduction of the Prescission cleavage site or cleavage itself made the proteins more susceptible to degradation by contaminating proteases.
o It is notable that the PS-cleaved proteins in Figure 2d look considerably less pure than their non-cleaved counterparts, so interpretation of the cleavage patterns over time should be treated cautiously.
o The elastase-mediated increase in the 40 kDa band corresponding to the kinase domain, which the authors use to assess stability of the proteins, is actually lower for both PS-cleaved mutants than their non-cleaved counterparts. If one accepts this, then this experiment does not support the authors’ conclusion that the Y285R mutation destabilizes the C2-kinase interface.
o This does not mean that the authors’ structure is not physiologically meaningful, it just means that the mutagenesis experiment presented in Figure 2d is not sufficient to validate the interface. As such, I would not support its publication, either as a main or supplementary figure panel.
• Figure 5. The authors have used an inconsistent coloring scheme that makes interpretation of their data unnecessarily complicated. For example, Fig. 5g uses black, red and magenta for the three samples, while Figure S7d,e use black, red and blue for the same samples. Although the PDFs in the rebuttal figure S7e do not correspond to the particle parameters listed in Figure 5g, the authors seem to have fixed this problem in the actual supplemental figure. (The rebuttal figure S7e mixed up PKCbI+ENDX and PKCbI+TAM? (the PKCbI+TAM curve clearly tends towards a lower Dmax than the other two curves, but, according to 5g, it should be PKCbI+ENDX that tends towards the lower Dmax).
• Figure 5, SAXS analysis. Although the employment of SAXS is ostensibly to look at the effects of ENDX and TAM on the compactness of PKCb, the authors miss an easy opportunity to compare their solution scattering profile to the high-resolution structure of autoinhibited PKC. Assuming that they included a non-hydrolyzable ATP analog in their PKCb sample, they could reasonably compare the theoretical solution scattering of their model for autoinhibited PKCb to their experimental data using CRYSOL. This would go a long way to helping substantiate the physiological relevance of their crystal structures, given the obvious impact of lattice contacts and domain swaps that they have alluded to themselves. This

requires no additional experimentation and a very simple data analysis. Reviewer #3 suggested the same thing, although the authors have argued against it. In my opinion, however, the authors' model for autoinhibited PKCb is complete enough to compare its solution scattering with their experimental data, with only ambiguities about the positioning of small linkers that will not influence the overall scattering curve to an appreciable degree at the resolution of SAXS.

Minor comments
• Pg 2, ln 32 and pg 3, ln 20. The authors claim, misleadingly, that no structure of a full-length PKC has been published to date. In fact, the structure of rat PKCbII, which the authors reference, is of the full-length protein, albeit with missing electron density for the PS and C1a domains.
• Pg 3, ln 37 and pg 10, ln 43. The authors should be careful when referencing PHLPP as a PKC modulator. It has recently been shown that PHLPP2 is a pseudophosphatase with no catalytic activity (Husremovic et al, PNAS 2025). The effects of NSC117079 are therefore likely not the consequence of PHLPP inhibition.

Reviewer #3

(Remarks to the Author)
The manuscript is easier to read and much clearer then the first draft. Hopefully, the effort will lead to higher citations.

Version 2:

Reviewer comments:

Reviewer #2

(Remarks to the Author)
Major comments

Figure 1g, ln 154-159. The authors have addressed my concerns with the presentation of this data, adding the molar percentages of the components in their micelles to the x-axis. Although the use of TWEEN20-based micelles in these assays is a strange choice, particularly since the final condition contains no TWEEN20, the comparison of wt and mutant suggests that G24Q indeed sensitizes PKCb to activation in the presence of physiological agonists.

Figure 2d. The authors have removed the problematic limited proteolysis experiments from the manuscript. I appreciate their efforts, but I think it is appropriate to acknowledge the deficiencies in the experiment.

Figure 5. The authors have fixed the inconsistent coloring scheme.

Figure S7g. Additional SAXS analysis. The authors now present evidence that their crystal structure of autoinhibited PKCb is a reasonable fit to its conformation in solution, as evidenced by its solution scattering (SAXS).

Minor comments
There is still a referencing error in the introduction: the authors claim, misleadingly, that no structure of a full-length PKC has been published to date. In fact, the structure of rat PKCbII, which the authors reference, is of the full-length protein, albeit with missing electron density for the PS and C1a domains. The authors have fixed the reference to this study in their discussion, but not in the introduction. This is a minor issue that is easily fixed and the authors are correct in their assertion that a complete mechanistic understanding has been elusive in the absence of structural information on the PS and C1a domains that were not visible in this structure, but it should be phrased as such.

The authors have addressed concerns that the effects of NCI117079 may not be mediated by inhibition of PHLPP, but the sentence that references this study in the discussion implies that the compound still influences PHLPP2 behavior, for which, if you take away inhibition of phosphatase activity, there is no evidence. The authors' findings rather imply that NCI117079 exerts effects on cells that are not mediated by PHLPP. Whilst this might be inconvenient to the authors' narrative on PKCb phosphorylation, I believe that this caveat needs to be appropriately acknowledged. Again, this is a minor issue, but it significantly affects the reader's interpretation of the authors' findings. This is not a criticism of the authors for having used the compound, since they were not aware of the new study when this work was carried out, but I urge them to them to be precise and transparent in reporting its findings.

Open Access This Peer Review File is licensed under a Creative Commons Attribution 4.0 International License, which permits use, sharing, adaptation, distribution and reproduction in any medium or format, as long as you give appropriate credit to the original author(s) and the source, provide a link to the Creative Commons license, and indicate if changes were made.
In cases where reviewers are anonymous, credit should be given to 'Anonymous Referee' and the source.
The images or other third party material in this Peer Review File are included in the article's Creative Commons license, unless indicated otherwise in a credit line to the material. If material is not included in the article's Creative Commons license and your intended use is not permitted by statutory regulation or exceeds the permitted use, you will need to obtain permission directly from the copyright holder.
To view a copy of this license, visit https://creativecommons.org/licenses/by/4.0/

REVIEWER COMMENTS

We sincerely thank the reviewers for their thoughtful and constructive feedback. In response, we have made substantial revisions to the manuscript, including new experimental data, expanded analyses, and improved descriptive language to enhance overall readability. PKC enzymes are remarkably (and wonderfully) complex, and uncovering their molecular mechanisms is a challenging yet rewarding endeavor—one we feel privileged to be able to contribute to. As requested by the reviewers, we have added more detailed background information and interpretive discussion, which has increased the manuscript's length by approximately 30%. We look forward to the reviewers' feedback on our revised submission.

Reviewer #1 (Remarks to the Author):

This paper features multiple structures of full-length PKCβI and II in active and inactive states. The structures are at good resolution and well-refined, and therefore highly credible. This is an important and long-awaited structural tour-de-force worthy of publication in a high profile venue. The discussion is clear and thorough. The functional aspects of the paper are much less impressive.

We thank the reviewer for their recognition of the strengths of the paper and thoughtful critique. We appreciate the comments of all reviewers and have addressed them as outlined below as well as in the revised manuscript, which we believe has substantially improved it and strengthened its findings.

A natural assessment of the structures would be to mutate the interface and explore the dose-response for translocation in cells or activation on liposomes. To some extent this type of information for the kinase-C2 interface is provided in Fig. 2c. It would be preferable for this type of experiment to be shown for the other interfaces. It would be much more informative to see dose-response instead of total activity at a single condition. The experiment in Fig. 3 does not show anything that wasn't known by the late 1980s, and the single mutant studied in Fig. 4c is not a very complete analysis. In the interests of getting these fascinating structures into the public view faster, perhaps such studies could be left for a follow-up paper.

The panel of mutations in Figure 2c includes a mutation in the C1b-kinase interface (A570E, depicted in panel 2b). Due to the smaller size of this interface, we could only design one mutation where all rotamers of the mutated residue would introduce a steric clash. The C1a and Kinase domains do not interact directly in the structure. As noted by the reviewer, Figure 2c includes data for activity of PKCβI at three different concentrations of mutant alongside the WT protein showing consistent patterns of reduced activity regardless of protein concentration in the presence of activating PS/DAG phospholipids.

Although the concept of phorbols requiring embedding in membranes to activate kinase activity depicted in panel 3b was previously known as noted by the reviewer, this exact experiment showing that PDBu cannot activate kinase activity *in vitro* even at extremely high concentrations has not (to our knowledge) been described. Therefore, we

chose to perform this experiment and include it in our paper alongside the model of activation to provide a specific biochemical context that demonstrates for the absolute requirement of a membrane in order to activate PKCβ.

We now include a comparison of alanine and valine mutants of F648 in triplicate as Supplementary Fig. 6g along with a quantification of cleavage efficiency. Although the alanine mutant is clearly different than WT, the valine mutant is more subtle and we note that the data generated by this technique is too noisy to see small effects. We attempted to generate mutants with larger residues at position 648, but the resulting proteins did not express well and were unstable. We hypothesize this is because the resulting mutants are too active and prone to dephosphorylation, deactivation, and degradation in the HEK293F cells. Nonetheless, these data demonstrate that changes in residue 648 can alter the conformation of PKCβ in a way that is generally consistent with the proposed model for PKCβI/II isoform differences.

Supplementary Figure 6g:

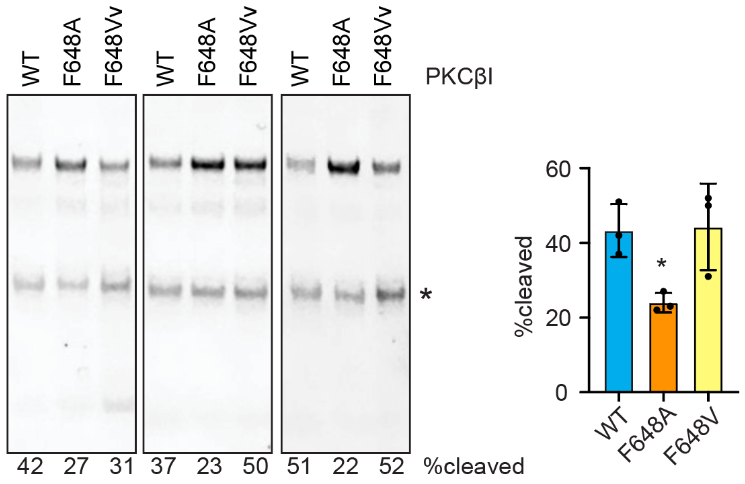

Introduction, line 5, and elsewhere, substitute "conventional" PKC for "canonical"

We have made this change throughout the manuscript.

Fig. S2 Legend. "Experimental" in reference to electron density syntheses is ambiguous. Specific the phasing model. Were the models shown including in the phasing of the corresponding maps, i.e., are these omit maps, or not? Fig. 2d should show electron density. An anomalous difference map would be desirable as well.

We have revised the legend for this figure panel to state that they are 2mFo-dFc maps. The comment referring to Fig. 2d appears to be for Supplementary Fig. 2d. The anomalous signal in the PKCβII dataset is quite low, owing to the weaker anomalous scattering of Zn at the wavelength used for data collection and the overall lower strength of diffraction from these crystals. Therefore, we now include 2mFo-dFc electron density

maps contoured at 4 sigma to reveal the high electron density around the Zn atoms as Supplementary Figures 2d and 6a to show the Zn sites in PKCβII and PKCβI respectively:

Supplementary Figure 2d:

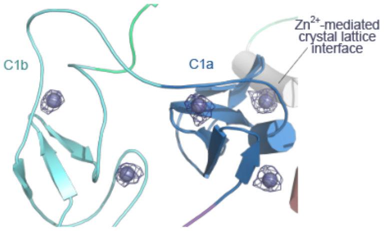

Supplementary Figure 6a:

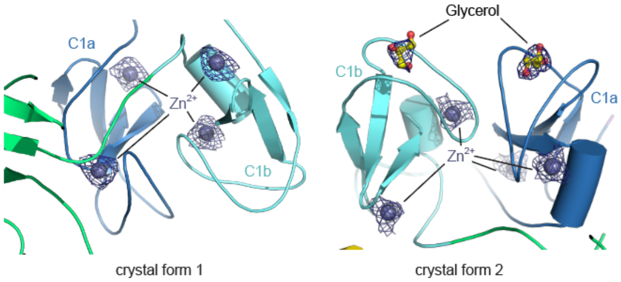

Ref. 21 should be to Violin et al. 2003.

We have corrected this reference.

Reviewer #2 (Remarks to the Author):

Summary

Protein Kinase C (PKC) is a family of 10 mammalian iso-enzymes that transduce the signal generated by phospholipase C (PLC) into appropriate downstream responses in the cell. PKCs have been implicated in a number of disease pathologies and regulate a diverse array of critical intracellular signaling pathways that make the study of their regulation incredibly valuable. Unfortunately, decades of study have failed to elucidate the complex regulatory mechanism(s) of these kinases at atomic resolution. The authors of this manuscript present a series of X-ray crystal structures of two splice variants of PKCb, which they use, together with a series of accompanying biochemical and cell biological experiments, to rationalize the mechanism of PKC activation by calcium, phospholipids, and diacylglycerol (DAG). In addition, the authors report the characterization of endoxifen, a metabolite of the anti-cancer drug tamoxifen, as an allosteric inhibitor of PKCb. The topic is of broad interest to a general audience, including basic, translational and clinical scientists. The study, however, falls short in a number of areas and, in the opinion of this reviewer, there are significant reasons to doubt some of the conclusions that the authors make on the basis of their data. In particular, serious

concerns are raised about the quality of some of the supporting biochemical and biophysical data, which the manuscript heavily depends on to support the authors' structure-based hypotheses.

We thank the reviewer for their recognition of the strengths of the paper and their thoughtful critique. We appreciate the comments of all reviewers and have addressed them as outlined below as well as in the revised manuscript, which we believe has substantially improved it and strengthened its findings. One note we will make for clarity for the editor and other reviewers is that this critique provided by Reviewer 2 is referring to their review of a previous submission of this manuscript at another journal, for which we were not provided the opportunity to respond. Of particular importance, we modified our manuscript based on the comments that we received prior to resubmitting it to Nature Communications.

Major Comments

***Structure of PKCbII reveals architecture of autoinhibited state**.*

The authors have determined the structure of full-length PKCbII in which 2 chains are observed in the ASU. The first chain (chain A, presumably) forms an intramolecular complex in which electron density is visible for 91% of the chain, while the second chain forms a domain-swapped interface between its regulatory domains and the kinase domain of an adjacent protomer in the crystal lattice. In this second instance, the inter-molecular interface is near-identical to the intramolecular interface observed in chain A. The structure represents the most complete picture of any PKC isoform to date.

The catalytic cleft of each kinase domain is engaged by a pseudosubstrate segment (PS) (either its own, or that of its domain swap partner), consistent with the known mode of substrate:kinase interaction. The DAG binding surface of the C1b domain is observed to be buried in intramolecular contacts with the PS and kinase domain, in contrast to a previously reported structure of PKCbII in which the C1b domain was observed to form an intramolecular contact with the N-lobe of the kinase domain (Leonard et al., Cell 2011), an interface that was validated with membrane translocation assays in cells both in the original manuscript and in a follow-up manuscript published by the same group (Lucic et al, JMB 2016).

In the original submission of this manuscript, it was difficult to assess the physiological relevance of the reported C1b-PS-kinase interface in the absence of any mutational data. The authors have added kinase activity data on a mutant, G24Q (Supplementary Figure 3D) to substantiate this interface. Although the data presented supports their claims, the data points have no error bars (presumably n=1) and the y-axis scale is '% kinase activity', where it appears that the authors have normalized their data to the signal from the highest wt concentration. At the very least, this experiment needs to have sufficient replicates and an appropriate activity scale.

We have edited the axis label and repeated this experiment to include 3 replicates. Additionally, we have expanded this analysis to show that G24Q is also more active in micelles that contains lower concentrations of phosphatidylserine/DAG, consistent with the proposed effect of this mutation being to destabilize the inactive conformation. We have replaced the data in Supplementary Fig. 3d with the following (now Supplementary Fig. 3b) which includes error bars and statistical significance testing:

Supplementary Figure 3b:

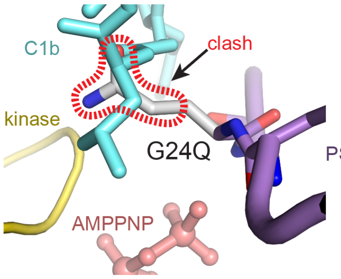

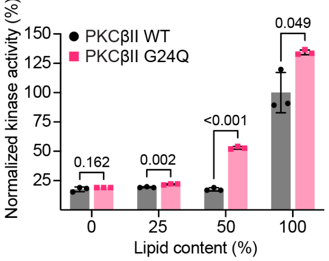

The DAG-binding surface of the C1a domain is observed to be solvent accessible. The observation that F43 of the C1a domain is buried in an intramolecular interface with the kinase domain is consistent with a previously published report showing that its mutation results in accelerated membrane translocation of all conventional and novel PKCs (Lučić et al, JMB, 2016).

To emphasize this point, the DAG binding site of the C1a domain is solvent accessible, but a portion of the membrane-binding surface of the C1a containing F43 is sequestered at the interface of the kinase domain in a manner that is mutually exclusive with interaction with a phospholipid membrane. We have added Supplementary Figs. 3c,d and edited the main text to help illustrate this point for the reader

Revised text:
"Our structures also revealed that the C1a domain can form a hydrophobic interaction and salt bridges with the kinase domain and the C-terminal extension (Fig. 1f, Supplementary Fig. 3c). The DAG-binding site on C1a was solvent-accessible, but a small portion of the surrounding hydrophobic residues that would be expected to interact with the hydrophobic lipid core, including residue F43, was sequestered at the C1a-Kinase interface, an interaction that would be mutually exclusive with membrane-binding (Supplementary Fig. 3d). This configuration is supported by previously reported site-directed mutagenesis data showing that F43A, D382K, or E655K mutations that would disrupt the observed C1a-Kinase interface resulting in a more extended conformation and increased phospholipid interactions[17,45] (Supplementary Fig. 3e)."

Supplementary Figure 3c and 3d:

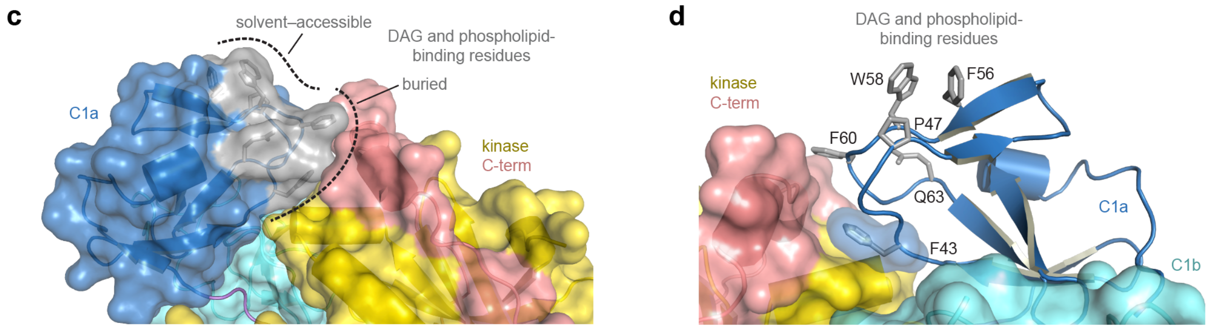

Stabilization of the active conformation of the kinase domain and its intramolecular interactions by phosphorylation of T500, T641 and S660, however, was already known, including how mutation of these sites to non-phosphorylatable alanines causes PKC to partition into the insoluble or membrane-bound fraction.

It has been previously shown that the three phosphorylated residues form interactions within the kinase domain that would support the folded, active kinase (Leonard et al 2011, Grodsky and Love 2006), and that without phosphorylation, PKC partitions into the insoluble or membrane-bound fraction. However, the molecular basis by which phosphorylated residues regulate the lipid affinity of distal domains (C1 & C2) through interdomain interactions was not known, and is now revealed by the structures in this report. Interactions between the pseudosubstrate and pT500 support sequestration of the C1a against the kinase domain, while the lipid binding surface of the C1b is sequestered at the kinase domain in part through interactions with pT641.

Crystal structures of PKCbI reveal active and inactive states.

PKCbI and PKCbII are slice-variants that differ only in the C-terminal ~50 amino acid tail of their kinase domains. The authors report two more crystal structures, this time of PKCbI in two different crystal forms.

Crystal form 1 contains 2 mol/ASU in which one of the two chains is observed to have electron density for the PS in the catalytic cleft of the adjacent protomer, again via a domain swap. The arrangement of the regulatory domains in this crystal form seems to be incompatible with the inactive conformation that they report for PKCbII, and the authors investigate this arrangement later in the context of PKCbI activation.

Crystal form 2 contains 1 mol/ASU in which the PS segment is not observed bound to the catalytic cleft, despite it being unobstructed and available for engagement. The authors suggest that the packing of the regulatory domains with the kinase domain is similar to that observed in PKCbII, but this is difficult to assess based on the data presented.

For clarity, we note that we are referring to crystal form 1 (not 2) when describing the interaction between the N-terminal domains of chain A that interact with the catalytic domain of chain B through a domain swap. We have edited Supplementary Fig. 5b to better depict this.

Supplementary Figure 5b:

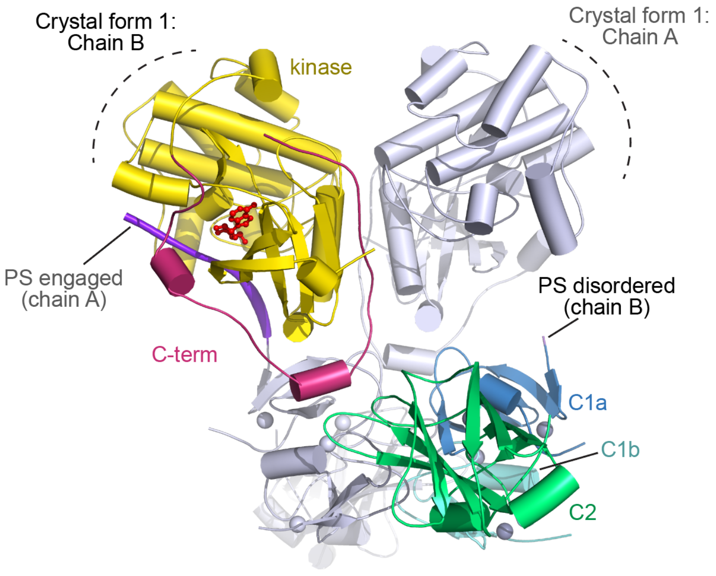

PKCβI crystal form 1 asymmetric unit

We have additionally expanded the writing to include more details on the process in determining the functional interdomain interactions of PKCβI based on the two crystal forms. In short, we first visualized the symmetry related N-terminal domains in the immediate vicinity (within 4Å) of the kinase domains of interest (2 copies from crystal form 1 and 1 copy from crystal form 2), and aligned these structures by the kinase domain. We then looked for interactions between kinase and N-terminal domains that are consistent between the three structures, and identified two that consisted of similar interacting residuues in all three crystal lattices (labelled N-term 1 and 2). The interaction between N-terminal 2 and the kinase domain contains a C1a and PS interaction similar to the structure of inactive PKCβII. The similarity here only extends to the fact that the PS is bound to the substrate-binding pocket on the kinase domain followed by the C1a domain forming an interface with the kinase domain. For this reason, we conclude that this interaction represents the auto-inhibited conformation of PKCβI. Specific structural details on how the auto-inhibited conformations of these two isoforms compare are explored in a later section of the paper titled "***Molecular basis of isoform-specific differences between the splice variants PKCβI and PKCβII***"

Visually, these structures do not appear to be that similar and the authors themselves state that the C1b domain occupies "an altered location…that we attribute to the autoinhibited conformation for the PKCbI enzyme." It is not clear what the authors mean by this, but it is difficult to imagine different autoinhibited conformations for PKCbI and PKCbII given that they are identical except for their C-terminal tails.

We appreciated this comment. To better clarify, we have expanded our initial description to include that this likely involves an equilibrium between the C1b being docked on the PS as depicted in PKCβII, vs it being displaced and not engaged in inhibiting the kinase domain as depicted in Fig. 4b. Therefore, the size of the residue at 648 (B1 numbering) and the resulting shift in position of helix αB alters the C1b docked/displaced equilibrium, with larger residues favoring displacement of the C1b resulting in increased protease sensitivity of PKCβI for larger 648 residues. See also response to comment below regarding residue 648 mutants. Beyond the C1b domain differences, our model proposes that the PS-C1a-kinase domain interfaces are very similar in PKCβI and II. We have added the following text, an addition to other changes:

"These data are consistent with a model whereby a smaller residue at position 648 favours a conformation with the C1b docked on the PS as seen in the PKCβII structure, while larger residues favour an equilibrium state with a displaced C1b domain."

Here, a definitive analysis of their claims is somewhat impeded by a more quantitative comparison of the PKCbII and PKCbI (crystal form 2) structures. Given that the PS segment is not observed in the catalytic cleft and the C1b domain occupies a different position, it should at least be considered that the arrangement of the domains could be the consequence of lattice packing interactions that are energetically more favorable.

We thank the reviewer for this comment, and we have now included commentary on this possibility in the discussion section of our revised manuscript:

"Here we present the most complete structural analysis of any PKC reported to date. We have determined a series of PKCβI and βII structures that contain all of the structured domains. We have identified interdomain interactions that comprise specific states of activity of the PKCβI/II enzymes that are supported by existing data or generated additional biochemical experiments to test and validate our molecular models, although the possibility remains that the domain arrangements could be the consequence of crystal lattice packing. The models obtained from these PKCβI/II structures illuminate the conformation of key stages in the allosteric regulatory cycle of PKC enzymes, which we demonstrate is mediated by rearrangement of the regulatory N-terminal domains…"

The authors observe an intriguing interface between the regulatory domains of PKC and its kinase domain that is common to both PKCbI structures that they have determined. In this conformation, which the authors refer to confusingly as N-term 1, the C1a, C1b and C2 domains pack against the C-lobe of the kinase domain. They make an interesting comparison to the AF2 prediction of PKCb, which places the domains in a similar arrangement. The authors have added in the AF2 prediction for the PKCa isoform, but there are still no PAE plots included in Supplementary Figure 5e, which would offer a metric-based assessment of the confidence of the predictions. Nevertheless, prediction of the same arrangement for other PKCs and the strong similarity to their experimentally determined structure strengthens the authors' claims.

We have now added PAE plots to Supplementary Fig. 5e.

<u>Supplementary Figure 5e:</u>

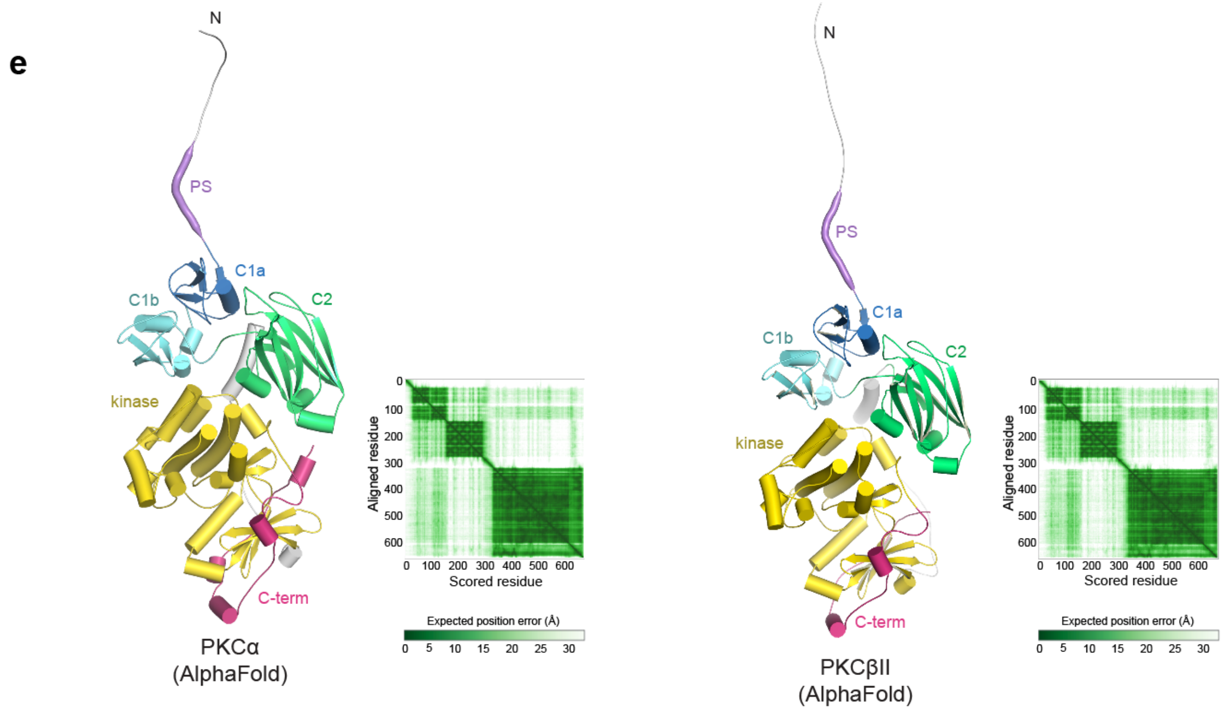

PKCbI active conformation

The authors next investigate the interface between the N-terminal domains and the C-lobe of the kinase that they observed in both crystal forms and that is also predicted by AF2. Given that the AF2 predictions of PKCb and PKCa are convergent with the authors' experimentally determined arrangement of the regulatory domains, the implications are intriguing. Namely, that the membrane binding surfaces of the C1a, C1b, and C2 domains are observed in the same plane and binding of the PS segment to the kinase domain would be impossible. Should this structure represent the active conformation of PKCbI, it would help resolve two important questions regarding PKC activation: (1) does PKC form a defined, active conformation on the membrane (implications for substrate specificity etc) and (2) what prevents the PS segment from inhibiting activity on the membrane?

The authors attempt to validate this interface with a site-directed mutagenesis approach coupled to an in vitro FRET-based kinase assay (why such a complicated kinase assay readout is required in vitro is not clear – other, more classical in vitro assays would almost certainly provide a cleaner readout). They report a series of mutants in the observed C1b-kinase and C2-kinase interfaces that impair PKC activity in vitro. However, the effect is modest for all mutants, except for F286E, which (strangely) seems to exhibit no concentration-dependent change in activity (Figure 2C).

Here we used the CKAR substrate for our *in* vitro kinase assay as it allowed real-time monitoring of kinase activity by direct changes in the substrate itself in a controlled buffer system. Classical *in vitro* assays rely on 32-P ATP, however, these assays require additional steps after a kinase reaction to readout activity and thus offer only endpoint assessment of activity. Further, (and unfortunately) due to institutional requirements we are not equipped to work with 32P in our laboratory.

The activity of the F286E mutant is just above background levels, and because of this a dose-dependent increase is difficult to detect. Nonetheless, this does indicate that F286E impairs activity of PKCB. The modest effect of the other mutants is attributed to destabilizing the active conformation described in 3a. We note that each mutation only destabilizes a small portion of the interface in the active conformation, which is also stabilized through interaction with the phospholipid membrane, and thus the modest effect of the other mutations is not necessarily unexpected.

Additionally, the observed packing arrangement of the domains exclusively involves the distal surface of the C-lobe of the kinase domain, which is not a reported surface of allosteric regulation for PKC. In fact, there are no movable elements in that surface that could regulate kinase activity allosterically. It is therefore hard to reconcile changes in activity in vitro caused by mutations that would disrupt these interfaces. The authors do not discuss this apparent contradiction, although, as previously stated, the interface they observe is potentially relevant. The question therefore arises: what is the interface useful for? In this respect, I believe that the authors may want to consider more explicitly the implications of the interface for the conformation and substrate specificity of PKC on the membrane. The consequences of such an interface for maintaining active and very highly specific PKC on the membrane due to a well-ordered conformation are quite profound, in my opinion, and are possibly the most interesting findings of this study.

We thank this reviewer for bringing this point to our attention. In this case the statement about allosteric rearrangements refers to inter-domain rearrangement, rather than through triggering intra-domain conformational changes within the kinase domain itself. Thus, the implications of the active conformation that we propose would be to stably sequester the N-terminal regulatory domains distal from the active-site or intra-kinase allosteric regulatory surfaces, independent of allostery within the kinase domain. This model is supported by data (i.e. ref 26: Grodsky et al. 2006) showing that the activity of truncated PKC lacking the regulatory domains is equivalent to that of phospholipid-membrane bound WT PKC.

We now include the following in the manuscript to convey this point:

"The C1b and C2 domains only interact with the kinase domain C-lobe on non-moveable surfaces, which suggests that this mode of binding would not alter the kinase domain N- and C-lobes movement between open and closed conformations that are part of the normal catalytic cycle of substrate engagement and release."

We have now expanded our description of the active form to (better) convey three significant implications of the proposed active conformation: 1) it anchors the PS and

other regulatory domains where they cannot re-engage and occlude the active site. 2) The active conformation consists of interfaces between the C1a, C1b, C2, and surfaces of the kinase domain that are distal from the active-site, and these would form cooperatively with DAG (or phorbol) and Ca^{2+}-driven binding to the plasma membrane (mentioned already, but now more explicitly stated). 3) this particular binding conformation positions the active site a specific distance (measured as approximately 40Å in crystal form 2) away from the plasma membrane, suggesting this could be the prime cellular region for proteins to be located (at least at the time of phosphorylation) relative to the plasma membrane.
We now include the following in the manuscript to convey these points:

"A prominent feature of this domain arrangement is that the phospholipid-binding surfaces of the C1a, C1b, and C2 domains align in the same plane, as would be the case when they bind or embed in a plasma membrane. This suggests that this conformation would consist of cooperative interactions with the C1a, C1b, C2 domain, as well as the phospholipid membrane that could compete with the auto-inhibited state. We note that this arrangement would position the active-site of PKC approximately 40Å away from the plasma membrane, suggesting this could be the prime cellular region for PKC substrates to be located for phosphorylation. Furthermore, the PS would be anchored ~70Å away from the active site and prevented from re-engaging the active site in this conformation…"

I do not, therefore, dismiss a physiological relevance of the interface observed. Since the autoinhibited conformation of PKCb relies on the clamping of the PS segment by the C1b domain (Figure 1), a possible way to test this interface would be to examine the interface mutants in the context of a construct of PKCb lacking its PS segment. Kinase activity assays are unlikely to reveal allosteric changes for the aforementioned reasons, but the particle might reasonably be expected to remain compact and become extended upon disruption of the interface. This could be tested, for example, by SAXS analysis of PKCb interface mutants compared to PKCb, assuming that the recombinant proteins behave sufficiently well to make them amenable to such analyses.

We thank the reviewer for this thoughtful suggestion. In our present HEK293F expression system we are unable to obtain soluble ΔPS PKC, similar to a report from the Newton lab (Baffi et al. Mol Cell 2019) due to downregulation and degradation of constitutively active PKC. Therefore, we engineered a new construct that contained a Prescission protease sequence (LEVLFQGP) inserted between the PS and C1a domains of PKCβI, so that it could be expressed in human cells and adopt the correct autoinhibited state. We were able to express and isolate soluble protein, then remove the PS by cleavage with Prescission protease cleavage during purification. This yielded protein that was active, but (perhaps unsurprisingly) prone to degradation.

We then generated a mutant that combined one of the interface mutants (Y285R, Fig. 2b,c) with the PS-prescission mutant. Following prescission protease cleavage during purification, we were unable to obtain PS-prescission mutant proteins in sufficient quantity and purity for SAXS, so we instead used limited proteolysis to probe their structure. The premise for such an analysis would be that the removal of the PS would destabilize the inactive conformation, and a C2-kinase interface mutation would

destabilize the proposed active/membrane bound conformation. If this premise were correct, these two mutations (i.e. a double mutant) would be expected to yield a protein without either stable, ordered domain arrangement and thus be more susceptible to cleavage by proteases than either of the single-mutation proteins. We have performed this experiment and now include it as Fig. 2d: The methods, results and discussion sections have also been modified in light of this new data. The new results are shown below:

"We hypothesized the active conformation would be in competition with the inactive conformation, and that any dynamic intermediate state would be transient and short-lived. We generated PKCβI mutants where the inactive state was destabilized by removal of the PS domain (ΔPS), the active state was destabilized (Y285R), or both states destabilized by a double mutation (ΔPS/Y285R). PKC proteins that lack the PS are constitutively active and degraded in human cells[41], so we engineered a PKCβI mutant containing an insertion of the cleavage site for the site-specific protease Prescission to allow removal of the PS from PKCβI during protein purification to yield ΔPS PKCβI. We then probed the solution conformation of the PKCβI proteins using limited proteolysis with elastase (Fig. 2c). The most prominent cleavage product in WT PKCβI is approximately 40 kDa, suggesting it results from cleavage at a protease sensitive site between the C2 and kinase domain[53] (Supplementary Fig. S1a). Cleavage at the same site is lower for the Y285R protein suggesting the protein exists in, or spends more time in a more stable conformation. The ΔPS protein already contains a 40 kDa fragment that could not be resolved during protein purification, and the amounts of this fragment increased with addition of elastase, again consistent with cleavage at the same elastase-sensitive site. Strikingly, the inclusion of the Y285R mutant in the ΔPS protein increased cleavage by elastase at this site and enhanced the intensity of bands below 40 kDa, suggesting that in contrast to the full-length protein, the Y285R mutation leads to a less stable conformation. Collectively, these data suggest a model where the active conformation of PKCβI is destabilized by Y285R and in competition with the auto-inhibited state, while the double mutant ΔPS/Y385R destabilizes both conformations."

Figure 2d:

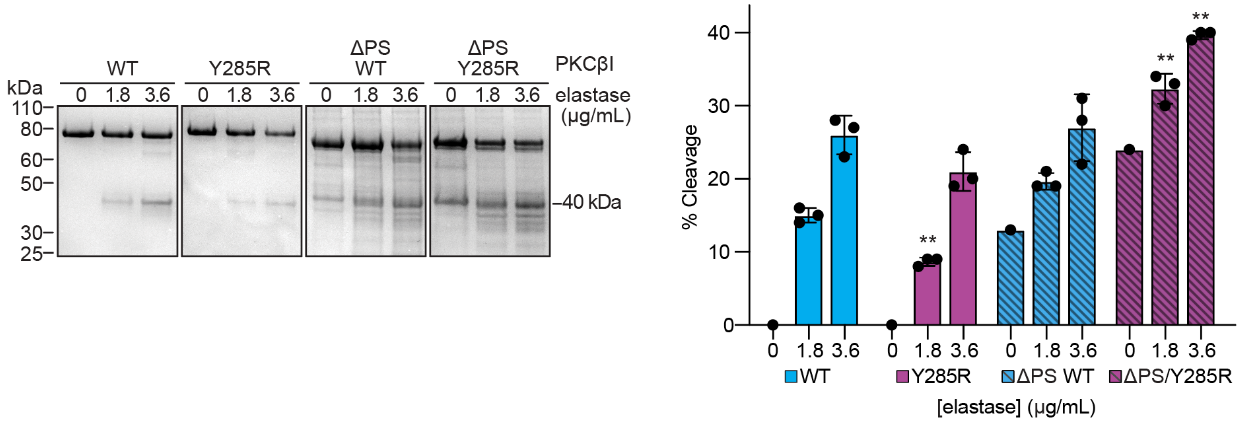

The lipid lever mechanism of PKC activation

Having determined that the above conformation is, according to their mutagenesis data, biochemically relevant, the authors sought to rationalize the activation of PKC on membranes containing DAG. DAG-mimetics, such as phorbol esters, are commonly used to activate PKCs in vitro. However, as the authors show, they rely on being embedded in a membrane for their ability to activate PKC (Figure 3B). The authors assert that, due to solvent accessibility of the lipid binding surface of the C1a domain in their crystal structure, phorbol dibutyrate (PDBu; a DAG-mimetic) binding would not disrupt the inactive conformation and therefore a direct competition mechanism can be ruled out. I strongly disagree with this statement for the following reasons:

1. The PDBu-binding surface of the C1B domain in their structure is buried in an intramolecular interface, which means that PDBu would compete for this interface.

The C1b domain PDBu binding site is indeed sequestered as stated by the reviewer, suggesting that PDBu would compete for this interface. However, the C1a PDBu/DAG binding site is solvent exposed in the inactive conformation (Supplementary Fig 3C,D and 6D), and PDBu binding would not be competitive with the C1a-kinase.

Supplementary Figure 3C,D:

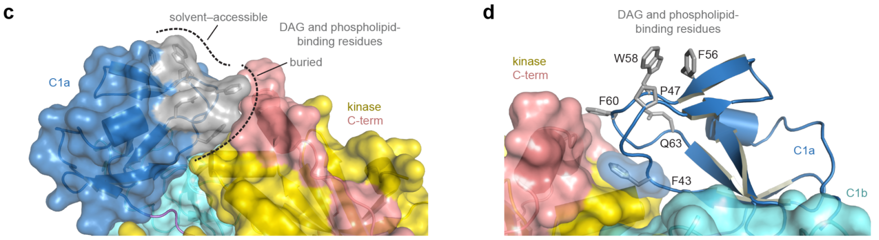

<u>Supplementary Figure 6D:</u>

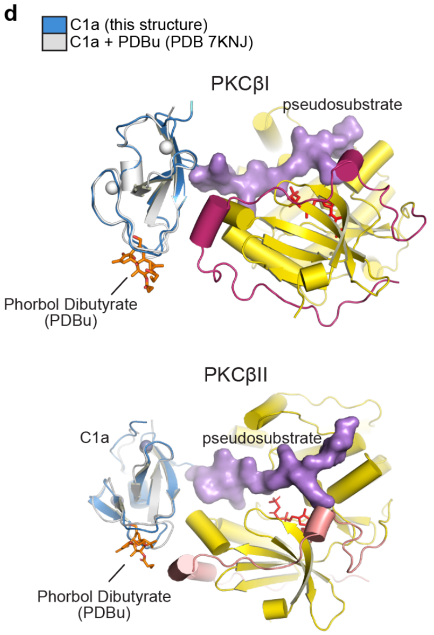

2. Multiple reports have unambiguously determined that the DAG-binding surfaces of the C1 domains (minimally one, but possibly both) are sequestered in the intramolecular, autoinhibited conformation. In fact, the naked C1 domains translocate to the plasma membrane with dramatically accelerated kinetics compared to full-length PKCs (Oancea & Meyer, Cell 1998; Lucic et al, JMB 2016).

The inactive conformation depicted in the PKCβII structure would indicate that the DAG-binding surface of the C1b is sequestered while that of the C1a is not, consistent with the second possibility listed. More precisely, although the C1a DAG pocket is solvent-accessible, many of the surrounding hydrophobic membrane-binding residues are sequestered at the C1a-kinase domain interface and therefore inaccessible for membrane intersection in the inactive PKC conformation. Therefore, the distinct difference in membrane recruitment kinetics of naked C1 vs full-length PKC (as described by Oancea & Meyer, Cell 1998; Lucic et al, JMB 2016) can be attributed to the sequestration of important residues in the inactive PKCβ in a conformation where they are unavailable for membrane localization.

3. Mutation of F43 (or its equivalent) in the C1a domain essentially unlocks all novel and conventional PKCs such that they translocate with near-identical kinetics to the isolated C1a-C1b domains. While an allosteric effect on the neighboring C1b domain and PS cannot be excluded, this observation strongly suggests that the DAG-binding surface of the C1a domain is also sequestered in the autoinhibited conformation. This raises the question of whether the interface observed in the authors' crystal structure is representative of the physiological conformation. Given the reported promiscuity of domain swaps in PKCb crystal lattice formation, this cannot be ignored.

There is an important distinction between the DAG/PDBu binding pocket located in the interior of the C1 domains, and the "outer" residues (including F43) that would interact with the hydrophobic phospholipid environment surrounding the DAG that can be made. While the inner DAG-binding pocket residues are solvent accessible in the C1a domain, the outer residues along one side of the C1a domain, including F43 are sequestered at the interface with the kinase domain. Both the inner DAG pocket and outer lipid biding residues need to be able to interact with the hydrophobic region of the phospholipids to bind to DAG/Phorbols when they are embedded in a membrane. Thus, although the PDBu/DAG binding site remains solvent accessible, sequestering F43 at the kinase domain interface is sufficient to occlude membrane interaction. We have added a schematic diagram (Supplementary Fig. 3e) to describe this model.

Supplementary Figure 3e:

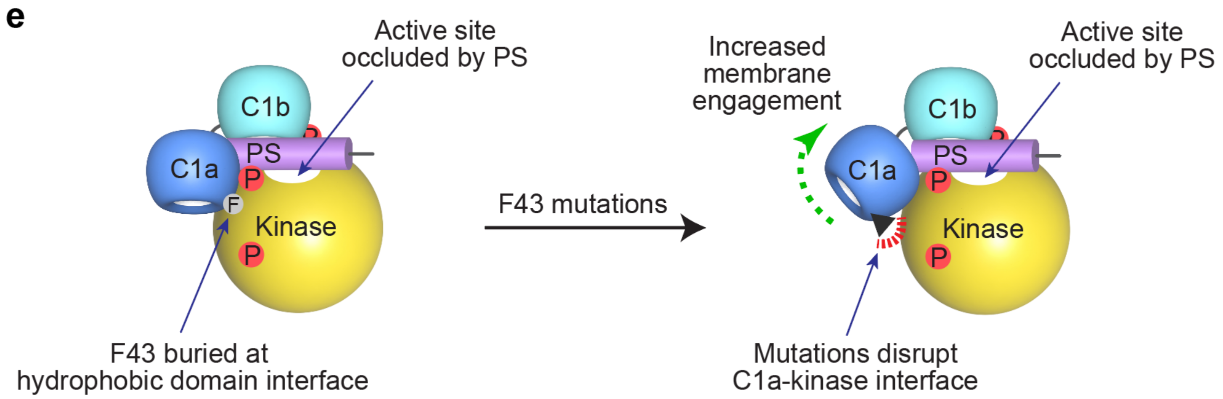

The authors use a limited proteolysis assay to probe PKCbI conformation in the presence of Ca2+, PDBu or lipids, and combinations thereof. They show, in Figure 3C, that DAG-containing lipid micelles result in increased elastase-mediate cleavage of PKCbII (presumably in the C2-kinase domain linker?), whereas PDBu and Ca2+ are insufficient. This supports the requirement for DAG/PDBu to be embedded in a membrane/micelle, but it does not rule out a competition mechanism between DAG/PDBu and intramolecular contacts of the C1 domains with the kinase domain.

Here the limited proteolysis assay does not detect any changes in the conformation of PKCβI induced by free PDBu, insofar as they can be detected by changes in accessibility of elastase cleavage sites. While we cannot rule out the possibility of altered interdomain contacts based on limited proteolysis alone, in combination with the data in panel 3a showing that PDBu does not activate kinase activity, the most probable interpretation is that free PDBu is not causing structural changes in PKC. We have modified the text to add this caveat:

"We used limited proteolysis to probe the conformation of PKCβI and PKCβII in the presence of Ca^{2+} and PDBu and observed that PDBu in solution did not alter elastase cleavage even at 100 μM (Fig. 3b), indicating that phorbol binding alone is also insufficient to drive a conformational change in PKCβI and PKCβII in regions that contain a elastase-accessible cleavage site. In comparison, the addition of micelles that contain DAG embedded within the phospholipid bilayer robustly stimulate kinase activity, and lead to increased elastase-mediated cleavage, consistent with a conformational change."

Minor comments related to Fig. 3C:
• Why do the authors compare PDBu with DAG-containing lipid micelles? This is a bit like comparing apples with oranges.

The data in figure 3C was included to demonstrate that PDBu alone cannot drive a conformational change in PKCβ, and thus the focus is on the PDBu data. Although we agree that DAG-micelles are different than PDBu, they were included as a positive control (the same micelles that are used in kinase assays throughout this study) to demonstrate

the effect of increased sensitivity to protease that could be expected in the case where PDBu alone *would* be sufficient to alter PKCβ conformation. We have revised the manuscript text to clarify the rationale for including the comparison to DAG/micelles:

"We used limited proteolysis to probe the conformation of PKCβI and PKCβII in the presence of Ca^{2+} and PDBu and observed that PDBu in solution did not alter elastase cleavage even at 100 μM (Fig. 3b), indicating that phorbol binding alone is also insufficient to drive a conformational change in PKCβI and PKCβII in regions that contain a elastase-accessible cleavage site. In comparison, the addition of micelles that contain DAG embedded within the phospholipid bilayer robustly stimulate kinase activity, and lead to increased elastase-mediated cleavage, consistent with a conformational change."

Minor comments regarding the text for this section:

The authors propose what they call a 'lipid lever model', in which they suggest that the membrane acts as a lever to pry the regulatory domains apart from the kinase domain. The model is based on the incompatibility of the domain arrangement observed in the autoinhibited structure of PKCbII with membrane binding by the C1a domain, which would cause a steric clash between the kinase domain and the membrane. Whilst the phrasing of this has been improved from the previous version of this manuscript, I urge the authors to reconsider their terminology here. If the C1a domain cannot approach the membrane because a of a clash between the membrane and the kinase domain, then binding is prohibited until the two domains dissociate (as the authors themselves write). As such, this model is not a 'lipid lever' model, which implies a force, but rather a process of conformational selection.

We have further revised this statement to highlight that it would proceed via a mechanism of conformational selection and not by force, or a process that requires energy expenditure. However, as the net result remains separation of the regulatory domains from the kinase domain, and since the presence of a membrane encourages the "selection" of this open conformation, we prefer to keep this term to describe the process. We feel that the "lipid lever" mechanism allows one to more easily visualize how the protein changes confirmation upon membrane engagement as has been revealed by our novel crystal structures, even though the molecular process is complex.

The text of this section has been revised to state:

"The C1a and PS domains would need to first undergo the slow process of dissociating[47] and embedding in the membrane, which would prevent rapid reassociation with the kinase domain. In this way, the lipid membrane effectively serves as a lever that holds the PS domain away from the catalytic domain, with the net effect of separating the PS and C1a domains from the kinase domain via a mechanism that we have termed a "lipid-lever"."

Although the authors use previously published membrane translocation data to support their lipid lever model, this is also problematic. In fact, published mutational data on F43, which appears to completely unlock the autoinhibited conformation of PKC (Lucic et al, JBC 2016), supports a conformational selection model in which the C1a and C1b domains must first dissociate from the kinase domain before they can bind the membrane. Here, whilst the authors have improved their terminology, their continued use of the 'lipid lever' terminology is misleading and their citation of references 14 and 17 needs to be more explicit. In particular:

1. It's not clear why reference 14 is cited after the sentence: "Additional data that supports the lipid-lever model comes from reports that the C1 domains exhibit enhanced membrane binding in the absence of the properly folded kinase domain 14, 17." Better references for this statement are the original studies by Oancea and Meyer (JBC 1998, Cell 1998).

We thank the reviewer for pointing this out. We have replaced citation of reference 14 with Oancea and Meyer for this statement.

2. The authors confusingly refer to the "absence of the properly folded kinase domain" in these studies, but what they mean is simply the absence of the kinase domain. They inadvertently imply that the proteins under investigation are not properly folded, which is not the case.

We apologize for the vagueness of this statement. "Properly folded" was intended to be inclusive of de-phosphorylated PKC. However, to clarify this statement we have changed it to now read: "<u>in the absence of the kinase domain</u>"

3. Reference 17 specifically deals with F43 and the equivalent residue in all conventional and novel PKCs. Since its mutation demonstrates accelerated membrane translocation of all cPKCs and nPKCs and the authors focus on it as part of their interface, it seems worthy of a more explicit explanation. In fact, this would help the authors to support their claims regarding the physiological relevance of the C1a-kinase interface they observe.

See also the response to the previous comments regarding F43. We have modified the text to expand on the discussion of F43 and its interface and added new figures depicting the structure around F43 (Supplementary Fig. 3f and 3g) and a new cartoon diagram (Supplementary Fig. 3e) depicting its role. We expect that these new additions to our manuscript sufficiently clarify the physiological relevance of the observed interface.

Revised text:
<u>"The DAG-binding site on C1a was solvent-accessible, but a small portion of the surrounding hydrophobic residues that would be expected to interact with the hydrophobic lipid core, including residue F43, was sequestered at the C1a-Kinase interface, an interaction that would be mutually exclusive with membrane-binding (Supplementary Fig. 3d). This configuration is supported by previously reported site-directed mutagenesis data showing that F43A, D382K, or E655K mutations that would disrupt the observed C1a-</u>

Kinase interface resulting in a more extended conformation and increased phospholipid interactions17,47 (Supplemental Fig. 3e)”

Supplementary Figures 3c,d,e:

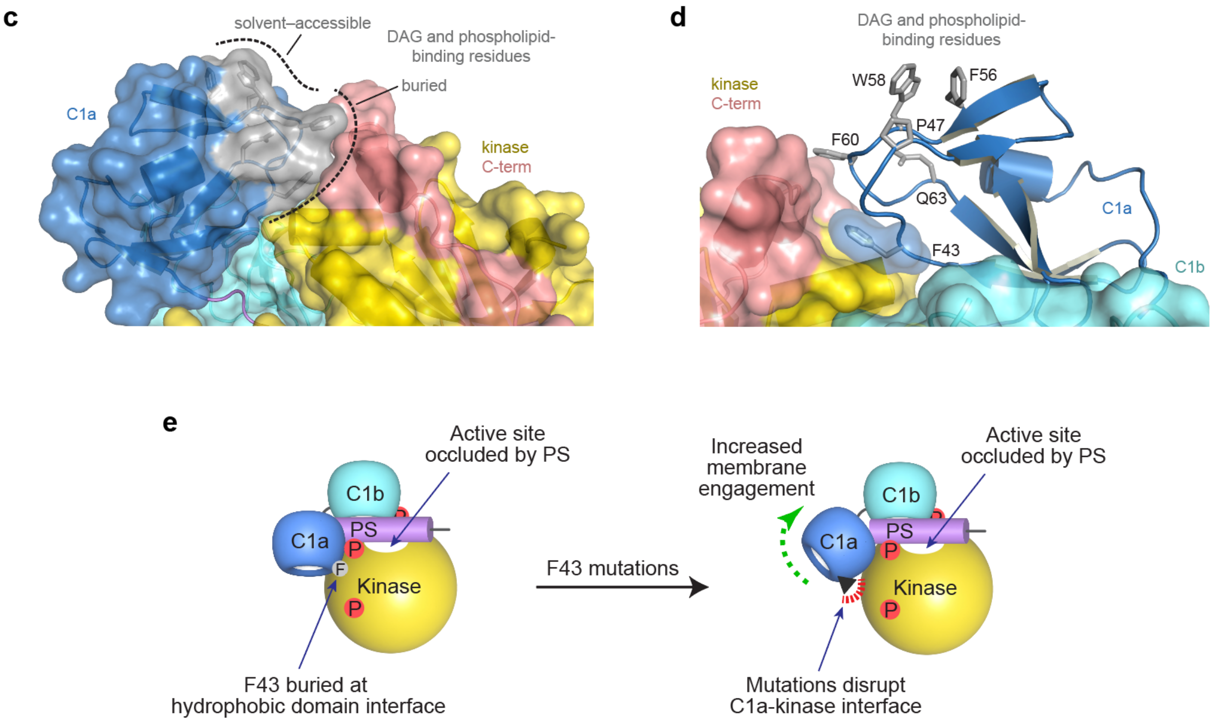

Molecular basis of isoform-specific differences between the splice variants PKCbI and PKCbII

PKCbI and PKCbII have been reported to have different affinities for phospholipid membranes. To try to rationalize this difference, the authors compared their structures of autoinhibited PKCbII (Figure 1) and PKCbI (crystal form I). The two structures differ in the following ways:

1. Electron density for the PS is only observed in the catalytic cleft of the neighboring protomer in the crystal lattice of PKCbI, an interaction brought about by a domain swap.
2. The C1b domain exhibits a different position: in PKCbII it is sequestered in an extensive contact with the catalytic domain that makes its lipid binding surface solvent inaccessible; in contrast, its lipid binding surface is solvent exposed in PKCbI.

According to the structure depicted in Figure 1D, the change in C1b position in PKCbI could reasonably be attributed to the absence of the PS in the catalytic cleft of one molecule in the ASU, an interaction which seems to be necessary for the intramolecular contacts of the C1b domain observed in PKCbII (Figure 1D). However, the authors do not appear to have considered this possibility.

We note that the altered position of the C1b domain in PKCβI is described for Chain A, for which the PS is present within the active site of the kinase domain (Fig. 4), via a domain swap (Supplementary Fig. 5A,B).

Instead, the authors focus on new contacts that they observe in PKCbI crystal form 1 between the C1b domain and the V5 extension to the kinase domain, which differs between PKCbI and bII. They postulate that the presence of F648 in PKCbI, which is V647 in bII, forces helix aB closer to the PS segment, thereby preventing an equivalent interaction of the C1b domain to that seen in PKCbII. To support this hypothesis, the authors engineered a F648A mutant of PKCbI and compared its sensitivity to elastase (as a probe for the active conformation) in a limited proteolysis assay. PKCbI was observed to be more sensitive to elastase cleavage than PKCbII in the presence of lipids. The following issues preclude a definitive assessment:

• It is unclear why the authors make a F648A mutant rather than F648V, which would convert F648 into the equivalent residue in PKCbII. As a test for the difference, this would seem to be a more appropriate comparison.

Data obtained using the F648A mutation provides a clearer phenotype and was chosen for the original submission. We now include data with F648V as Supplementary Fig. 6G, which shows an intermediate phenotype between that of F648A and WT, although the data obtained from limited proteolysis has variability that precludes the demonstration of a clear effect of this mutation.

• n=1 for the data shown in Figure 4C. There is no quantifiable difference in elastase sensitivity in the absence of lipids (middle panel), while each protein measured in panel C is performed only once. What has been quantified by densitometry is also not described in the figure legend and the effect size is modest at best (6% of PKCbII cleaved vs 11% of PKCbI and 3% of the F648A mutant). Since this lacks replicates and corresponding error bars, it does not provide compelling evidence to support the authors' conclusions.
• This figure/data has not changed from the original manuscript.

We now supplement the data from Fig. 4C with new data that includes F648V (Supplementary Fig. 6g) with 3 replicates. The % cleavage is defined as (cleaved band intensity/cleaved + uncleaved intensity), and we have added these details to the methods.

Supplementary Fig. 6g:

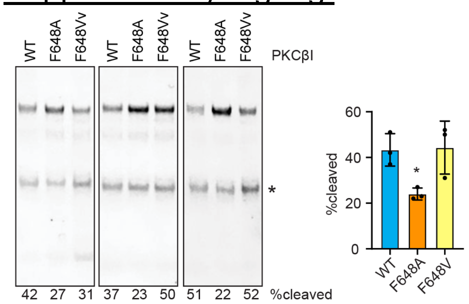

Endoxifen as an allosteric regulator of PKCb

The final experimental section of this manuscript addresses published data reporting an inhibitory effect of tamoxifen (TAM) on PKC and a more potent effect of its metabolite, endoxifen (ENDX). The authors start by assessing the inhibitory effect of TAM and ENDX on PKCbI and bII in an in vitro kinase assay. Both compounds exhibited lower IC50 values in the absence of lipids; however, given the hydrophobicity of these compounds, indirect effects of lipids on their availability in solution cannot be ruled out. As such, the interpretation of the results in Figure 5A should be treated cautiously.

The IC50 value is higher in the presence of phospholipid micelles than in the absence of them. However, the IC50 value of the kinase domain ($PKC\beta I^{cat}$) in the absence of phospholipids is similar to that of WT with phospholipids. We interpret this as the hydrophobicity of the phospholipids is not the most likely cause of the higher IC50, but we have now edited the text to comment on this possibility:

"Both ENDX and TAM were less effective inhibitors in the presence of Ca2+ and activating phospholipid micelles. Although it is possible that phospholipids reduce the availability of ENDX in solution, the IC50 is similar to that of a truncated, constitutively active PKCβI consisting of only the kinase domain in the absence of phospholipids (Fig. 5d) suggesting that the active conformation of PKCβI is less sensitive to inhibition by ENDX."

The authors also report thermal stability (DSF) measurements for PKCbI in the presence and absence of ENDX (Figure S7A). Although they claim that ENDX binds to both the N-terminal regulatory and kinase domains of PKCbI (pg 7, ln 15-17), the data do not support this conclusion. In fact, the melting temperature of full-length PKCbI (FL), the catalytic domain (CAT), and the N-terminal domain (NT) are unaffected (even slightly reduced) by ENDX (see table inset, Figure S7A). It is, therefore, not clear how the authors came to the conclusion that ENDX binds to PKC from this DSF data. The data and the conclusions from these experiments remain unchanged from the original manuscript, but do not support the authors conclusion that "differential scanning fluorimetry (DSF) analysis shows that both the N-terminal regulatory domain and the kinase domain bind to ENDX."

A reduction in the melting temperature in a DSF experiment can occur when the ligand traps a conformation that is less stable to thermal denaturation in the DSF conditions. We now mention this explicitly and cite two references that describe a reduction of Tm by a ligand (refs 61 and 62). We have repeated the DSF experiment to include more replicates and increase the confidence in the small shifts in Tm. Additionally, we have also assayed the known PKCβI inhibitor enzastaurin, which also decreases the Tm of PKCβI. Therefore, these data suggest that the decrease in Tm of PKCβI is consistent with binding of ENDX. We include the following in the revised manuscript:

"In line with our previous report indicating that ENDX binds to PKCβI[41], differential scanning fluorimetry (DSF) analysis showed that ENDX also decreases the Tm of PKCβI, further suggesting a direct interaction or ENDX-induced molecular alteration to PKCβI (Supplementary Fig. 7a). Although ligand binding is more classically associated with an

increase in Tm, a Tm decrease can also be induced by ligand binding[61,62]. Enzastaurin, a well-known PKCβI ATP-competitive inhibitor, decreased the Tm of PKCβI, suggesting that a reduced Tm is possibly a general effect of PKCβI inhibitors."

a

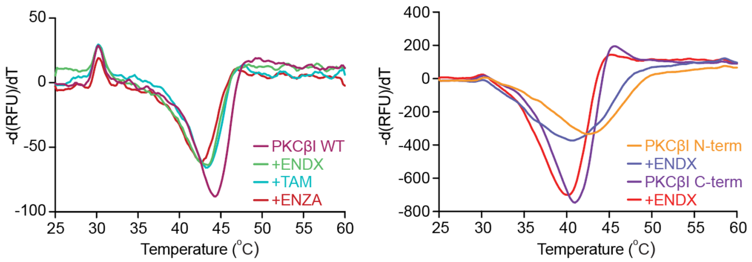

Protein	Tm (°C)	P-value
PKCβI FL WT	44.3 ± 0.1	
+ENDX	42.9 ± 0.1	4.3 x 10^{-4}
+TAM	43.4 ± 0.2	5.7 x 10^{-4}
+ENZA	42.7 ± 0.3	4.3 x 10^{-4}

Protein	Tm (°C)	P-value
PKCβI N-term	42.7 ± 0.2	
+ENDX	40.5 ± 0.2	1.5 x 10^{-9}
PKCβI C-term	41.0 ± 0.1	
+ENDX	40.1 ± 0.1	4.4 x 10^{-4}

Next, the authors support the conclusion that ENDX is an allosteric inhibitor of PKCbI by comparing the effect of ENDX on PKCbI in the presence or absence of lipids with the isolated kinase domain of PKCb (Figure 5B). ENDX has a stronger inhibitory effect on PKCbI in the absence of lipids than either PKCbI with lipids or the isolated catalytic domain, which the authors interpret as an allosteric effect of ENDX on PKCbI that depends on the intramolecular assembly of its regulatory domains. (Again, I would emphasize the aforementioned concerns regarding the hydrophobicity of ENDX and possible (indirect) effects of lipids on its inhibitory behavior). This is further supported by kinase activity data in Figure 5C that shows that ENDX is a non-competitive ATP inhibitor (it lowers Vmax without changing the Km for ATP, right panel), which is in contrast to the known ATP-competitive inhibitor enzastaurin, which does not affect Vmax, but increases the Km for ATP (left panel).

Minor comment: panels A and C each contain two graphs, which should be labeled separately for ease of reading and interpretation.

We have now split the original 5A panel into A and B panels, and split the original 5C panel into panels E and F.

To evaluate the effect of ENDX on the conformation of PKCbI in solution, the authors employed small angle X-ray scattering (SAXS). Figure 5D depicts the raw 2D scattering curves for PKCbI alone, +TAM and +ENDX, together with a table of the respective Rg and Dmax parameters derived from the data. The authors have added Guinier plots for their SAXS data (Supplementary Figure 7d) which confirm that their samples are generally well behaved, with the exception of their PKCbI+TAM sample, which shows evidence of low levels of aggregation (therefore Rg and Dmax should be treated cautiously). The manuscript also now contains a Materials and Methods section on the SAXS sample preparation and data collection, as well as a Supplementary Table summarizing the corresponding data parameters. Pair-distribution functions used to determine Dmax, however, are still missing. Overall, the evidence from SAXS that ENDX results in compaction of PKCbI is reasonably strong, as evidenced in the low-angle scattering regime in Figure 5D. However, the authors could make a stronger case by showing the respective Guinier plots for PKCbI ±TAM on the same plot in the main figure

(only a SAXS expert will spot and appreciate the difference in the raw scattering curves that the authors show).

We moved the raw scattering plot to Supplementary Figure 7d and replaced panel 5d (now 5g) with an overlay of the three Guinier plots. We have kept the individual Guinier plots and their associated residuals in Supplementary Fig. 7f as an overlay of the residuals was difficult to visualize. We have added the electron pair distribution *ρ*(r) plot as Supplemental Fig. 7e. With the new Guinier plots and *ρ*(r) plots the differences between the ENDX and vehicle/TAM samples is readily apparent.

Figure 5g:

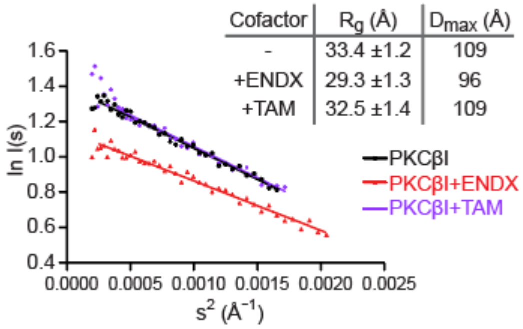

Supplementary Fig. 7d,e:

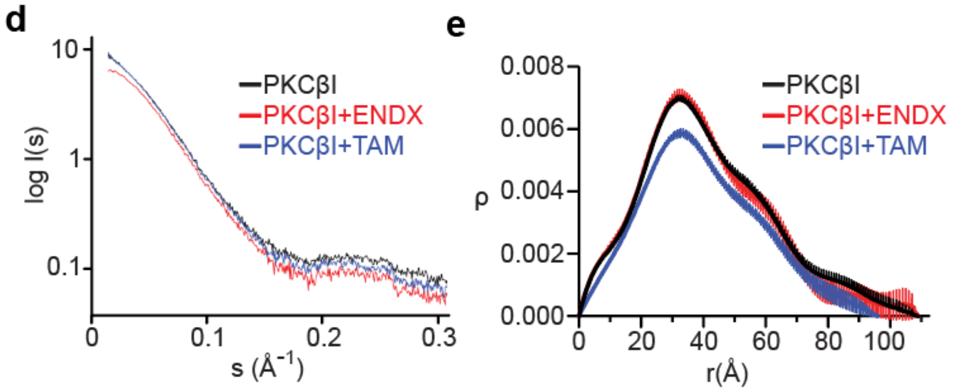

It is, however, somewhat perplexing that ENDX exerts an apparent compaction effect on PKCbI in solution, but has no stabilizing effect in the authors DSF assays (despite the authors' claims to the contrary. An allosteric inhibitor, that acts as a 'molecular glue' to stabilize intramolecular, interdomain interactions would be expected to have a fairly significant effect on the melting temperature of FL PKCbI. However, according to the authors' data in Supplementary Figure 7a, it has the opposite effect of lowering the melting temperature. This apparent contradiction undermines the authors' claims that ENDX is an allosteric inhibitor of PKCbI.

We certainly agree that *prima facie,* these data are perplexing. We don't have any structural information about how the ENDX leads to compaction of the PKCβI protein. As noted above for both ENDX and enzastaurin, as well as in the two cited references, a decrease in melting temperature in DSF can be caused by ligand binding. Although we

have been unable to determine a structure depicting the molecular basis of this effect, we can speculate that for example, a binding mode such as that observed for WNK1 inhibitors (Yamada et al., ACS Chem Biol, 2016) which insert between, and pry apart the kinase N- and C-lobes could be a molecular mechanism that would lead to a decrease in Tm by DSF, perhaps through increasing accessibility to the fluorescent dye as the protein approaches its melting temperature. Overall, a Tm decrease in the DSF assay is supportive of interaction between PKCβI and ENDX even though the Tm decreases. Furthermore, additional lines of evidence including limited proteolysis (Fig. 5h) and enzyme inhibition (5a-d,f) support direct binding of ENDX.

Using limited proteolysis again as a readout for conformation, the authors then compare the effects of ENDX and TAM on elastase cleavage of PKCbI. No comparison to PKCbI in the absence of either compound is shown as a reference. While the data presented show a clear difference in the cleavage pattern which is visible by eye, it is not clear what sort of model has been used to fit the data in the lower panel. In particular, fitting the cleavage of PKCbI in the presence of TAM appears to be questionable given that there is no significant change at any concentration of TAM. This remains unchanged from the original manuscript.

Cleavage by elastase in the absence of compound is shown in the lanes labelled "0" ENDX/TAM, namely lanes 2 and 9 of Fig. 5e. IC50 values were calculated by fitting the intensity of the band indicated by "*" as a function of ENDX concentration to a 4-parameter dose-response model using GraphPad Prism (now clarified in the methods section. The dotted line for TAM represented what a fit could look like if the bottom was constrained to zero, however, given the speculative nature of this fit we have removed the dotted line entirely.

Finally, the authors employ live-cell imaging to visualize the effect of ENDX on PKCbI translocation to the plasma membrane in MCF7AC1 cells treated with the phorbol ester PMA. The authors observed that ENDX promoted PKCbI translocation in the presence of PMA and that the PHLPP phosphatase inhibitor NSC117079 abrogated this effect (Figure 5F). Based on the authors' previous observations of the effects of ENDX, these findings are somewhat paradoxical. The authors claim that ENDX is an allosteric inhibitor of PKC that specifically targets its intramolecular conformation in an ATP non-competitive manner and renders it more compact. Intuitively, one might therefore expect an inhibitory effect on PKC translocation, much as has been observed for the allosteric inhibitor MK2206, which targets Akt. Instead, the authors observe the opposite. The data presented in Figure 5F and Figure S7C-D is difficult to assess for the following reasons:

- *the authors present only static snapshots of different patches of cells in each experiment. Without translocation kinetic data, averaged over many cells to account for variabilities in PMA and/or ENDX availability that result from the available membrane surface area and local environment of each cell in a 2D culture monolayer, it is impossible to assess with any degree of confidence what the effect of ENDX treatment is on PKCbI membrane translocation.*

We now include Supplementary Fig. 9, depicting a time course of recruitment of PKCβI to the plasma membrane after addition of PMA +/- pretreatment with endoxifen. We include 3 independent time courses with multiple cells visible in each that show membrane accumulation begins around the 3-6 minute mark in the PMA+ENDX samples, and is near a steady state at 15 minutes. In the PMA-only samples the effects are more subtle but appear to be near a steady state in later timepoints. The changes in the extent of membrane accumulation with ENDX pretreatment were readily apparent at 15 minutes. We have chosen to present single snapshots at 15 minutes to succinctly convey the effects of the indicated treatments and mutations on PKCβI membrane accumulation over the large number of experimental conditions examined. Indeed, there are many additional follow-up studies of interest, but many of these are beyond the scope of the present work and we hope to include them as parts of other manuscripts in the future.

The authors invoke the phosphatase PHLPP as a possible explanation for this effect on the basis that the reported PHLPP inhibitor NSC117079, as well as the PP1/PP2A inhibitor okadaic acid (OA), mitigated the increased localization. However, there are significant problems with this interpretation:

• The authors use a mutant PKCbI, T500A, which cannot be phosphorylated in its activation loop. ENDX, NSC and OA had no effect on the translocation of PKCbI T500A, compared to wt PKCbI. However, the implication, at least for NSC, is that PHLPP inhibition is responsible for this effect. However, PHLPP has been reported to be the phosphatase for the hydrophobic motif of PKC and not the activation loop (T500), and the validation of NSC as a specific PHLPP inhibitor is not very convincing, in the opinion of this reviewer. Furthermore, OA specifically inhibits a structurally different family of phosphatases to PHLPP, which implies that these compounds exert different effects on cells which cannot be rationally integrated into the same model.

In the HEK293F cell line used for this study the T500A mutation yields PKCβI that also does not become phosphorylated at the other two sites (T642 and S661). Thus, although only T500 is mutated, altered localization is due to lack of phosphorylation at all three sites. We note that although PHLPP1/2 inhibitor and PP1/2A inhibitors target different phosphatases, both phosphatases dephosphorylate PKCs — although relative rates, extents, or contextual differences have not yet been clearly established in the literature. As noted by the reviewer the validation of the PHLPP1/2 inhibitors is not as robust as the very well-characterized okadaic acid inhibitor. Individually, inhibiting a single class of phosphatases would have its own set of caveats — phosphatases act on other proteins which could potentially lead to secondary changes in PKCs, or mediate off-target effects. For precisely this reason we have examined the effects of inhibiting these two different classes of phosphatases which share PKCs as substrates, while other targets (and potential off-targets) would differ. See also the new in vitro dephosphorylation data included in response to the reviewer's next comment.

• The authors reference autophosphorylation of T500, but the prevailing model in the field is that this is accomplished by PDK1. I do not necessarily subscribe to this model, but the authors should acknowledge it, since inhibition of autophosphorylation is intrinsic to their

model. To support their model, the authors should demonstrate that ENDX inhibits PKCb T500 autophoshorylation in an in vitro kinase assay containing purified, recombinant, and dephosphorylated PKCbI. This should be possible given that the authors have well-behaved, purified proteins at their disposal.

We now include an *in vitro* (de/auto)phosphorylation assay in the revised manuscript to address this point (Supplementary Figs. 10d–g). We use phos-tag SDS-PAGE gels, which contain a phosphate-interacting ligand that slows migration of phosphorylated proteins, allowing visualization of individual phosphorylation states (0, 1, 2, or 3 phosphoresidues). We note that the 1P and 2P bands are poorly resolved and overlap in some of the gels. In panel S10d we show that partially dephosphorylated PKCβI (1P, 2P) can rephosphorylate itself, while fully dephosphorylated PKCβI (0P) does not appear to be able to do so in these experimental conditions. We next established conditions for an in vitro reaction that contained both PP2A and ATP to achieve a balance between de-phosphorylation and (re-)autophosphorylation. In these conditions the protein consists primarily of 2P and 3P PKCβI (panel S10e), suggesting PKCβI partially rephosphorylates itself. We next compared the effect ENDX on the phosphorylation state of PKCβI in these conditions and observe a shift to lower phosphorylation states with accumulation of 0P PKCβI (panel S10f), suggesting ENDX inhibits autophosphorylation, which tilts the balance of activities towards dephosphorylation. This is in contrast to the ATP-competitive inhibitor ENZA, which does not lead to an increase in dephosphorylation of PKCβI. We feel that collectively these experiments recapitulate *in vitro* the molecular events that would trigger PKCβI dephosphorylation in cells. We have added these data as Supplementary Figures 10d-g along with the following text to the manuscript:

<u>To test this model, we developed an experimental system to assess PKCβI phosphorylation using a reconstituted in vitro system with recombinant proteins. We established a phos-tag PAGE assay capable of resolving the distinct phosphorylation states of PKCβI. Recombinant PKCβI, purified from expression systems, contains three phosphorylated residues. When analysed on a phos-tag gel, it appears as four distinct bands (Supplementary Fig. 10d, lane 2), representing species with 0, 1, 2, or 3 phosphorylated serine/threonine residues. We note that the band corresponding to the singly phosphorylated form (1P) is faint and can vary between experiments. PKCβI was then treated sequentially with PP2A to remove some phosphate groups, followed by inhibition of PP2A with okadaic acid, and then ATP was added to assess PKCβI's ability to autophosphorylate (Supplementary Fig. 10d, lane 3). These results indicate that PKCβI can partially restore its fully phosphorylated (3P) form through autophosphorylation. However, the persistence of the 0P band after ATP addition suggests that only the 1P and 2P forms are competent for autophosphorylation and restoration of enzymatic activity. Co-incubation of PKCβI with both PP2A and ATP simultaneously leads to a stable equilibrium consisting of primarily 2P and 3P species (Supplementary Fig. 10e). Using this reconstituted system, we next evaluated the impact of ENDX on the phosphorylation/dephosphorylation balance of PKCβI (Supplementary Fig. 10f). ENDX disrupts the phosphorylation equilibrium by preventing autophosphorylation, thus shifting the balance toward dephosphorylation yielding greater amounts of the 0P PKCβI. Interestingly, treatment with the ATP-competitive inhibitor enzastaurin did not lead to</u>

accumulation of dephosphorylated PKCβI (Fig. 10g), indicating that ENDX's mechanism of action is distinct from that of ATP-competitive and staurosporine-derived inhibitors. Together, these findings provide a mechanistic framework for how ENDX can lead to accumulation of PKCβI at the plasma membrane.

Supplementary Figure 10d-g:

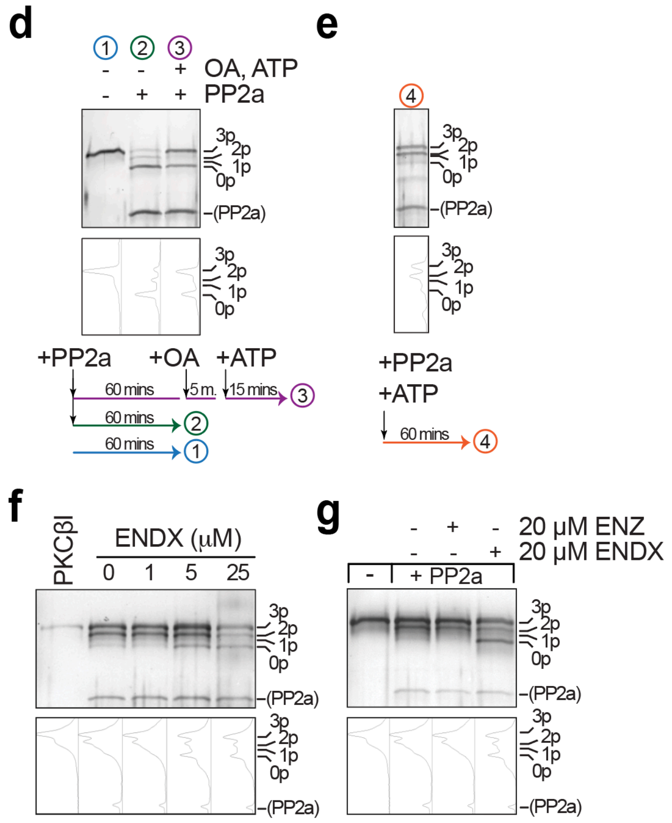

Minor comment:

The authors reference Supplementary Figure 7C for the effects of OA on membrane accumulation, but the correct reference is Supplementary Figure 8C.

Thank you. We have corrected this in the revised manuscript, which is now a reference to Supplementary Figure 10a.

In summary, the assertion that ENDX is an allosteric inhibitor of PKCbI is not sufficiently well supported by the data the authors present.

Our conclusion that ENDX is an allosteric inhibitor of PKCβI is based on multiple lines of experimental data. We first established that ENDX is a PKCβ inhibitor through biochemical kinase inhibition assays. Results from our assays additionally showed that ENDX inhibits PKCβ via a non-ATP competitive manner in contrast to the ATP-competitive inhibitor ENZA. We collected SAXS data which demonstrated that ENDX induces conformational changes to PKCβI and this conformational change is also

captured in limited proteolysis data. Allosteric inhibition by ENDX manifests as a defect in PKCβI autophosphorylation, leading to an increase in membrane localization observed in our microscopy data. This effect was examined side-by-side with ENZA treatment, which showed distinct features and further suggesting a different mechanism of inhibition by ENDX.

Reviewer #3 (Remarks to the Author):

In their article "Molecular Basis of Allosteric Regulation and Pharmaceutical Targeting 1 of Protein Kinase Cβ" Cong et al report their crystallographic results, biochemistry results and analysis on an important class of proteins in the Protein Kinase C (PKC) family.

On the positive, the data reported in this paper are very worthwhile and meet the barrier for a high ranking journal. These protein and these crystal structures have been sought after for many decades. The resolution and completeness of these structures is very informative and has the possibility to guide further biological studies and even help in drug discovery.

The reporting of these results faces many challenges. The proteins are complex with multiple domains, there are multiple functions and finally their crystal structures are also very complicated. For such a complicated system and results, the authors must have a very clear explanation of their results and conclusions. Unfortunately, I didn't feel like the authors hit that mark in their current draft. There were many confusing issues with the presentation of these results in this paper. These issues made the paper almost unreadable as the reader is made to look up, in other literature, whether the authors meant one thing or another. I don't think these issues are insurmountable. They require the authors to read their paper multiple times and assess whether a person outside the lab would also understand, without second hand information. I definitely did not catch all issues as they accumulated and I got bogged down as I read the paper.

We thank the reviewer for this feedback. As we are able to add some length to the paper, we have expanded the language throughout, resulting in an increase of ~30% in the word count, which we hope will address the reviewers concern about readability.

Starting from the beginning
Phosphorylation is required for proper folding. Don't think this is true, protein folds and phosphorylation is part of function and regulation.

Although PKC should remain folded when unphosphorylated, newly synthesized PKCs associate with the HSP90/CDC37 chaperone complex, and acquisition of proper phosphorylation is associated with chaperone-mediated folding of the kinase domain. It was this latter context that we were referring to, and we have revised the text accordingly:

"The C-terminal kinase domain acquires phosphorylation at three Ser/Thr residues during CDC37/HSP90-mediated protein folding[19,20] that are required for kinase activity[15]."

PBI is used once in the paper without definition.

We now define this as a "Phox and Bem 1 (PBI) domain"

Very little is said about the active site of the protein. Sure this may not be the main point of the paper, but a sentence that comments on the active site of the protein and says the results are identical to some other literature would be good. Worth paying some homage to the active site. Along similar lines, the substrate that is acted on is ATP not DAG. DAG is a regulatory molecule, as far as I could tell. Also the paper would benefit from an understanding of whether the active or inactive form is "meant" to have the ATP substrate bound.

We have edited language throughout the paper such that DAG is referred to as a regulatory molecule or ligand. We did not focus our discussion on the active site itself, as it has already been described and it was indeed not the main focus of our paper. We now include additional details describing the active site, rearrangements of the glycine-rich loop, and differences in active site conformation that are observed when the PS is present or absent. We have added the following text and its associated figure panel:

"The AMPPNP nucleotide is only present in the monomer with a PS-bound kinase, and the active site is slightly more open when the nucleotide is absent, suggesting the kinase domain could toggle between these conformations in order to release and re-bind nucleotide. In the absence of nucleotide (crystal form I, chain A), the glycine-rich loop (^{351}GSFGG^{354}) adopts a conformation that positions F353 in the ATP binding site in an arrangement similar to that seen for PKCβII bound to a bisindolylmaleimide inhibitor[26]. In Chain B the glycine-rich loop adopts an extended conformation that displaces F353 from the nucleotide binding site and unmasks three peptide dipoles that interact with the phosphates of the Mn^{2+}–AMPPNP (Supplementary Fig. 5c). Although these structural features alone do not portray the biological interdomain interactions of a single PKCβI enzyme, they indicate that all monomers contained two conserved kinase domain elements, namely the NFD (Asn–Phe–Asp) motif and helix αB in conformations, which suggests the kinase domains are in the activated and catalytically competent state[52]. Thus, the unbound and peptide–nucleotide–bound states observed here likely represent states in the catalytic cycle of PKC kinases as proposed for the closely related enzyme protein kinase A[53]."

Supplementary figure 5c:

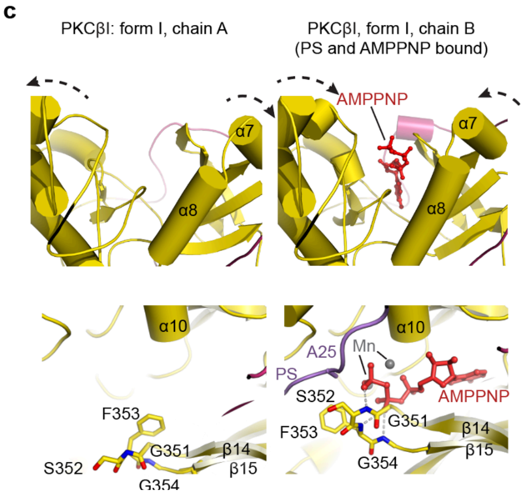

In the Results section titled "Molecular basis of PKCβII autoinhibition and …." suddenly there are two chains. I assume this is intended to refer to two chains in the asymmetric unit. However, this is completely up to the reader to surmise.

We have added the following statements to clarify the observation of two PKCβII chains in the crystal structure asymmetric unit as follows:

"<u>We obtained crystals of PKCβII in the presence of a non-hydrolysable ATP analogue AMPPNP and solved a 3.3Å structure that contains two individual chains of full-length PKCβII within the asymmetric unit (Figs. 1c, d; Supplementary Table 1)</u>."

"<u>We assigned all domains (PS, C1a, C1b, C2, and kinase) to the two PKCβII chains within the asymmetric unit based on…</u>".

I'm not sure whether the authors intended to use the word "lipid" as shorthand for phospholipid or just the hydrophobic portion of a phospholipid. If the latter, then whenever there is "lipid" binding, near to this hydrophobic area there should be something on the protein that contends with the hydrophilic part.

We used the term lipid as shorthand to refer to phospholipids, and we have revised the manuscript to refer to these as phospholipids. The C1 domains contain a ring of charged residues adjacent to the hydrophobic ones that interacts with the hydrophilic and anionic region of the phospholipid membrane. See studies by Bittova et al (Pubmed ID 11029472) and Katti et al (Pubmed ID 35577811) that examine the interaction between charged C1 domain residues and the polar component of phospholipids.

The description of the differences between the different isoforms of the PKC were quite good. The work that went into clearly describing the difference paid off.

We thank the reviewer for their positive feedback.

The text that is associated with Fig 2 and figure itself were confusing to me. The overlapping of the kinase domain and the placement of the N-terminal domains was specifically confusing. At some point in the text it says "two of them were comprised of similar molecular interactions", then looking at the figure, there are three very different interactions. Further the colors have changed – keeping at least one of the N-terms in similar domain colors as Figure 1 would help with orientation.

We have expanded the discussion of this figure to more clearly describe this analysis and its interpretation. The similar molecular interactions refer to the three chains present in the "N-term 1" arrangement, and the three chains present in "N-term 2" arrangement. We used colors that are distinct from the domain arrangements in Fig. 1, as each individual domain (PS, C1a, C1b, and C2) is colored uniquely in Fig 1 (and others), whereas in Fig. 2a all of the N-terminal domains are grouped together and coloring each subdomain would yield a figure panel with 10 different domain colors and be difficult to interpret. We have revised the manuscript to describe this.

Revised text:
"To identify the biological unit of the multi-domain PKCβI enzyme and interdomain regulatory interactions we explored the local symmetry environment in each PKCβI monomer comprised of an interface between a kinase and N-terminal domains within 4Å in the crystal lattice. The kinase domains from all three monomers (one from crystal form 2 and two from crystal form 1) were aligned to identify common interaction modalities present among the domain arrangements that comprised the crystal lattice. Each kinase domain formed an interface with three adjacent N-terminal domain modules, and an alignment of each kinase domain and surrounding N-terminal modalities reveals that and two were comprised of molecular interactions that were present for all kinase domains (Fig. 2a; N-term 1 & 2), while the third (N-term 3) was variable among the three monomers (Supplementary Fig. 5d). We therefore hypothesized that the interfaces between N-terms 1 & 2 and the kinase domain represented physiological interactions that would be present in solution. The interface with N-term 2 yields a conformation in which the C1a and PS engage with the kinase domain similarly to PKCβII (Fig. 1d), suggesting that it represents the auto-inhibited conformation of the PKCβI enzyme. The interface with N-term 1 positioned the C1b and C2 domains, such that they interact with the kinase domain C-lobe, distal from the active-site in a conformation that has not been previously described."

Overall, a small bubble cartoon of the asymmetric units in the crystals would have been very helpful. This would have particularly helped in underscoring the challenges of the project and why much analysis was done.

Thank you for this suggestion. We have added bubble cartoons to Supplementary Fig. 3f and Supplementary Fig. 5a to illustrate the asymmetric units and domain swaps that are discussed in our analysis.

Supplementary Figure 3f:

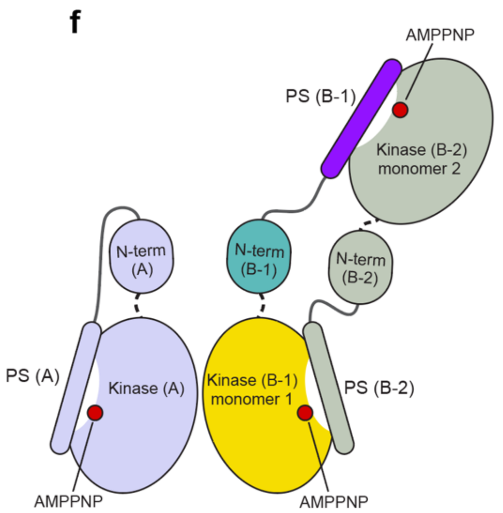

Supplementary Fig. 5a:

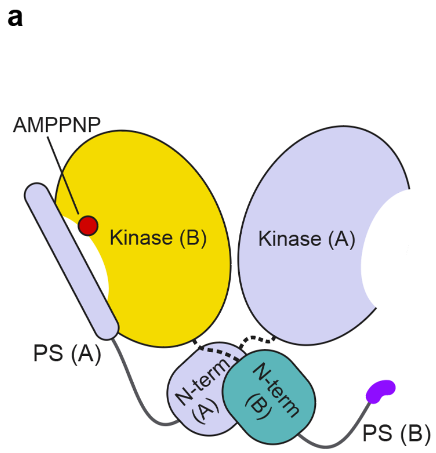

The lipid-lever phraseology was confusing though the alliteration is appreciated. As far as I followed the proposed mechanism, and especially as I followed Fig 3a, a portion of the protein is sequestered into the phospholipid membrane. This process removes contacts that are made between domains in a non-membrane state. First, my complaint here involves the lipid vs phospholipid issue I raised earlier. The protein does not only need to prefer the hydrophobic section, but there must be something that deals with the strong phospholipid contacts. Second, this is more like a phase sequestration or membrane integration process. Would be good to note in Fig 3a whether the drawn lipids are "to scale" and if not, to make some attempt to make it so.

See also our response to Reviewer 2's comments above, as well as the earlier question raised by this reviewer in relation to contacts with the hydrophilic section of the phospholipid membrane. Supplementary Figure 6e also depicts charged residues that interact with the polar region of the phospholipid bilayer. We have revised the diagram in Figure 3 to better approximate the scale of the lipid bilayer to PKC in the panel on the right. Phospholipid bilayers and the C1 domains are both about 30-40Å across, and they

are drawn approximately to scale. The middle section of the figure still shows an enlarged view of the PS-C1a-kinase complex as shrinking it to scale yielded crowded labels around it that made it less readable.

Figure 3c:

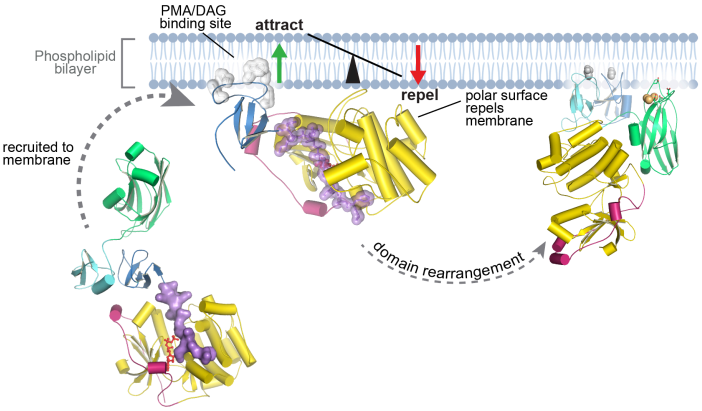

Supplementary Figure 6e:

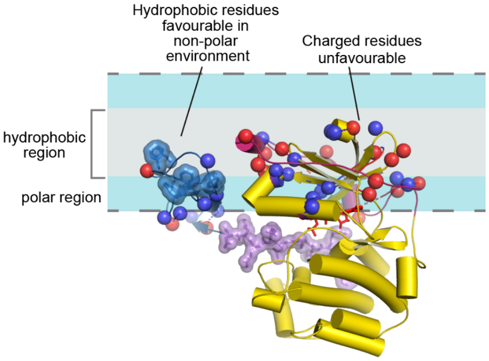

We have also modified our description of the conformational change driven by phospholipid membrane association to emphasize that the C1 domains are sequestered in the membrane:

<u>"The C1a and PS domains would need to first undergo the slow process of dissociating[49] and embedding in the membrane, which would prevent the otherwise rapid reassociation with the kinase domain. In this way, the lipid membrane effectively serves as a lever that holds the PS domain away from the catalytic domain, with the net effect of sequestering the PS and C1a domains from the kinase domain via a mechanism that we have termed a "lipid-lever".</u>

The only substantive issue I have with the science, rather than way or specificity with which the information was conveyed, was the lack of a solution analysis. There appears to be some SAXS data available (though it appears to be collected both at the Advanced Photon Source and the Advanced Light Source – per text vs acknowledgement). Quite easily the full length solved structure (or a model where the missing residues are modelled in) could be put into a SAXS calculator like crysol or foxs and the result could be compared to the measured SAXS profile. If the match is poor and conformational change is

suspected for the difference, the program BilboMD or EOM could be used to help define the conformational flexibility required to fit the data.

One of the challenges with SAXS is the low information content contained within the scattering data. We have chosen not to include this analysis because there are too many degrees of freedom within our model of PKCβ, which precludes definitive conclusions from SAXS modelling. For example, in the auto-inhibited conformation we would model the N-terminal 18 residues as well as the linkers between the C1b-C2 and C2-kinase domains as flexible. Furthermore, the C2 domain is variable in position relative to the kinase domain and would be modelled as a mobile domain. The resulting EOM runs yields a large number of possible solutions, and although it fits well to the SAXS data, it isn't really a meaningful result. Thus, we have only used SAXS to evaluate whether global changes in PKCβI occurs in the experiment. SAXS analysis on phospholipid micelle-bound PKCβI in the active conformation would not be possible due to scattering from the lipid micelles.

Details about the beamline usage are contained in the methods section. SAXS data was collected at the SIBYLS beamline (12.3.1) at the ALS.

I apologize to the authors for being critical of the writing after they clearly have put so much effort into the science. Going through rounds of review is never fun. However, having made some effort in deciphering the text, I think the hardest part is done. With a bit of thoughtful revision (thinking about what the reader needs to know and will assume), the effort will come through much more clearly. The conceptual issues I describe were exemplary of distracting issues that prevented me from being able to read through the paper without having to research whether the authors meant one thing or another. I suggest going through the entire paper for issues like those raised here. I'm pretty sure there are more that I didn't catch.

Again, we than this reviewer for their insightful comments. In addition to addressing the comments posed by the reviewers here, we have expanded our description and discussion throughout the manuscript to better describe our results and expand our discussion. We hope the new version is a better read for everyone!

Reviewer #2 (Remarks to the Author):

Major comments

• Supplementary Figure 3b. The authors include new data to support the C1b-PS interaction they observe in their crystal structures. Mutation of G24Q in the PS segment increases activity, rather modestly, at high concentrations of phosphatidylserine-containing micelles. However, I am concerned about both the interpretation and depiction of this data:

o The activating effect of the mutation is not seen in the absence of lipids, despite the authors' claims that it destabilizes the inactive conformation. This weakens the authors' claim that G24 is part of a physiologically relevant PS-C1b interface.

As noted by Reviewer #2, basal activity in the presence of uncharged micelles (labelled “0”; please also see the response to the subsequent point clarifying the lipid composition for this experiment and updated Fig S3e) is not significantly greater for G24Q than WT. While G24Q is designed to destabilize the C1b interface, it is not clear that this alone would be sufficient to increase activity of the unactivated kinase. For example, the C1b-kinase domain interface proposed by Leonard et al. would also remain functional, and the C1a/PS-kinase interface remains intact. The relative strength of the C1a/PS-kinase vs C1b-kinase interfaces would also be important for interpreting this experiment, because if the C1a/PS bound more tightly than C1b, then defects caused by G24Q may not significantly alter unstimulated kinase activity. Therefore, the differences between G24Q and WT become apparent with sub-optimal phospholipid activation, where the weakened C1b-kinase interface is less able to compete for binding to the phospholipid micelles.

o The x-axis is labeled as % lipid content, which has no meaning. It is not clear what the authors mean, but the lipid content of these reactions should be expressed in standard concentration terms.

o It is not clear whether DAG was included in these micelles and, if so, at what concentration. Since phosphatidylserine (PS) binds to the C2 domain and not the C1b domain being investigated in this experiment, the presence of absence of DAG is important for interpretation of the result. In fact, as the authors state, the binding site in the C2 domain for PS is solvent-exposed, such that PS alone would not be expected to activate PKC.

We thank this reviewer for pointing this out. The legend for this figure has been edited to indicate the percentage of PS, DAG, and Tween-20 used in this experiment. Both DAG and PS concentrations were altered in this experiment.

The new graph for figure 1g (formerly S3b) is:

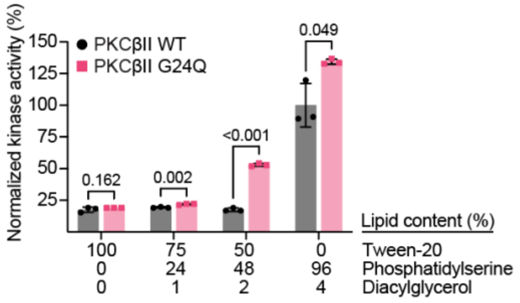

o Finally, the experimental validation of the authors' structure(s) is extremely important for an assessment of their physiological relevance. Such data are therefore an integral part of the authors' narrative and should not be relegated to Supplementary Data.

We have moved Figure S3b to the main text, where it appears as panel 1g

• Figure 2d. The authors use limited proteolysis to test the effects of mutation of the C2-kinase domain interface in their structure. This experiment was complicated by the degradation of PKC lacking its PS segment, so the authors engineered a cleavable variant with which they could remove the PS segment post-purification. With the purified proteins in hand, they tested their susceptibility to elastase cleavage as a proxy for their stable compact intramolecular association. However, I do not agree with their interpretation of the results:
o Both PS-cleaved mutant proteins are already significantly degraded at t=0 in comparison to the non-cleaved proteins, presumably because the introduction of the Prescission cleavage site or cleavage itself made the proteins more susceptible to degradation by contaminating proteases.
o It is notable that the PS-cleaved proteins in Figure 2d look considerably less pure than their non-cleaved counterparts, so interpretation of the cleavage patterns over time should be treated cautiously.
o The elastase-mediated increase in the 40 kDa band corresponding to the kinase domain, which the authors use to assess stability of the proteins, is actually lower for both PS-cleaved mutants than their non-cleaved counterparts. If one accepts this, then this experiment does not support the authors' conclusion that the Y285R mutation destabilizes the C2-kinase interface.
o This does not mean that the authors' structure is not physiologically meaningful, it just means that the mutagenesis experiment presented in Figure 2d is not sufficient to validate the interface. As such, I would not support its publication, either as a main or supplementary figure panel.

This experiment was designed and included to address the question raised by the reviewer as to whether destabilization of both the active and inactive conformations of PKCBI could somehow be detected. While far from ideal, the experiments presented in Fig. 2d have been the best that we were able to obtain after carefully optimizing the protein preparation method to minimize the amount of contaminating proteolysis products. Although we note that the changes caused by the Y285R mutation in full-length vs PS-cleaved are broadly consistent with the proposed model, we appreciate that this data is difficult to interpret. Thus we have chosen to remove this figure panel and its associated text from the manuscript at this time.

• Figure 5. The authors have used an inconsistent coloring scheme that makes interpretation of their data unnecessarily complicated. For example, Fig. 5g uses black, red and magenta for the three samples, while Figure S7d,e use black, red and blue for the same samples. Although the PDFs in the rebuttal figure S7e do not correspond to the particle parameters listed in Figure 5g, the authors seem to have fixed this problem in the actual supplemental figure. (The rebuttal figure S7e mixed up PKCbI+ENDX and PKCbI+TAM? (the PKCbI+TAM curve clearly tends towards a lower Dmax than the other two curves, but, according to 5g, it should be PKCbI+ENDX that tends towards the lower Dmax).

We thank the reviewer for the very careful read. We did miss the figure error in the version that was included in the rebuttal, and the version included in the manuscript for S7e was correct. Magenta was used for figure 5g since the overlapping blue and black colors used previously were difficult to visualize. In the revised version we have used a lighter shade of blue for the TAM sample that is visible adjacent to the black symbols, and is consistent with other figure panels.

The revised panels 5g now appear as follows:

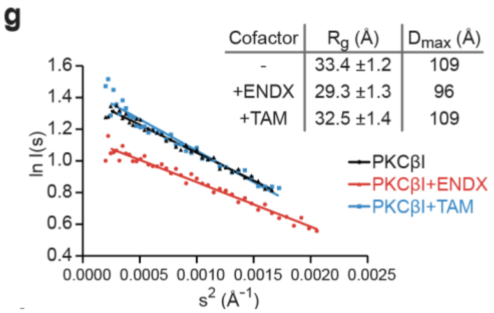

• Figure 5, SAXS analysis. Although the employment of SAXS is ostensibly to look at the effects of ENDX and TAM on the compactness of PKCb, the authors miss an easy opportunity to compare their solution scattering profile to the high-resolution structure of autoinhibited PKC. Assuming that they included a non-hydrolyzable ATP analog in their PKCb sample, they could reasonably compare the theoretical solution scattering of their model for autoinhibited PKCb to their experimental data using CRYSOL. This would go a long way to helping substantiate the physiological relevance of their crystal structures, given the obvious impact of lattice contacts and domain swaps that they have alluded to themselves. This requires no additional experimentation and a very simple data analysis. Reviewer #3 suggested the same thing, although the authors have argued against it. In my opinion, however, the authors' model for autoinhibited PKCb is complete enough to compare its solution scattering with their experimental data, with only ambiguities about the positioning of small linkers that will not influence the overall scattering curve to an appreciable degree at the resolution of SAXS.

We now include an analysis of the full length PKCβI, as the SAXS samples did indeed include non-hydrolyzable ATP (AMPPNP). We used the proposed inactive conformation generated by the domain swap as depicted in Supplementary Figure 6F and analyzed it using CRYSOL. Overall, the fit is reasonable, with comparable solution scattering parameters. We also utilized the program EOM (Ensemble of Mixtures) to model the disordered residues in the linkers and N-terminus, which further improved the fit to the experimental data, revealing reasonable congruence between the inactive conformation of PKCβI that we propose based on the crystal structure and the solution scattering data.

We have added Supplementary figure 7g and text describing this SAXS analysis:

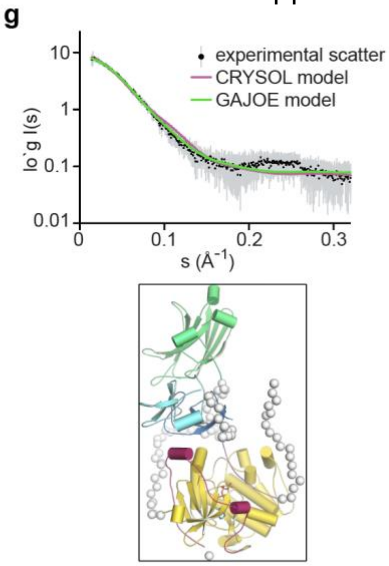

Figure Legend: (upper) Comparison of observed scattering with theoretical scattering generated from the model of PKCβI in the inactive conformation generated by CRYSOL or that from EOM used to model the unobserved residues. Black dots indicate mean pixel intensity, grey error bars, s.d. (lower) Model of PKCβI in the inactive conformation with domains colored as in Fig. 4a, with dummy residues modelled by EOM as grey spheres.
A comparison of observed scattering for the vehicle treated PKCβI with that calculated from the crystal structure in the inactive state (Supplementary Fig. 6f) using CRYSOL reveals a good fit with a χ^2 value of 0.37 (Supplementary Fig. 7g). Modelling the unobserved dynamic residues that are not observed in the crystal structure further improved the χ^2 to 0.18, consistent with PKCβI adopting the inactive conformation in solution.

Minor comments
• Pg 2, ln 32 and pg 3, ln 20. The authors claim, misleadingly, that no structure of a full-length PKC has been published to date. In fact, the structure of rat PKCbII, which the authors reference, is of the full-length protein, albeit with missing electron density for the PS and C1a domains.

We have reworded these statements to align with the domains in the previous PKCβII structure being unobserved, rather than not present. The text has been revised as follows:

"What remains uncertain is the role of the unobserved C1a domain and…"

"Here, we report the first crystal structure of full-length PKCβII containing electron density for all (PS, C1a, C1b, C2, and kinase) domains which reveals…"

• Pg 3, ln 37 and pg 10, ln 43. The authors should be careful when referencing PHLPP as a PKC modulator. It has recently been shown that PHLPP2 is a pseudophosphatase with no catalytic activity (Husremovic et al, PNAS 2025). The effects of NSC117079 are therefore likely not the consequence of PHLPP inhibition.

We thank the reviewer for bringing this recent manuscript to our attention. The function, activity, and effects of NSC117079 on PHLPP1/2 are not very well characterized. As Husremovic et al. point out, PHLPP1/2 (or at least PHLPP2) may not be active phosphatases. That said, the effects of NSC117079 lead to higher levels of phospho-PKC (see for example Taylor et al., Osteoarthritis and Cartilage Open, 2021), which is a relevant effect for counteracting the decrease in phospho-PKCBI caused by ENDX in this study. Therefore, we have added the following language to the results and discussion sections to better convey this consideration:

Pg 10, ln 43: "Plasma membrane accumulation was mitigated by addition of NSC117079, which has been proposed to be a pleckstrin homology domain leucine-rich repeat protein phosphatase (PHLPP1/2) inhibitor 65…"

Pg 13, ln 36: "However, a recent report has proposed that PHLPP2 is a pseudophosphatase, and therefore it should be considered that the effects of NSC117079 could be mediated by non-phosphatase effects on PHLPP2."

Reviewer #3 (Remarks to the Author):

The manuscript is easier to read and much clearer then the first draft. Hopefully, the effort will lead to higher citations.

We thank Reviewer 3 for their consideration of our response and revised manuscript!

Major comments

Figure 1g, ln 154-159. The authors have addressed my concerns with the presentation of this data, adding the molar percentages of the components in their micelles to the x-axis. Although the use of TWEEN20-based micelles in these assays is a strange choice, particularly since the final condition contains no TWEEN20, the comparison of wt and mutant suggests that G24Q indeed sensitizes PKCb to activation in the presence of physiological agonists.

TWEEN-20 was used as a non-activating, non-ionic detergent to dilute the PS/DAG phospholipid in micelles. There is no TWEEN in the final condition as it is composed of only PS/DAG.

Figure 2d. The authors have removed the problematic limited proteolysis experiments from the manuscript. I appreciate their efforts, but I think it is appropriate to acknowledge the deficiencies in the experiment.
Figure 5. The authors have fixed the inconsistent coloring scheme.
Figure S7g. Additional SAXS analysis. The authors now present evidence that their crystal structure of autoinhibited PKCb is a reasonable fit to its conformation in solution, as evidenced by its solution scattering (SAXS).

We again thank the reviewer for their helpful suggestions that led to these improvements in the manuscript.

Minor comments
There is still a referencing error in the introduction: the authors claim, misleadingly, that no structure of a full-length PKC has been published to date. In fact, the structure of rat PKCbII, which the authors reference, is of the full-length protein, albeit with missing electron density for the PS and C1a domains. The authors have fixed the reference to this study in their discussion, but not in the introduction. This is a minor issue that is easily fixed and the authors are correct in their assertion that a complete mechanistic understanding has been elusive in the absence of structural information on the PS and C1a domains that were not visible in this structure, but it should be phrased as such.

We have corrected this statement in the introduction to read as follows:

"However, no structure of any full-length PKC enzyme with electron density for all domains including the PS and C1a domains has been solved to date rendering a complete understanding of the molecular mechanisms of PKC regulation and activation elusive."

The authors have addressed concerns that the effects of NCI117079 may not be mediated by inhibition of PHLPP, but the sentence that references this study in the discussion implies that the compound still influences PHLPP2 behavior, for which, if you take away inhibition of phosphatase activity, there is no evidence. The authors' findings rather imply that NCI117079 exerts effects on cells that are not mediated by PHLPP. Whilst this might be inconvenient to the authors' narrative on PKCb phosphorylation, I believe that this caveat needs to be appropriately acknowledged. Again, this is a minor issue, but it significantly affects the reader's interpretation of the authors' findings. This is not a criticism of the authors for having used the compound, since they were not aware of the new study when this work was carried out, but I urge them to them to be precise and transparent in reporting its findings.

The literature has conflicting reports on PHLPP1/2 phosphatase activity. The 2025 PNAS report could not detect phosphatase activity for PHLPP2, and demonstrated that contaminating PP1/2A activity is present in PHLPP2 purified from eukaryotic cells. On the other hand, Sierecki and Newton (Biochemistry 2014) report that both PHLPP1 and PHLPP2 isolated from E coli do possess

phosphatase activity. We appreciate that there are differences in the approaches used to assay PHLPP1/2, and that this is an important issue, however, this is an ongoing area of study and is beyond the scope of the current manuscript. In the context of the experiments presented herein as they relate to PKCβI/II okadaic acid also reverses the endoxifen-driven accumulation at the plasma membrane, which provides an additional line of evidence that phosphatases play a role in this event. Thus, to better reflect the uncertainty with NSC117079 we have modified the following statement in the discussion to acknowledge the possibility that NSC117079 could be targeting other proteins:

"However, a recent report has proposed that PHLPP2 is a pseudophosphatase, and therefore it should be considered that the effects of NSC117079 could be mediated by proteins other than PHLPP2"

Summary

Protein Kinase C (PKC) is a family of 10 mammalian iso-enzymes that transduce the signal generated by phospholipase C (PLC) into appropriate downstream responses in the cell. PKCs have been implicated in a number of disease pathologies and regulate a diverse array of critical intracellular signaling pathways that make the study of their regulation incredibly valuable. Unfortunately, decades of study have failed to elucidate the complex regulatory mechanism(s) of these kinases at atomic resolution. The authors of this manuscript present a series of X-ray crystal structures of two splice variants of PKCb, which they use, together with a series of accompanying biochemical and cell biological experiments, to rationalize the mechanism of PKC activation by calcium, phospholipids, and diacylglycerol (DAG). In addition, the authors report the characterization of endoxifen, a metabolite of the anti-cancer drug tamoxifen, as an allosteric inhibitor of PKCb. The topic is of broad interest to a general audience, including basic, translational and clinical scientists. The study, however, falls short in a number of areas and, in the opinion of this reviewer, there are significant reasons to doubt some of the conclusions that the authors make on the basis of their data. In particular, serious concerns are raised about the quality of some of the supporting biochemical and biophysical data, which the manuscript heavily depends on to support the authors' structure-based hypotheses.

Major Comments

Structure of PKCbII reveals architecture of autoinhibited state.

The authors have determined the structure of full-length PKCbII in which 2 chains are observed in the ASU. The first chain (chain A, presumably) forms an intramolecular complex in which electron density is visible for 91% of the chain, while the second chain forms a domain-swapped interface between its regulatory domains and the kinase domain of an adjacent protomer in the crystal lattice. In this second instance, the inter-molecular interface is near-identical to the intramolecular interface observed in chain A. The structure represents the most complete picture of any PKC isoform to date.

The catalytic cleft of each kinase domain is engaged by a pseudosubstrate segment (PS) (either its own, or that of its domain swap partner), consistent with the known mode of substrate:kinase interaction. The DAG binding surface of the C1b domain is observed to be buried in intramolecular contacts with the PS and kinase domain, in contrast to a previously reported structure of PKCbII in which the C1b domain was observed to form an intramolecular contact with the N-lobe of the kinase domain (Leonard et al., Cell 2011), an

interface that was validated with membrane translocation assays in cells both in the original manuscript and in a follow-up manuscript published by the same group (Lucic et al, JMB 2016).

In the original submission of this manuscript, it was difficult to assess the physiological relevance of the reported C1b-PS-kinase interface in the absence of any mutational data. The authors have added kinase activity data on a mutant, G24Q (Supplementary Figure 3D) to substantiate this interface. Although the data presented supports their claims, the data points have no error bars (presumably n=1) and the y-axis scale is '% kinase activity', where it appears that the authors have normalized their data to the signal from the highest wt concentration. At the very least, this experiment needs to have sufficient replicates and an appropriate activity scale.

The DAG-binding surface of the C1a domain is observed to be solvent accessible. The observation that F43 of the C1a domain is buried in an intramolecular interface with the kinase domain is consistent with a previously published report showing that its mutation results in accelerated membrane translocation of all conventional and novel PKCs (Lučić et al, JMB, 2016). Stabilization of the active conformation of the kinase domain and its intramolecular interactions by phosphorylation of T500, T641 and S660, however, was already known, including how mutation of these sites to non-phosphorylatable alanines causes PKC to partition into the insoluble or membrane-bound fraction.

Crystal structures of PKCbI reveal active and inactive states.

PKCbI and PKCbII are slice-variants that differ only in the C-terminal ~50 amino acid tail of their kinase domains. The authors report two more crystal structures, this time of PKCbI in two different crystal forms.

Crystal form 1 contains 2 mol/ASU in which one of the two chains is observed to have electron density for the PS in the catalytic cleft of the adjacent protomer, again via a domain swap. The arrangement of the regulatory domains in this crystal form seems to be incompatible with the inactive conformation that they report for PKCbII, and the authors investigate this arrangement later in the context of PKCbI activation.

Crystal form 2 contains 1 mol/ASU in which the PS segment is not observed bound to the catalytic cleft, despite it being unobstructed and available for engagement. The authors suggest that the packing of the regulatory domains with the kinase domain is similar to that observed in PKCbII, but this is difficult to assess based on the data presented. Visually, these structures do not appear to be that similar and the authors themselves state that the C1b domain occupies "an altered location…that we attribute to the autoinhibited conformation for the PKCbI enzyme." It is not clear what the authors mean by this, but it is difficult to imagine different autoinhibited conformations for PKCbI and PKCbII given that they are identical except for their C-terminal tails. Here, a definitive analysis of their claims is somewhat impeded by a more quantitative comparison of the PKCbII and PKCbI (crystal form 2) structures. Given that the PS segment is not observed in the catalytic cleft and the C1b domain occupies a different position, it should at least be considered that the arrangement

of the domains could be the consequence of lattice packing interactions that are energetically more favorable.

The authors observe an intriguing interface between the regulatory domains of PKC and its kinase domain that is common to both PKCbI structures that they have determined. In this conformation, which the authors refer to confusingly as N-term 1, the C1a, C1b and C2 domains pack against the C-lobe of the kinase domain. They make an interesting comparison to the AF2 prediction of PKCb, which places the domains in a similar arrangement. The authors have added in the AF2 prediction for the PKCa isoform, but there are still no PAE plots included in Supplementary Figure 5e, which would offer a metric-based assessment of the confidence of the predictions. Nevertheless, prediction of the same arrangement for other PKCs and the strong similarity to their experimentally determined structure strengthens the authors' claims.

PKCbI active conformation

The authors next investigate the interface between the N-terminal domains and the C-lobe of the kinase that they observed in both crystal forms and that is also predicted by AF2. Given that the AF2 predictions of PKCb and PKCa are convergent with the authors' experimentally determined arrangement of the regulatory domains, the implications are intriguing. Namely, that the membrane binding surfaces of the C1a, C1b, and C2 domains are observed in the same plane and binding of the PS segment to the kinase domain would be impossible. Should this structure represent the active conformation of PKCbI, it would help resolve two important questions regarding PKC activation: (1) does PKC form a defined, active conformation on the membrane (implications for substrate specificity etc) and (2) what prevents the PS segment from inhibiting activity on the membrane?

The authors attempt to validate this interface with a site-directed mutagenesis approach coupled to an in vitro FRET-based kinase assay (why such a complicated kinase assay readout is required in vitro is not clear – other, more classical in vitro assays would almost certainly provide a cleaner readout). They report a series of mutants in the observed C1b-kinase and C2-kinase interfaces that impair PKC activity in vitro. However, the effect is modest for all mutants, except for F286E, which (strangely) seems to exhibit no concentration-dependent change in activity (Figure 2C). Additionally, the observed packing arrangement of the domains exclusively involves the distal surface of the C-lobe of the kinase domain, which is not a reported surface of allosteric regulation for PKC. In fact, there are no movable elements in that surface that could regulate kinase activity allosterically. It is therefore hard to reconcile changes in activity in vitro caused by mutations that would disrupt these interfaces. The authors do not discuss this apparent contradiction, although, as previously stated, the interface they observe is potentially relevant. The question therefore arises: what is the interface useful for? In this respect, I believe that the authors may want to consider more explicitly the implications of the interface for the conformation and substrate specificity of PKC on the membrane. The consequences of such an interface for maintaining active and very highly specific PKC on the membrane due to a well-ordered conformation are quite profound, in my opinion, and are possibly the most interesting findings of this study.

I do <u>not</u>, therefore, dismiss a physiological relevance of the interface observed. Since the autoinhibited conformation of PKCb relies on the clamping of the PS segment by the C1b domain (Figure 1), a possible way to test this interface would be to examine the interface mutants in the context of a construct of PKCb lacking its PS segment. Kinase activity assays are unlikely to reveal allosteric changes for the aforementioned reasons, but the particle might reasonably be expected to remain compact and become extended upon disruption of the interface. This could be tested, for example, by SAXS analysis of PKCb interface mutants compared to PKCb, assuming that the recombinant proteins behave sufficiently well to make them amenable to such analyses.

The lipid lever mechanism of PKC activation

Having determined that the above conformation is, according to their mutagenesis data, biochemically relevant, the authors sought to rationalize the activation of PKC on membranes containing DAG. DAG-mimetics, such as phorbol esters, are commonly used to activate PKCs in vitro. However, as the authors show, they rely on being embedded in a membrane for their ability to activate PKC (Figure 3B). The authors assert that, due to solvent accessibility of the lipid binding surface of the C1a domain in their crystal structure, phorbol dibutyrate (PDBu; a DAG-mimetic) binding would not disrupt the inactive conformation and therefore a direct competition mechanism can be ruled out. I strongly disagree with this statement for the following reasons:

1. The PDBu-binding surface of the C1B domain in their structure is buried in an intramolecular interface, which means that PDBu would compete for this interface.
2. Multiple reports have unambiguously determined that the DAG-binding surfaces of the C1 domains (minimally one, but possibly both) are sequestered in the intramolecular, autoinhibited conformation. In fact, the naked C1 domains translocate to the plasma membrane with dramatically accelerated kinetics compared to full-length PKCs (Oancea & Meyer, Cell 1998; Lucic et al, JMB 2016).
3. Mutation of F43 (or its equivalent) in the C1a domain essentially unlocks all novel and conventional PKCs such that they translocate with near-identical kinetics to the isolated C1a-C1b domains. While an allosteric effect on the neighboring C1b domain and PS cannot be excluded, this observation strongly suggests that the DAG-binding surface of the C1a domain is also sequestered in the autoinhibited conformation. This raises the question of whether the interface observed in the authors' crystal structure is representative of the physiological conformation. Given the reported promiscuity of domain swaps in PKCb crystal lattice formation, this cannot be ignored.

The authors use a limited proteolysis assay to probe PKCbI conformation in the presence of Ca2+, PDBu or lipids, and combinations thereof. They show, in Figure 3C, that DAG-containing lipid micelles result in increased elastase-mediate cleavage of PKCbII (presumably in the C2-kinase domain linker?), whereas PDBu and Ca2+ are insufficient. This supports the requirement for DAG/PDBu to be embedded in a membrane/micelle, but it does <u>not</u> rule out a competition mechanism between DAG/PDBu and intramolecular contacts of the C1 domains with the kinase domain.

Minor comments related to Fig. 3C:

- Why do the authors compare PDBu with DAG-containing lipid micelles? This is a bit like comparing apples with oranges.

Minor comments regarding the text for this section:

The authors propose what they call a 'lipid lever model', in which they suggest that the membrane acts as a lever to pry the regulatory domains apart from the kinase domain. The model is based on the incompatibility of the domain arrangement observed in the autoinhibited structure of PKCbII with membrane binding by the C1a domain, which would cause a steric clash between the kinase domain and the membrane. Whilst the phrasing of this has been improved from the previous version of this manuscript, I urge the authors to reconsider their terminology here. If the C1a domain cannot approach the membrane because a of a clash between the membrane and the kinase domain, then binding is prohibited until the two domains dissociate (as the authors themselves write). As such, this model is not a 'lipid lever' model, which implies a force, but rather a process of conformational selection.

Although the authors use previously published membrane translocation data to support their lipid lever model, this is also problematic. In fact, published mutational data on F43, which appears to completely unlock the autoinhibited conformation of PKC (Lucic et al, JBC 2016), supports a conformational selection model in which the C1a and C1b domains must first dissociate from the kinase domain before they can bind the membrane. Here, whilst the authors have improved their terminology, their continued use of the 'lipid lever' terminology is misleading and their citation of references 14 and 17 needs to be more explicit. In particular:

1. It's not clear why reference 14 is cited after the sentence: "Additional data that supports the lipid-lever model comes from reports that the C1 domains exhibit enhanced membrane binding in the absence of the properly folded kinase domain 14, 17." Better references for this statement are the original studies by Oancea and Meyer (JBC 1998, Cell 1998).
2. The authors confusingly refer to the "absence of the properly folded kinase domain" in these studies, but what they mean is simply the absence of the kinase domain. They inadvertently imply that the proteins under investigation are not properly folded, which is not the case.
3. Reference 17 specifically deals with F43 and the equivalent residue in all conventional and novel PKCs. Since its mutation demonstrates accelerated membrane translocation of <u>all</u> cPKCs and nPKCs and the authors focus on it as part of their interface, it seems worthy of a more explicit explanation. In fact, this would help the authors to support their claims regarding the physiological relevance of the C1a-kinase interface they observe.

Molecular basis of isoform-specific differences between the splice variants PKCbI and PKCbII

PKCbI and PKCbII have been reported to have different affinities for phospholipid membranes. To try to rationalize this difference, the authors compared their structures of autoinhibited PKCbII (Figure 1) and PKCbI (crystal form I). The two structures differ in the following ways:

1. Electron density for the PS is only observed in the catalytic cleft of the neighboring protomer in the crystal lattice of PKCbI, an interaction brought about by a domain swap.
2. The C1b domain exhibits a different position: in PKCbII it is sequestered in an extensive contact with the catalytic domain that makes its lipid binding surface solvent inaccessible; in contrast, its lipid binding surface is solvent exposed in PKCbI.

According to the structure depicted in Figure 1D, the change in C1b position in PKCbI could reasonably be attributed to the absence of the PS in the catalytic cleft of one molecule in the ASU, an interaction which seems to be necessary for the intramolecular contacts of the C1b domain observed in PKCbII (Figure 1D). However, the authors do not appear to have considered this possibility. Instead, the authors focus on new contacts that they observe in PKCbI crystal form 1 between the C1b domain and the V5 extension to the kinase domain, which differs between PKCbI and bII. They postulate that the presence of F648 in PKCbI, which is V647 in bII, forces helix aB closer to the PS segment, thereby preventing an equivalent interaction of the C1b domain to that seen in PKCbII. To support this hypothesis, the authors engineered a F648A mutant of PKCbI and compared its sensitivity to elastase (as a probe for the active conformation) in a limited proteolysis assay. PKCbI was observed to be more sensitive to elastase cleavage than PKCbII in the presence of lipids. The following issues preclude a definitive assessment:

- It is unclear why the authors make a F648A mutant rather than F648V, which would convert F648 into the equivalent residue in PKCbII. As a test for the difference, this would seem to be a more appropriate comparison.
- n=1 for the data shown in Figure 4C. There is no quantifiable difference in elastase sensitivity in the absence of lipids (middle panel), while each protein measured in panel C is performed only once. What has been quantified by densitometry is also not described in the figure legend and the effect size is modest at best (6% of PKCbII cleaved vs 11% of PKCbI and 3% of the F648A mutant). Since this lacks replicates and corresponding error bars, it does not provide compelling evidence to support the authors' conclusions.
- This figure/data has not changed from the original manuscript.

Endoxifen as an allosteric regulator of PKCb

The final experimental section of this manuscript addresses published data reporting an inhibitory effect of tamoxifen (TAM) on PKC and a more potent effect of its metabolite, endoxifen (ENDX). The authors start by assessing the inhibitory effect of TAM and ENDX on PKCbI and bII in an in vitro kinase assay. Both compounds exhibited lower IC50 values in the absence of lipids; however, given the hydrophobicity of these compounds, indirect effects of lipids on their availability in solution cannot be ruled out. As such, the interpretation of the results in Figure 5A should be treated cautiously.

The authors also report thermal stability (DSF) measurements for PKCbI in the presence and absence of ENDX (Figure S7A). Although they claim that ENDX binds to both the N-terminal regulatory and kinase domains of PKCbI (pg 7, ln 15-17), the data do not support this conclusion. In fact, the melting temperature of full-length PKCbI (FL), the catalytic domain (CAT), and the N-terminal domain (NT) are unaffected (even slightly reduced) by ENDX (see table inset, Figure S7A). It is, therefore, not clear how the authors came to the conclusion that ENDX binds to PKC from this DSF data. The data and the conclusions from these experiments remain unchanged from the original manuscript, but do not support the authors conclusion that "differential scanning fluorimetry (DSF) analysis shows that both the N-terminal regulatory domain and the kinase domain bind to ENDX."

Next, the authors support the conclusion that ENDX is an allosteric inhibitor of PKCbI by comparing the effect of ENDX on PKCbI in the presence or absence of lipids with the isolated kinase domain of PKCb (Figure 5B). ENDX has a stronger inhibitory effect on PKCbI in the absence of lipids than either PKCbI with lipids or the isolated catalytic domain, which the authors interpret as an allosteric effect of ENDX on PKCbI that depends on the intramolecular assembly of its regulatory domains. (Again, I would emphasize the aforementioned concerns regarding the hydrophobicity of ENDX and possible (indirect) effects of lipids on its inhibitory behavior). This is further supported by kinase activity data in Figure 5C that shows that ENDX is a non-competitive ATP inhibitor (it lowers Vmax without changing the Km for ATP, right panel), which is in contrast to the known ATP-competitive inhibitor enzastaurin, which does not affect Vmax, but increases the Km for ATP (left panel).

Minor comment: panels A and C each contain two graphs, which should be labeled separately for ease of reading and interpretation.

To evaluate the effect of ENDX on the conformation of PKCbI in solution, the authors employed small angle X-ray scattering (SAXS). Figure 5D depicts the raw 2D scattering curves for PKCbI alone, +TAM and +ENDX, together with a table of the respective Rg and Dmax parameters derived from the data. The authors have added Guinier plots for their SAXS data (Supplementary Figure 7d) which confirm that their samples are generally well behaved, with the exception of their PKCbI+TAM sample, which shows evidence of low levels of aggregation (therefore Rg and Dmax should be treated cautiously). The manuscript also now contains a Materials and Methods section on the SAXS sample preparation and data collection, as well as a Supplementary Table summarizing the corresponding data parameters. Pair-distribution functions used to determine Dmax, however, are still missing. Overall, the evidence from SAXS that ENDX results in compaction of PKCbI is reasonably strong, as evidenced in the low-angle scattering regime in Figure 5D. However, the authors could make a stronger case by showing the respective Guinier plots for PKCbI ±TAM on the same plot in the main figure (only a SAXS expert will spot and appreciate the difference in the raw scattering curves that the authors show).

It is, however, somewhat perplexing that ENDX exerts an apparent compaction effect on PKCbI in solution, but has no stabilizing effect in the authors DSF assays (despite the authors' claims to the contrary. An allosteric inhibitor, that acts as a 'molecular glue' to stabilize intramolecular, interdomain interactions would be expected to have a fairly significant effect

on the melting temperature of FL PKCbI. However, according to the authors' data in Supplementary Figure 7a, it has the opposite effect of lowering the melting temperature. This apparent contradiction undermines the authors' claims that ENDX is an allosteric inhibitor of PKCbI.

Using limited proteolysis again as a readout for conformation, the authors then compare the effects of ENDX and TAM on elastase cleavage of PKCbI. No comparison to PKCbI in the absence of either compound is shown as a reference. While the data presented show a clear difference in the cleavage pattern which is visible by eye, it is not clear what sort of model has been used to fit the data in the lower panel. In particular, fitting the cleavage of PKCbI in the presence of TAM appears to be questionable given that there is no significant change at any concentration of TAM. This remains unchanged from the original manuscript.

Finally, the authors employ live-cell imaging to visualize the effect of ENDX on PKCbI translocation to the plasma membrane in MCF7AC1 cells treated with the phorbol ester PMA. The authors observed that ENDX promoted PKCbI translocation in the presence of PMA and that the PHLPP phosphatase inhibitor NSC117079 abrogated this effect (Figure 5F). Based on the authors' previous observations of the effects of ENDX, these findings are somewhat paradoxical. The authors claim that ENDX is an allosteric inhibitor of PKC that specifically targets its intramolecular conformation in an ATP non-competitive manner and renders it more compact. Intuitively, one might therefore expect an inhibitory effect on PKC translocation, much as has been observed for the allosteric inhibitor MK2206, which targets Akt. Instead, the authors observe the opposite. The data presented in Figure 5F and Figure S7C-D is difficult to assess for the following reasons:

- the authors present only static snapshots of different patches of cells in each experiment. Without translocation kinetic data, averaged over many cells to account for variabilities in PMA and/or ENDX availability that result from the available membrane surface area and local environment of each cell in a 2D culture monolayer, it is impossible to assess with any degree of confidence what the effect of ENDX treatment is on PKCbI membrane translocation.

The authors invoke the phosphatase PHLPP as a possible explanation for this effect on the basis that the reported PHLPP inhibitor NSC117079, as well as the PP1/PP2A inhibitor okadaic acid (OA), mitigated the increased localization. However, there are significant problems with this interpretation:

- The authors use a mutant PKCbI, T500A, which cannot be phosphorylated in its activation loop. ENDX, NSC and OA had no effect on the translocation of PKCbI T500A, compared to wt PKCbI. However, the implication, at least for NSC, is that PHLPP inhibition is responsible for this effect. However, PHLPP has been reported to be the phosphatase for the hydrophobic motif of PKC and not the activation loop (T500), and the validation of NSC as a specific PHLPP inhibitor is not very convincing, in the opinion of this reviewer. Furthermore, OA specifically inhibits a structurally different family of phosphatases to PHLPP, which implies that these compounds exert different effects on cells which cannot be rationally integrated into the same model.

- The authors reference autophosphorylation of T500, but the prevailing model in the field is that this is accomplished by PDK1. I do not necessarily subscribe to this model, but the authors should acknowledge it, since inhibition of autophosphorylation is intrinsic to their model. To support their model, the authors should demonstrate that ENDX inhibits PKCb T500 autophoshorylation in an in vitro kinase assay containing purified, recombinant, and dephosphorylated PKCbI. This should be possible given that the authors have well-behaved, purified proteins at their disposal.

Minor comment:

The authors reference Supplementary Figure 7C for the effects of OA on membrane accumulation, but the correct reference is Supplementary Figure 8C.

In summary, the assertion that ENDX is an allosteric inhibitor of PKCbI is not sufficiently well supported by the data the authors present.
